# Supplementary material for: Women’s experiences of communication with medical staff before and after emergency caesarean birth in Zambia: A qualitative study
Source: PLoS One. 2026 Apr 9;21(4):e0346694. doi: 10.1371/journal.pone.0346694 (PMC13065054; doi:10.1371/journal.pone.0346694)
Supplement: S1 File — (PDF) [file pone.0346694.s001.pdf]

Interview guide (women)

Part 1, Demography

1. **Gender**

   Female

2. **Age**

   18 to 20

   21 to 30

   31 to 40

   41 to 49

   50 and above (those who might have undergone in vitro fertilization)

3a. Education

What is the highest level of education that you have attained or are currently pursuing?

3b. Literacy

Are you able to read and write in any language?

3. **Occupation**

   What is your occupation?

4. **Religion**

   Which religion do you belong to?

5. **Marital status**

   Are you married?

6. Was this your first experience of the emergency caesarean section?

   Probe: If not the first emergency caesarean section, how many have you had?

7. What was the indications/cause for the caesarean section?

   Probe: If has had more than one emergency caesarean section, what lead to you having an emergency caesarean delivery?

8. What was your reaction when you were told you were going to deliver via caesarean section?

Probe: What do you think could have made you react that way?

9. What was your experience of communication before you went into theatre?

   Probe: What information were you given?

10. Do you think the information given to you was enough?

    Probe: If yes why do you think it was enough and if not why was the information not sufficient? What aspects of information would you have loved to receive?

11. Who provided this information on the need for emergency caesarean section?
12. Describe the process of consenting to emergency caesarean section?

    Probe: What did you make of it?

13. What factors did you consider when giving consent to emergency caesarean section?
14. Describe your experience of communication after the emergency caesarean section?
15. What information were you given?
16. What did you make of it?

    Probe: Do you think it was enough? If yes why do you think the information was enough and if no why do you think the information was not enough?

17. Which aspects of information would you have wished to receive?
