# Supplementary material for: Women’s experiences of communication with medical staff before and after emergency caesarean birth in Zambia: A qualitative study
Source: PLoS One. 2026 Apr 9;21(4):e0346694. doi: 10.1371/journal.pone.0346694 (PMC13065054; doi:10.1371/journal.pone.0346694)
Supplement: S2 File — (PDF) [file pone.0346694.s002.pdf]

Interview Guide for Health Care Providers

1. Who attends to women who need emergency caesarean section?
2. I understand medical doctors work in firms within the hospital, what are firms?
3. Where do the midwives fit in these firms?
4. Please explain to me what happens when a mother walks in or is brought to the UTHs Women and Newborn hospital up to theatre and after the operation until they are discharged.
5. What is the role of a midwife/Registrar/Consultant Obstetrician in obstetrics?
6. How would you describe communication between health care providers and the women who undergo emergency caesarean section?
7. What kind of information are women given before they undergo emergency caesarean section?
8. **Follow up question:** You mentioned that you only explain the indication for emergency caesarean section and the consequences of not consenting to the surgical procedure, why is that? Why is it that you only explain two things – indication for caesarean section and consequences?
9. Please describe the process of consenting to emergency caesarean section.
10. **Follow up question:** So who obtains this consent from the woman?
11. Please explain to me the communication between health care providers and women after surgery?
12. **Follow up question:** Are they given the consent form to read it on their own or it is read out to them?
13. What are some of the challenges that you face in communicating with the women that undergo emergency caesarean section?
14. **Follow up question:** Why do you think women don't understand how severe their condition is?
15. Earlier you mentioned that you only inform the women the indication for emergency caesarean section and consequences of not consenting to the surgical procedure due to limited time, what other factors apart from time do you think constrain medical personnel from fully communicating with the women who undergo emergency caesarean section?

16. In your opinion, what do you think is lacking in terms of communication between the two parties?

17. How much time is required to communicate to a woman who needs an emergency case?
