# Supplementary material for: Women’s experiences of communication with medical staff before and after emergency caesarean birth in Zambia: A qualitative study
Source: PLoS One. 2026 Apr 9;21(4):e0346694. doi: 10.1371/journal.pone.0346694 (PMC13065054; doi:10.1371/journal.pone.0346694)
Supplement: S5 File — (PDF) [file pone.0346694.s005.pdf]

ANONYMISED TRANSCRIBED DATA FOR FACE TO FACE IN DEPTH INTERVIEWS WITH WOMEN WHO UNDERWENT EMERGENCY CAESAREAN SECTION

PARTICIPANT NUMBER ONE

Age: [late 30s]

Highest level of Education: degree in [removed]

Literacy: able to read and write

Occupation: [removed]

Religion: Christian

Marital status: Married

Interviewer: Was this your first experience of emergency caesarean section?

Participant: No, this was the second time.

Interviewer: What was the cause of the first emergency caesarean section?

Participant: For the first emergency caesarean section my baby was coming out with face posterior/presentation though upon waiting, he wasn't adjusting, they had to rush me into C/section to take him out.

Interviewer: What was the cause for the second emergency caesarean section?

Participant: For the second emergency C/Section I had stopped dilating at 4cm. Also they waited but I wasn't dilated so I went for another C/section.

Interviewer: The first time you were told you were going to deliver through caesarean section, what was your reaction?

Participant: Shock first, then fear, lots of fear ah thoughts of my husband and kids because you never know ha ha ha ha how you come out from there. Yeah, so mostly shock and fear.

Interviewer: When you were attending antenatal were you told that you were going to deliver through caesarean section or it just happened?

Participant: Because my first delivery was through C/Section, my second delivery was a normal delivery ah I was expected to deliver normally with my third child. The antenatal everything was treated normally but I was a special case because I had previous C/Section. So I was told that there might be a possibility of delivering through C/section but there were no indications of complications that would lead to C/section so I was expecting to have a normal delivery. So no, I wasn't told like one hundred percent I was going to have C/Section.

Interviewer: The second time you had emergency caesarean section, what was your reaction?

Participant: The second caesarean section I literally broke down because I remembered how the procedure and process was in the first one and I think because of shock again I was thinking the worst would happen because for the first one I almost lost the child so for the second one, mentally I was telling myself that if nothing happens right now I might lose the child. So I literally had a break down, yeah.

Interviewer: What do you think could have made you react that way?

Participant: Ah I think, I am sure the medical personnel are trained in a certain way to handle, you know anxiety in women who are about to give birth in a complicated manner but I wasn't understanding the process at the time because they were more of laid back, relaxed, trying to observe without really hyping you, you know and I was in a panicking mood. So looking at them being like that and feeling the way I was feeling it just gave me more anxiety like they don't care about how I am feeling or the possibility of me losing my child. So it was a spiral of emotions you know. The way they were acting, the way I was feeling, nothing was really being communicated to me that you will be fine and all that. All that was being done was observation, no explanation, you know, so it is confusing ha ha ha yeah.

Interviewer: How would you describe the communication before you were taken into theatre?

Participant: Ah I can't say that I even knew why I was being taken to theatre, I just heard that she has stopped dilating, they were talking to themselves. No, she has stopped dilating I think we need to rush her to theatre. They didn't really explain why they had to take me to theatre. Sometimes these medical terms we all don't know them and when something is not being explained at the time, I think even if they tried to explain I don't really think I would have understood what they meant because all I was thinking about the baby's safety and my safety. So I can't really say there

was anyone who communicated to me that this is what is happening, we are going to take you here, all I knew was that the baby had to be removed as fast as possible.

Interviewer: What was going through your mind when the medical personnel were talking to themselves?

Participant: Ah like I was just an object ha ha like I was just an object on a bed, like I had no feelings, yeah like an object like I had no feelings whatsoever because this was my life they were talking about and I wasn't even involved in the process of delivery or being communicated to. So I just felt like an object, nothing much, just like an object.

Interviewer: Was that the same case when you had the first emergency caesarean section?

Participant: It was the same, it was the same. The first one they made me wait and again they were talking to themselves. I tried asking them what they meant about certain terms I had heard them mention but they would become a little bit frustrated because you would be asking too many questions, they think you can't understand the terms they would use. So they will just say just rest, just rest. So it was the same.

Interviewer: So they just got you wheeled into theatre without telling you that you are going for caesarean section?

Participant: They said you have to go for caesarean section and you have to sign this form. Now this form had a lot of writings on it and I am in pain I don't think I can even read it because all I am concentrating on is the pain I am having. So they tell you, you have to go for caesarean section and have to sign this form in case of anything. I can't even remember what was on that form or what it even meant me signing that form because they said the longer you take to sign this form, the longer you take to be wheeled into theatre and you haven't even read anything. So they don't tell you in detail, they just say sign the form.

Interviewer: What information were you given before you were taken to theatre?

Participant: I was just told you are having a C/section, we will give you anaesthesia, and then we have to wait because there is a queue, nothing else. Not that they are going to do this, they are going to do that. Just that.

Interviewer: Do you think that the information they gave you was enough?

Participant: It wasn't enough because I think to ease my nerves a little bit they should have explained that this is what is happening and we have to do this to save your life and the baby's life and the procedure will be just like this. Even just a few sentences just to put me at easy but that wasn't done. There was one male nurse who tried but even with him his trying I think because ok I had started crying at one point I think he felt very sorry for me and tried to assure me that everything was fine. I could see that doing that was taking time away from other patients so it's like it's just come and go, just come and go, so it's like they cannot give you any of the emotional part to easy you because it's taking away time from the patient on the next bed, so you are just there, you are just an object really.

Interviewer: What information would you have loved to receive?

Participant: First of all I would have loved to know how I was doing in the process of delivering the baby. Also secondly I would have loved to be assured that the baby was doing ok but that there was this emergency thing that needed to happen for both my life and the baby's life but none of that was really happening in the entire process**.** It's more of mechanical, like lie down, do this, and do that not really being reassured that you will be fine. So I would have loved to be reassured and just to be told a little bit of what was happening, just a little bit, that would have been fine.

Interviewer: What do you mean when you say it was mechanical?

Participant: Ah the procedures of how you are laying on the bed during the labour pains ah how you are shifted here and there you know you are told to get out of bed, told to sit there in terms of taking the vitals. There wasn't really that part of the emotional part going on or lack of a better word mechanical you know or sleep on the left side, sleep on the right side or sit up, sleep flat, let's check you know just those procedures.

Interviewer: You mean not being told what is happening?

Participant: Yes. It was quite an experience.

Interviewer: Who provided this information on the need for emergency caesarean section?

Participant: It was the nurse. I remember one of the nurses saying she has been at 4cm for seven hours, I think that was seven hours or something like that. She was telling the doctor and I

remember the doctor saying you can give them a sense of two minutes and they dilate fully. So if possible we could give her some more minutes but I was already mentally challenged thinking that the worst will come to the worst with my baby. So the nurses are the ones that said I think let us put her on the queue for C/section. So it was the nurses.

Interviewer: Please describe the process of consenting to emergency caesarean section?

Participant: I think the consent itself was with the signing of the paper because it is not like saying yes I am going for the C/Section. I was told that I have to go for the C/Section failure to which I may lose my life or the child may lose his life or it was literally just signing the paper. That was the only explanation given. So you sign it out of fear that something bad might happen to you or the baby or both.

Interviewer: What did you make of it?

Participant: I was upset really because I didn't know what I was signing. All I knew was that I had to put my signature on this paper for my baby and I to be ok. No explanation of the fine print. It's like a paper of one or two pages long with lots of words yeah**.**

Interviewer: What scenario would you have loved to see?

Participant: The scenario I would have loved to see because the entire time this was happening my husband was just outside the ward. The scenario I would have loved to see is if he (husband) was involved in the process knowing very well that I was already compromised physically, emotionally and mentally and even if things were being explained I don't think I would have gotten them. So I would have loved to see a situation where he is invited in and explained to him by the nurses so that he signs the consent form and he is aware of the risks and he knows what is happening instead of myself and other women were there within the vicinity but at least for them they had a sister or mother to sign the form for them. So that would have been better.

Interviewer: Why wasn't your husband allowed in?

Participant: Ah I don't know why he wasn't allowed in, I don't know why he wasn't allowed in because even when I was signing the consent form he was right outside the ward. I really don't know ha ha and I have never found out why. Maybe I should have asked but I don't know.

Interviewer: What factors did you consider when giving consent to emergency caesarean section?

Participant: Ah I was looking at how vulnerable I was and the distress I was in. I signed the consent form because I was in fear of my baby's life and mine and I was told the C/section needed to be done fast so I had to sign.

Interviewer: Describe your experience of communication after the emergency caesarean section?

Participant: ah ok this time around I think was a bit ok but then again it's like they are completely understaffed a lot of things are really not told to you after the C/section this is what will happen but of course you are told the eight hours bed rest. They don't really explain to you why you have to sleep flat for eight hours. You don't know why but they tell you it is important that you do this. I learnt the hard way with my first C/section. My child needed to be breast feed so one of my aunties who was at the bed side said that I sit up to breast feed. I did that, I think that is the worst thing I have ever done almost like in the fourth, fifth hour to the eight hours, then ah after that it was a spiral of events I started getting migraine headache, throwing up, …. I was expecting the nurses to explain to me why that was happening all they said is you sat up too quickly. What is happening? There was no explanation. It is only after so many years during my antenatal visit when I was carrying my second child that I was explained to that maybe it was the anaesthesia that was giving me those side effects but the doctor at that time explained to me that no that wasn't the side effect to anaesthesia or anything, the whole reason you are told to lay down in hours is for your spinal fluid to balance. So if you get up before that time you create imbalance which can lead to meningitis. This was not explained to me during my first time of having emergency caesarean section. So you can imagine the damage I would have done to myself. Just that piece of information, which they know and could have communicated. At least when I had the second C/section I knew I had to lay down for eight hours. Not all the women know that. Now you can imagine how many women leave the hospital without knowing that and you have to breastfeed the child all the time. But at least this time around I was in the high cost ward and they had formula or the nurses were the ones taking care of the baby and they had to give him formula. But those women who don't have formula and they make the mistake of sitting up and they are discharged and go home and they have these complications. It is would be nice to have information on how to take care of myself during that period including cleaning of the scars (wound) and everything. That you might experience this, your scar (wound) is going to heal like this you know just basic information.

Interviewer: Apart from being told to lay down for eight hours, what other information were you given?

Participant: Ah the other information of course the usual one after you have had a major operation don't eat solids, you have to walk immediately the eight hours is done ah you have to wash the wound just the basic information at least this time around it was better maybe because I was in high cost or what I don't know. Just the basic information on the upkeep of the wound. When to start eating solids, how to breast feed and all that while you have the surgery.

Interviewer: What did you make of the information they give after the emergency caesarean section?

Participant: Ah I would have loved medical personnel to be a bit more elaborate for a better understanding, a better care giving to myself you know so that I give the information to whoever will be taking care of me. It would be nice if it was more elaborate, a bit in detail even told the risks of not doing certain things they tell you to do but it is just listed one, two three no explanation. Maybe if you ask but you also have to ask in a certain way otherwise you will be shouted at and seen as if you are wasting their time.

Interviewer: Was that the same experience you had when you had your first emergency caesarean section?

Participant: The first time I had the emergency C/Section was worse, this is when I didn't receive all that information. So the first time was the worst because I remember after I was discharged I got migraines, I was throwing up and couldn't walk properly for nearly a month. The headaches just disappeared then I remember when I was pregnant with my second son in the second trimester, the headaches appeared, the same headaches then they just disappeared so I would not know if there was a deeper damage that was done. So from time to time the headaches do come back but they just disappear.

Interviewer: Which aspects of information would you have wished to receive after emergency caesarean section**?**

Participant: I think these ones are the ones I had mentioned, just the issue of how to take care of my body after emergency caesarean section in detail you know because this is not something that

happens every day. You are going to take care of your body when it is in different form so if the information was in more detail and explained properly it would be very nice instead of just little basic information, which makes it a bit complicated because you don't know how to deal with your body. So detailed information would have been very nice.

Interviewer: How would you want medical personnel to tailor information for first emergency caesarean section mothers, second and third?

Participant: I think the information needs to change because the first time I had the emergency caesarean section I healed fast and then the second time I had the emergency caesarean section, the healing was a little bit longer and a little bit more painful I don't know if that is normal so I ended up asking the doctor why there was a difference and I really had to ask. So his explanation was because we are opening the same wound the second time, we are re cutting the same wound so the healing won't be fast. So you can imagine if you go under the knife the third time, same incision you won't expect the healing to be as fast as the first one. So I think the information has to be tailored to what number of times you have been there you might think that certain things you were able to do back then which is not the case. So it has to be tailored to the number of times you have been there of course it should be similar because my first caesarean section I was up and about despite my migraine headaches. The second one took time.

Interviewer: What information were you given regarding future pregnancies?

Participant: For the first caesarean I was told to wait at least two years before I have my second child which we did. We waited two years by the time the second born was being born we waited a long time. Then for this other caesarean section we were being told after two years but we were being warned that we can't continue going under the knife but we can have as many as we want but you risk more complications because they are opening all the time you expose yourself to risks in terms of the bladder and other organs inside. So they were advising that that is why people go for three or four or more but you have to consider these risks of having every time you go under the knife.

Interviewer: As we conclude this interview is there anything of importance you would like to talk about regarding the communication between the women and medical personnel before and after emergency caesarean section?

Participant: I think the major one would be on the consent, signing the consent form at least if they could involve another party who would understand things better instead of asking this woman who is in distress, mentally, emotionally and physically challenged to sign something that they don't actually understand or better yet since you don't know that somebody will be undergoing emergency caesarean section maybe they can put it in such a way that any woman who comes through because you don't know they may have emergency caesarean section you explain these things beforehand because you don't know they might have an emergency c/section so you explain these things before hand before these things get complicated so that they know what they are signing and not when they are in distress you want them understand this document and sign. So maybe it should be mandatory and be introduced earlier during antenatal classes that consent form can be one of the things there so that even if you had like myself had nine months of healthy pregnancy but complications might arise during labour and you might have an emergency caesarean section. So if they can incorporate that in antenatal lessons that would be great because the women would know what they might be signing when you go there because it is very very important.

Interviewer: Participant number one thank you so much for your time.

Participant: Thank you for conducting this interview.

<u>PARTICIPANT NUMBER TWO</u>

Age: [early 30s]

Religion: Christian

Highest level of Education: Master's degree in [removed]

Marital status: married

Occupation: I am serving as [removed] at [removed]

Interviewer: Was this your first experience of emergency caesarean section?

Participant: Yes please.

Interviewer: How many children do you have?

Participant: This is my first child actually.

Interviewer: What was your reaction when you were told that you were going to have an emergency caesarean section?

Participant: Well ah I think the research itself stated the problem and that was the problem I found out. I knew it (caesarean section) was necessary but I think there were so many gaps especially where communication is concerned like you know working in a health system you expect some procedures to be done in a certain way. For instance, in a case of where you have to consent, it wasn't like an informed consent, it is like they were really pushing you, you have to do it so whether ah it didn't come with so much information and it didn't come with so much freedom to lay down yeah.

Interviewer: What was written on the consent form?

Participant: Ah well, what the procedure is all about. I didn't even have the time to read through it that is what the tricky is. I know it was an emergency but I think the health professionals or clinicians were supposed to even read it or summarize it but it is just where they give you, you need to sign this, it is an emergency. There was more of fear instilled in you. You are making a decision based on fear and not based on information really so yeah. I didn't have the time to read through.

Interviewer: Please describe to me the process of consenting for emergency caesarean section?

Participant: They just gave me the papers, actually I was told the baby may die if you don't go through caesarean section. So you see the information is coming with fear. So let's save the baby. I know it's an encouragement but what about if it came out to say this is the best alternative but we could do this, this and that, like give me options so that I can be able to choose but they came out like you are going to die or the baby is going to die if you don't do this. You see. So yeah I accepted, I was in panic mode because the information was more like an alarm and so when the forms came I just signed, they just said sign here without reading through and because I am a health professional it felt so wrong because whatever decision you make in a rush without really having to think about it, it shows that there is a problem somewhere. I lost the trust in the system, I lost the trust in the clinician I don't know if they were really for me or they just may be trying to have I don't know there was just mistrust involved. So you are getting in an emergency room scared not really that they are going to help me**.**

Interviewer: What information do you think would have calmed you down?

Participant: I think the whole consenting process if it is clearly stated and all the information is there. I know it is a necessity especially in the condition I was under but you don't approach it like instilling fear into someone because I was getting there even when things were not okay I just felt like okay this is a 50 – 50 thing. I accepted it is the only thing that can be done but do I really have to trust the system if things don't go well? You know the mistrust I was talking to you about, there was no trust and it wasn't a very nice experience.

Interviewer: Who gave you the consent form?

Participant: Ha ha ha I won't tell you the name.

Interviewer: Was it the nurse or doctor?

Participant: A nurse and apparently she is a good person but you see it is the system, which is set in place where they don't think it is important to have proper consent. The panic yeah it is for the sake of the baby yeah but give information, it is that information that will calm the person down, it is that information that will make the person have a positive attitude when they go into that emergency room so in as much as you can be medically right, is the person ready, emotionally ready for the process and it being my first experience of emergency caesarean section I didn't know what was going on the next time I didn't know what even the consent form I expected it to outline the process, from here we are going to do this and that but it's just like you are a rat or a rubber, they just throw you here and there and with so much mistrust which is there and it's like you are just giving yourself for some sort of an experiment yeah and you know how it is, you even know that if anything was to go wrong they wouldn't even be accountable because apparently that is the system in place. So yeah it was scary, very scary**.**

Interviewer: What factors did you consider before signing the form?

Participant: just the safety of the baby you know when the mother's instinct just comes in you just want the baby to come out safe. I think that was the major, it wasn't even about me my safety yeah the mistrust was there but should it come at the cost of the baby's life? So the biggest factor was just survival of the fittest.

Interviewer: What was the indication for emergency caesarean section?

Participant: What do you mean?

Interviewer: What was the cause of the emergency caesarean section?

Participant: oh ok. They said fetal distress so when I came to the hospital my pregnancy was overdue. One of the consequences of that is that balanaka, they (baby) get tired in the stomach so when I was coming here the baby was already gone into fetal distress. So as they were getting the vitals the water broke we could see an indication that the child had pooped inside so it was definitely a caesarean case yeah. I know it was necessary I know that is the only way I could have given birth but let there be a proper consenting process especially for people who don't know how the process goes just for the sake of calming the mind of the person who is going into theatre.

Interviewer: How would you describe the communication with medical personnel before you were taken into theatre?

Participant: I encountered a number of them, it was a chain of them but I think it was the consultant who informed me that I was going to have emergency caesarean section. She was working hard in hand with the midwife. So with the consultant like I said it is something that they do she just said this is a caesarean case which I understood because I am a health practitioner as well but where I had issues is the consenting process. So the one who brought the consent form was the midwife. So you can see the information gaps from the midwife's point of view but I was made to sign, sign here, do this, do that and because you are running out of time for the baby just sign it but you just accept the communication gaps and do what they are telling you to do for the sake of the child.

Interviewer: How did that make you feel?

Participant: I had a mixture of feelings – hopeful and fearful you know where it's no longer about you anymore but the baby. The mistrust was there but you hope for the best. I don't go to private facilities because of the mistrust because you don't know if they are interested in your money or your general health even when you go to, I stay in [removed] by the way in [removed] you go there you go to the government hospital but it is the same gap of information then there is a gap of professionalism then they only have one person. So when I came here it's like I was expecting you are told it's never a straight line when it comes to these issues. It's like I had prepared myself

already to say just in case it happens the odds of a successful operation but they could do better when it comes to consenting.

Interviewer: Apart from information on the consent form, for you to make an informed decision, what other information would you have loved to receive before you were wheeled into theatre?

Participant: I think basically everything falls into the consent form, I know it is a summary, I don't know I have forgotten how to write a consent form but streamlining the procedure and I think that is consenting you have to make somebody understand what they are getting themselves into and you can even mention the risks which are there how they should behave, you can mention the odds of a successful emergency caesarean section because I didn't even know anything about the procedure, how much risk do I have, is it possible that it might not be successful, I didn't even have that information you know so if I who is a health professional don't know what more somebody for instance these guys from rural areas who have never even gone to school? You are just at the mercy of health care providers, you are just being experimented on you know, had I been on the upper hand it is not something I would really advocate for. Professionalism they are good, they know what they are doing but they can't speak it out. They are practicing on you. But otherwise I trust [hospital removed] but I may not have trust in the individuals that are there.

Interviewer: Describe the communication experience with health care providers after emergency caesarean section?

Participant: I think that is where it was even bad because I was just left alone. They didn't tell me why I am left alone, they didn't tell me what I was waiting for. Emergency caesarean section is not a pleasant procedure. They say no you won't feel the pain but there are these reactions you feel even just the feeling of numbness at the back of the mind you know somebody is cutting on you, effects which come with that like feeling cold they needed to explain all that so that I don't have to be anxious. When it comes to the health of the person when you are scared even when it was supposed to be a minor effect it can be a major effect, the shivering because I kept on asking is this normal, why because I needed to know so that I calm myself down to say this is the actual procedure this is what everybody is going through but in a case where by they don't tell me anything its actually normal but because I don't have all that information so after the procedure there was no one to explain anything and one thing I noticed the doctors were more interested in the procedure than actually the person they are operating on. I understand it is exciting, it is the

love of medicine this weakness of playing God, it is like they are more interested in the actual process than the wellbeing of the person they are processing on. I remember after me there was a case which was more severe than mine that is why they had to leave the room I was in to go and observe the more critical case so that they learn you get the point so that they learn more since most of them were student doctors. You can see the focus is not really on the person, it is on the actual medical practice, which makes it further more difficult for trust even if they are good at it but do you have me at your best interest? Ha ha ha it is crazy, they are good at what they do, I was supposed to do medical school from here but I decided to do [removed].

Interviewer: It is not too late you can still study medicine.

Participant: Ha ha ha no no I think [removed] is fine for me. I wanted to do it. I love the interest but I know most of my colleagues who have done it, it is the same issue of the God factor they are more interested in**.** The medical practitioners are great in what they do but they lack the psychological aspect, which needs to be addressed yeah.

Interviewer: Were you given an opportunity to ask questions before and after the procedure?

Participant: Yeah a few instances like the anaesthesia the guy who was doing that explained to me so we are going to do this and that okay he summarized it but you know consenting like I told you is a continuous process and there is times when something odd happens then you ask you know maybe it was me, maybe I was expecting more information compared to others but it has to be inclusive I who need more information I have to be answered to these other guys maybe they don’t need it because they don’t know or they may not understand but I can understand it for me I think I can understand anything you can just explain it to me so you would find that with that summary they gave they thought it was enough I kept on asking questions and you could see they were irritated so even me I equally got irritated the fact that they were irritated but now the focus was okay let me keep quiet it is not about me let me just be hopeful and have this baby just come out.

Interviewer: As we conclude this interview, is there anything else that you would like to talk about regarding the aspect of communication that you haven’t mentioned already?

Participant: I think I have mentioned everything yeah the trust of the system can be there like was the case with me I was able to make that decision to come to [hospital name removed]. Based

on my experience since I have never undergone any operation in my life so I expected something better, I expected something less stressful because I was even surprised by how long it took, they didn't even tell me how long the procedure is, I didn't even know I was finishing my whole process together with someone who was next to me because it was interesting so they had to make me wait longer because they have to attend to the next patient, you know all those loopholes yeah I trust the institution because it is[text removed] something like that but the individuals in it I think they can do better especially the communication aspect, you know when you are at the mercy of a professional if you think who is this person, how much information do they need, how much information can you comprehend so they have to go with the person's comprehension levels. You can't explain a procedure to me like you would explain to a grade seven you have to at least bring it to the level of my understanding so that I can comprehend. I got the baby but actually I didn't like the experience of communication because I feel it could have been done better.

Interviewer: What information gaps did you notice?

Participant: The whole process, explaining it by point. You can even do it as you are suturing I think that that is the main reason why all your senses are on the sense of feeling why because you want to walk the person through. That is one of the objectives but if you keep quiet and sometimes they are talking to one another but not talking to you it makes you feel more of an object, you know objectifying you, when they are supposed to explain to me yeah so in short it is the process that I didn't like but I trust the institution. They can do better where communication is concerned. They can actually see that maybe most of the nurses don't even understand what is going on but they have left it to the doctor that he or she knows what he or she is doing but the one who is closest to the patient is actually the nurse because they have one on one contact with the nurse so even the nurses should be interested in the procedure than them just running up and down if they can't explain it to a lay man then they don't know what they are doing. So I think the information gaps start with them than the processes themselves because if you really understand the process then you can explain it and they have that gap between the nurse and doctor where the doctor is just there suturing, I think there is an imbalance I know it is professionalism but it is a wrong culture, there should be that fluidity information flow from the doctor to the midwife, midwife to the person who is on the table. There shouldn't be information gaps yeah.

Interviewer: Anything else?

Participant: Now I am scared of having another baby because I don’t trust the system ha ha ha but other than that I am ok.

Interviewer: Thank you so much for your time.

Participant: You are welcome**.**

PARTICIPANT NUMBER THREE

Age: [late 30s]

Highest level of Education: degree

Religion: Christian

Occupation: Teacher

Marital status: Married

Interviewer: Was this your first experience of emergency caesarean section?

Participant: Yes.

Interviewer: What was the cause of the emergency caesarean section?

Participant: The baby was not dilating. The other thing is that labour was not progressing.

Interviewer: What was your reaction when you were told that you were going to deliver via caesarean section?

Participant: It was traumatizing of course and stressful.

Interviewer: What do you think could have made you react that way?

Participant: Fear, just fear.

Interviewer: What was your experience of the communication before you were taken into theatre?

Participant: No response

Interviewer: What information were you given before being taken into theatre?

Participant: the information I was given was about the reasons for C/section so that I should be aware why they are taking me that side.

Interviewer: What did you make of the information you were given?

Participant: I think it was the right decision kaili I am not a medical person. They should know better so I just have to follow what they tell me.

Interviewer: Do you think the information you were given was enough?

Participant: Yes

Interviewer: they told me everything that I needed.

Interviewer: What aspects of information would you have loved to receive?

Participant: Like is the caesarean section safe for me and the baby what will come of me after the caesarean section.

Interviewer: Who provided this information to you on the need to have emergency caesarean section?

Participant: The midwife and the people who were conducting the caesarean section.

Interviewer: the doctor or nurse?

Participant: The doctor.

Interviewer: Please describe the process of consenting to emergency caesarean section?

Participant: It is not an easy thing to do but after hearing the advice.

Interviewer: Were you given any form to sign?

Participant: Yes there was.

Interviewer: What was on the consent form?

Participant: just the consent form if I agree to be operated on.

Interviewer: What was the content of the consent form?

Participant: full names, age and if I agree to be operated on.

Interviewer: Did you understand the consent form?

Participant: Yes.

Interviewer: What did you make of it?

Participant: next question

Interviewer: What factors did you consider when giving consent to emergency caesarean section?

Participant: The safety of the baby and my safety.

Interviewer: Describe your experience of communication after emergency caesarean section?

Participant: It was ok.

Interviewer: What was ok about it?

Participant: No response

Interviewer: What information were you given after the caesarean section?

Participant: just on how to take care of the wound.

Interviewer: Apart from giving you information on how to take care of your wound, what else did they tell you?

Participant: Just how to take care of the wound so that it heals.

Interviewer: What did you make of the information they gave you?

Participant: It was enough.

Interviewer: What other aspects of information would you have loved to receive?

Participant: nothing

Interviewer: How many children do you have?

Participant: I have three.

Interviewer: As we conclude the interview is there anything of importance that you would like to talk about before and after emergency caesarean section?

Participant: before you feel scared because you don’t know what you are going through and after you feel comfortable because you are relieved.

Interviewer: I would like to sincerely thank you for participating in the interview.

Participant: You are welcome.

PARTICIPANT NUMBER FOUR

Age: (mid 30s)

Highest level of Education: Grade seven (primary school education)

Literacy: not able to read and write

Occupation: Nothing

Religion: Christian

Marital status: Married

Interviewer: Was this the first operation?

Participant: No, this is the third one**.**

Interviewer: All the three were emergency caesarean sections?

Participant: No, the third one is the one, which was an emergency. The other two I came to deliver from the hospital. My first pregnancy, the baby was big so they told me I have to be operated upon. He was 4.7 kg. Then the rest of the two children were weighing 2.3 kg. I have six children.

Interviewer: What was the cause of the emergency caesarean section?

Participant: I started bleeding from home in the morning and when I noticed blood clots around 19hrs, I rushed to the (hospital name removed) where the nurses examined me and advised that I needed to be referred to the (Hospital name removed). They scolded me for not seeking medical attention much earlier considering that I had two caesarean sections previously. The nurses informed me that my HB was low and that they couldn’t operate me because there was no blood in case of a transfusion.

Interviewer: What was your reaction when you were told you would deliver via caesarean section again?

Participant: I somehow had an idea that I was going to be operated upon because I have had two operations already and during antenatal I had requested that I should have a bilateral tubal ligation (BTL). So when I was taken to theatre I thought they were going to perform a BTL, I didn't know that I was going for caesarean section.

Interviewer: What information were you given before being taken to theatre?

Participant: they gave me a form to sign and I signed. They just told me that there was something wrong with the placenta I can't remember the medical term they used**.**

Interviewer: What was written on the consent form?

Participant: what was on the consent form was the part where I had to write my name.

Interviewer: What else was on the consent form?

Participant: I can't remember.

Interviewer: What was your experience of communication with medical personnel before you went into theatre?

Participant: They made feel comfortable and I could see that it was really an emergency because the doctors were running around. Even in the theatre the doctor kept reassuring me that I will be fine. Apart from trying to reassure me the doctor was trying to strike a conversation like how old I am just to allay my fears.

Interviewer: What information were you given before the operation?

Participant: I was told that I didn't have enough blood that is why I was going for an operation.

Interviewer: Why did you sign the consent form?

Participant: I signed because I wanted to save the baby's life and mine**.**

Interviewer: What information were given after C/section?

Participant: they only told me that the baby‘s temperature was high and that they had taken him to D block (Neonatal Intensive Care Unit)**.**

Interviewer: Who provided the information to you?

Participant: Student nurses who wore stripped attire gave me advice on how to take care of my wound and not to eat solids for a certain period of time.

Interviewer: What aspects of information would you have loved to receive?

Participant: everything I was told was just ok.

Interviewer: Thank you for your time.

Participant: You are welcome.

PARTICIPANT NUMBER FIVE

Age: (mid 20s)

Highest level of Education: Diploma

Literacy: Yes

Occupation: Teacher but not yet employed

Religion: Christian

Marital status: Married

Interviewer: Was this your first experience of emergency caesarean section?

Participant: Yes, it was.

Interviewer: How many children do you have?

Participant: This is my first child.

Interviewer: What was the cause of your emergency caesarean section?

Participant: The baby was big and the centimetres were not improving because of my height and everything.

Interviewer: What was your reaction when you were told you were going to deliver through caesarean section?

Participant: I was okay, I think I was prepared for anything. It didn't come as a surprise.

Interviewer: Why didn't it come as a surprise to you**?**

Participant: My last visit during antenatal I was told the baby was big so three weeks after that I knew that the baby must have grown more than that. So I was already prepared for anything.

Interviewer: What was your experience of communication with medical staff before you went into theatre?

Participant: It was ok.

Interviewer: What information were you given before you were taken into theatre?

Participant: I was just told to go for caesarean section and then come back. That's it.

Interviewer: Do you think that the information you were given was enough?

Participant: Yes, ok they asked me if I was prepared and I said yes. So I think the information I had was enough.

Interviewer: What aspects of information would you have loved to receive?

Participant: I think the information was enough. I wouldn't have wanted more.

Interviewer: What factors did you consider when giving consent for emergency caesarean section?

Participant: The factors I considered were to have a normal baby because if I had delayed maybe the baby would have been tired. I looked at the wellbeing of the child, yes mum.

Interviewer: Please describe your experience of communication with medical staff after the operation?

Participant: The experience I had was awesome. They managed to bring the child to me after I regained conscious and was able to take care of her.

Interviewer: Then what was your experience of communication with medical staff before the operation?

Participant: Ah it was ok.

Interviewer: What information were you given after the operation?

Participant: They have been telling me about how I am progressing - temperature, blood and blood pressure.

Interviewer: Apart from that is there any information that they have given you?

Participant: No, for now not yet since I am still in their (nurses and doctors) care.

Interviewer: As we conclude this interview is there anything of importance that you would like to talk about regarding communication with medical personnel before and after emergency caesarean section?

Participant: The only thing is that they have really helped and I would like to appreciate them a lot.

Interviewer: Thank you so much for your time.

Participant: Thank you.

PARTICIPANT NUMBER SIX

Age: (mid 20s)

Highest level of Education: Grade seven (primary school education)

Literacy: I don’t know how to read but I can write**.**

Occupation: nothing

Religion: Christian

Marital status: Married

Interviewer: Was this your first emergency caesarean section?

Participant: It’s the second one.

Interviewer: What was the cause of your emergency c/section?

Participant: The first emergency caesarean section was done in [name of place removed] in 2014. Then I had the second emergency caesarean section here.

Interviewer: So how did it go?

Participant: it went well.

Interviewer: What caused the first emergency caesarean section?

Participant: The baby was too big. The baby was too too big.

Interviewer: Then what caused the second emergency caesarean section?

Participant: I was bleeding heavily from 20 hours to 01 hours so medical staff said I was going to deliver through caesarean section again.

Interviewer: What was your reaction when you were told you would deliver through caesarean section again?

Participant: I had to listen to what the doctor said that the bleeding was too much.

Interviewer: What do you think made you not to be scared to be operated upon?

Participant: It was painful so I had to be strong.

Interviewer: How would you describe the communication with medical staff before you were taken into theatre?

Participant: it was just ok. They kept on checking how far I was in labour.

Interviewer: What advice were you given or what did they tell you before you were wheeled into theatre?

Participant: There is nothing they told me.

Interviewer: So they just took you to theatre?

Participant: Yes, they just wheeled me into theatre and called someone to come and conduct the operation.

Interviewer: In your opinion, do you think the information you were given was enough or what information would you have loved to receive from medical staff before you went into theatre?

Participant: Nothing, I don't want to lie.

Interviewer: Who gave you the information?

Participant: I don't know his name.

Interviewer: Was it a nurse or doctor?

Participant: It was the doctor.

Interviewer: please describe your experience of communication with medical staff after emergency caesarean section?

Participant: There was nothing they told me. I was just wheeled back to the ward.

Interviewer: What kind of information would you want medical staff to be giving mothers?

Participant: I don't know.

Interviewer: Thank you for your time.

Participant: you are welcome

PARTICIPANT NUMBER SEVEN

Age: (early 30s)**.**

Highest level of Education: Grade 12

Literacy: Yes.

Occupation: Business lady

Religion: Christian

Marital status: married

Interviewer: Was this your first experience of emergency caesarean section?

Participant: Yes**.**

Interviewer: How many children do you have**?**

Participant: Three.

Interviewer: What was your reaction when you were told that you were going to deliver via caesarean section?

Participant: nothing because I expected it because my second child was delivered via caesarean section so I knew that even the third child would be born through c/section. So I was just fine.

Interviewer: So what was your experience of communication with medical personnel before you went into theatre?

Participant: It was fine.

Interviewer: What was fine about it?

Participant: I remained calm so that I don't cause any problems in theatre.

Interviewer: What information were you given before you were taken into theatre?

Participant: they told me I was going to be taken to theatre because I couldn't deliver in a normal way. I told them that I know and that they should take me to theatre for the sake of my child.

Interviewer: Do you think that the information you were given was enough?

Participant: It was fine according to the situation I was in.

Interviewer: What aspects of information would you have loved to receive?

Participant: Silence

Interviewer: What other information would you have loved to receive before being taken into theatre?

Participant: Nothing that was enough.

Interviewer: Please describe the process of consenting to emergency caesarean section

Participant: nothing.

Interviewer: Were you made to sign any form?

Participant: yes I did. I signed one paper. I just put the details of my family that's all.

Interviewer: what was written on the form?

Participant: The form was just asking me to agree on everything. The other thing was the phone numbers for the next of kin so that if there is any mistake they (medical staff) can inform him (husband).

Interviewer: So what factors lead you to signing the consent form?

Participant: So that they can have the information.

Interviewer: ok, what are the reasons you signed the consent form?

Participant: You know when you are going into theatre bad things can happen so it is important that they have contact numbers for your relatives – in case of anything they let them know.

Interviewer: What was your experience of communication with medical staff after the operation?

Participant: it was just fine. I told them I was feeling pain and they gave me some medicine.

Interviewer: What information or advice did they give you**?**

Participant: They told me to clean the wound and warned me that I risked over staying in the hospital if I felt lazy to clean my wound. So I have just been following their instructions.

Interviewer: Apart from information on how to take care of the wound, what else have they told you?

Participant: They told me that if I experience any pain after discharge I should return to the hospital. I shouldn't wait too long but come back to the hospital so that they see what happened to me.

Interviewer: is there anything else**?**

Participant: No.

Interviewer: What else would you have loved them to tell you?

Participant: nothing. I just want to thank them for the good job they are doing. I was in pain for five days and if it weren't for them I would be dead together with my son.

Interviewer: Thank you so much for your time.

Participant: you are welcome.

PARTICIPANT NUMBER EIGHT

Age: (mid 30s).

Highest level of Education: Grade eight

Literacy: I am able to read and write

Occupation: [removed]

Religion: Christian

Marital status: married

Interviewer: Was this your first experience of emergency caesarean section?

Participant: yes

Interviewer: How many children do you have?

Participant: three

Interviewer: What was your reaction when you were told you were going to give birth via caesarean section?

Participant: I was scared ha ha because it is my first time. So I was very scared ha **ha.**

Interviewer: Why were you scared?

Participant: I was scared because the other two children I delivered them normally. I was scared because it was my first time.

Interviewer: What was your experience of communication with the medical personnel before you were taken into theatre?

Participant: Ah ok it wasn’t good because some of them were shouting at me over things that I did not even understand. There were some things that I did not understand but I was surprised that I was shouted at. But some of the medical staff were understanding.

Interviewer: What information were you given before going into theatre?

Participant: they only told me to sign the consent form an indication that I had agreed to undergo emergency caesarean section. Then they told me that the reason for caesarean section was because the baby's weight was big.

Interviewer: Do you think the information given to you was enough before you went into theatre**?**

Participant: It wasn't enough because they didn't give me a proper explanation.

Interviewer: what kind of information would you have loved to receive**?**

Participant: At least they should have explained how things were, the process of the procedure but they just came and said sign the consent form, you are going into theatre and told me the reason for emergency caesarean section that the child was overweight**.**

Interviewer: What were the contents of the consent form?

Participant: there was no time to read the consent form. All I was told was to write my name, where I am coming from and appending my signature.

Interviewer: What made you sign the consent form**?**

Participant: because they told me that every person going to theatre is required to sign the consent form.

Interviewer: Please describe to me the process of consenting to emergency caesarean section

Participant: hmmm

Interviewer: the process of agreeing to go into theatre, describe to me how it's like

Participant: I just agreed because I had no choice, they told me that the baby was too big and that the only option I had was to undergo emergency caesarean section. That is why I agreed.

Interviewer: Describe your experience of communication with medical staff after emergency caesarean section

Participant: it wasn't bad because they took us to the waiting room and in the morning took us to the ward.

Interviewer: What information have you been given after coming from theatre?

Participant: They told us how long we were supposed to lie down, drink water to avoid complications.

Interviewer: Apart from that is there anything else you were told?

Participant: nothing

Interviewer: do you think the information you were given was enough?

Participant: hmmm I can't say it was enough or not because I have no idea how things are supposed to be. So I just followed the instructions they gave me.

Interviewer: What kind of information would you have loved to receive after the operation?

Participant: I would want them to advise me on how I should take care of myself so that I am safe.

Interviewer: Did they advise you on when you can have your next child?

Participant: Not yet.

Interviewer: is there anything else you would want to talk about communication with medical personnel before and after emergency caesarean section, something you haven't mentioned already?

Participant: ok medical staff differ, others are good and others are bad. But it is important for them to explain things properly when they are asked rather than shouting at the patient. Some of us don't understand medical issues because it is not in our line of work. So it is better they explain instead of shouting. Like in my case this was my first emergency caesarean section but they shouted at me on things that I didn't even know or understand.

Interviewer: Thank you so much for your time.

Participant: You are welcome.

PARTICIPANT NUMBER NINE

Age: (early 30s).

Highest level of Education: Grade nine

Literacy: Able to read and write

Occupation: Business lady

Religion: Christian

Marital status: Married

Interviewer: Was this your first experience of emergency caesarean section?

Participant: No, this is the second time I am having unplanned operation.

Interviewer: What lead you to having emergency caesarian section the first time?

Participant: The first born it was breech and again this one the cause is breech.

Interviewer: When did you have your first baby?

Participant: In 2005.

Interviewer: What was your reaction when you were told that you would again deliver the baby through caesarean section?

Participant: I felt very sad and bad because my first child was delivered through an emergency caesarean section. I have four children. The second and third were normal deliveries. The fourth one was an operation again. Anyway, I just prayed to God.

Interviewer: What do you think made you react like that?

Participant: I felt sad and prayed to God because most people don't survive the caesarean section especially when they sign the consent form. Before you have an operation you have to sign a consent form so that if anything happens to you, the doctors are not held responsible. You just have to rely on faith to survive.

Interviewer: What was your experience of communication with medical personnel before you went into theatre?

Participant: I first went to (facility name removed) when labour pains started. At the clinic they noticed that the baby was not fine, one leg was coming out so they told me to go and pack my

things and that the ambulance was waiting for me. So I was referred to [hospital name removed] and when I got here I was taken straight into theatre. Medical personnel asked me if there were some family members that had accompanied me but I was alone. I was made to sign the consent form as I was being wheeled into theatre. They treated me well until I felt numb on the lower part of my body.

Interviewer: What information were you given before being taken into theatre?

Participant: They didn't tell me anything. They just examined me and quickly took me to the theatre.

Interviewer: What did the medical staff tell you before they took you to the theatre?

Participant: Ok but when I was taken to theatre I didn't know they were going to operate on me. I thought they were going to turn the baby and put it into the right position. A lot of people say when the baby is breech, medical staff can turn the baby and put it in the right position. They didn't tell me I was going to have an operation. I honestly had no idea it was an operation. So when I was in theatre I asked if it was an operation. Then medical staff were surprised. They asked me "you haven't been told it's an operation?" I responded, no, they didn't tell me anything.

Interviewer: So then who told you that you were going to have an operation?

Participant: The people in the theatre are the ones who told me that I had been taken there for an operation.

Interviewer: Were they nurses or doctors?

Participant: Mmmm there are no nurses in theatre I think it was ah the trainees, abalumendo nabakashana (some males and females).

Interviewer: Earlier, you told me you were given a paper (consent form) to sign. Kindly explain to me the process of consenting to emergency caesarean section.

Participant: They just gave me a paper (consent form) when I was lying down on the bed and told me that I must sign. So I just signed the consent form fast fast.

Interviewer: So what was written on the consent form?

Participant: I was rushed into signing the consent form. So I just appended my signature.

Interviewer: So you didn't read the consent form or it wasn't read to you?

Participant: No, they didn't give me chance to go through consent form. They said I must sign. So I just signed the consent form.

Interviewer: So what did you write on it?

Participant: I just appended my signature. That's all.

Interviewer: What factors did you consider when consenting to emergency caesarean section?

Participant: They said I should sign so that in case I die they can say I agreed to be operated on.

Interviewer: Ok, what else made you sign?

Participant: the other reason is that I was going for an operation and it is a requirement that I do so.

Interviewer: What was your experience of communication with medical personnel after the operation?

Participant: The nurses encouraged me to be prayerful following surviving the operation. They also told me not to lift heavy things when I get discharged. They also told me that this operation was different from the first one. They also gave me advice on how to clean the wound with cold water and soap.

Interviewer: What else did the medical staff tell you?

Participant: They also told me not to eat solid foods for a particular period of time.

Interviewer: In future do you intend to have another baby?

Participant: I doubt it. Since I have two girls and two boys, they are enough but anyway I have to speak with my husband first.

Interviewer: What other aspects of information would you have loved to receive?

Participant: During the first emergency caesarean section they told me to wait until the wound healed before I could fall pregnant again – two to three years to avoid complications.

Interviewer: What other aspects of information would you have loved to receive?

Participant: Ok I would have loved if they could give me information on when I can resume sexual intercourse.

Interviewer: As we conclude this interview, is there anything else you would like to say that you haven't mentioned already?

Participant: I just want to say nurses and doctors should respect patients. We see other patients being disrespected and shouted at as a result a patient can die from high blood pressure. I would have loved to be given information about the welfare of my baby. I kept on asking them and they kept saying the baby was in the room. They took so long to bring the baby to me so I thought it had died.

Interviewer: Thank you so much for your time.

Participant: I am also grateful.

PARTICIPANT NUMBER 10

Age: [early 40s]

Highest level of Education: Diploma in Accountancy

Religion: Christian

Marital status: Married

Interviewer: Was this your first emergency caesarean section?

Participant: Yes, it was. I have had normal deliveries except for this one. The reason being that I was told the baby had too much weight which I couldn't deliver normally. So they decided to take me for C/Section, which was the safest way for me and the baby.

Interviewer: What was your reaction when you were told that you were going to deliver through caesarean section?

Participant: At first I wasn't comfortable with it knowing what comes with it having a sore afterwards. Then it would entail that I would be looking after myself and the baby for quite some time. Then the other thing I was concerned about is being an active person it would mean that I would be inactive for some months because I will not be allowed to carry heavy things. But I had

to accept for the sake of life, life is life - preserving my own life and that of the baby. It is by the grace of God that they chose this path for me, I did not chose it for myself.

Interviewer: What do you think could have made you react that way?

Participant: There are so many stories we hear about people who go through c/section. Others will tell you there are complications. But after the doctor explained that I will just lead a normal life just like any other person who is going through a normal delivery I had to accept it.

Interviewer: So how many children do you have?

Participant. Four. This is the fourth one.

Interviewer: Please describe to me your experience of communication with medical personnel before you went into theatre?

Participant: It was quite well, they explained to me what I went through before they conducted a c/section on me. I was told what I would experience, I was told the pain would be there for about two weeks. They educated me on a number of things and how …I just had to say this is my way. On the communication part they did a good job even the time they were carrying me they encouraged me, they told me to have hope and that I would be fine. Before I went to theatre I was told not to eat. They chatted with me through the whole process until it was finished.

Interviewer: Do you think the information you were given was enough?

Participant: It was very enough because ah ok they told me if you want you can go through the normal delivery but you will not survive it. So after they explained to me… they gave me everything that I needed to make a final decision and afterwards they told me if you want after caesarean section there are those that go for BTL meaning permanent closure, there are those that go through where they do those that come with family planning. So they really helped me in making a good decision.

Interviewer: What aspects of information would you have loved to receive regarding communication?

Participant: they gave me all the information I needed. On my part there isn't anything that I lacked. They are professionals, they did their job.

Interviewer: Who provided information on the need to have emergency caesarean section?

Participant: It was the doctor because when I came I just came for a normal checkup and I was told it is important that I go through the c/section if the normal one doesn't go through. So it was not planned that this day I would have an operation. So he gave me everything that I needed.

Interviewer: Were you due?

Participant: Almost.

Interviewer: Please describe the process of consenting to emergency caesarean section?

Participant: There is usually a consent form that you have to sign. First they will educate you on what you will expect, how everything will be done, then they will give you the consent form where you need to agree then after signing they will start the process by preparing you for an operation. Then after the operation they will still take care of you.

Interviewer: What was on the consent form?

Participant: you have to agree that you agree for them to conduct a c/section on you. That is what is there. What is on the consent form is just the basics, the name, and your age, where you reside and finally you have to sign. You even put the date, the actual date they are operating you on. That's all.

Interviewer: What was your experience of communication with medical personnel after emergency caesarean section?

Participant: they were friendly, and whatever information I needed which I wasn't clear about they gave me the information. They told me to lie down for eight hours, after eight hours that's when you start eating, you start with the liquids.

Interviewer: Thank you so much for your time.

Participant: You are welcome.

PARTICIPANT NUMBER 11

Age: [late 20s]

Religion: Christian

Marital status: Married

Highest level of Education: Grade twelve

Occupation: working in a [removed] company

Interviewer: How many children do you have?

Participant: Two

Interviewer: Was this your first emergency caesarean section?

Participant: No, this was my second.

Interviewer: What was the cause for the emergency caesarean sections?

Participant: The first one the baby was too big 4.9kg. Then this one I was told by the doctor that the baby was too big again was told she was going to be more 3.5kg. It was 39 weeks I was going to 40 weeks. Then I was having some pain on some part of my stomach so they just decided I should have emergency caesarean section.

Interviewer: what was your reaction when you were told you were going to have emergency caesarean section for the second time?

Participant: I had to accept it because I wanted both my baby and I to be safe. So it was okay with me.

Interviewer: Please describe to me the communication with medical personnel before you were taken into theatre?

Participant: the communication was just okay although you know how these people are sometimes you find someone who is able to explain things to you so that you understand but some become so rude to you instead of explaining for you to understand.

Interviewer: Were you able to understand what they were explaining to you?

Participant: yes after sometime I came to understand what they were explaining although I differed with a person from theatre but later we came to understand each other.

Interviewer: what information were you given before you were taken into theatre, what did they tell you before they took into theatre?

Participant: they just told me that I was going for an operation.

Interviewer: What else?

Participant: nothing.

Interviewer: so they just told you are going to theatre and took you there?

Participant: they explained to me.

Interviewer: what did they explain to you?

Participant: they explained the reason I was going to theatre because part of my stomach was paining looking at I was 39 weeks and my first pregnancy was delivered through caesarean section.

Interviewer: Please tell me the process of consenting to emergency caesarean section?

Participant: I was given a form to sign.

Interviewer: what was written on the consent form?

Participant: they just told me to sign it and put my address. I didn’t go through it because I have signed that form when I had my first emergency caesarean section. Even last time they told me to put my signature.

Interviewer: Did the medical personnel read the consent form to you?

Participant: No, they didn’t they just said I should sign.

Interviewer: what factors did you consider before signing the form?

Participant: to save my baby and my life.

Interviewer: what advice did they give you after you came back from theatre?

Participant: I was explained to how to clean my wound. They told me to clean the wound three times with lifebuoy soap in a day. They also told me to lie down for 8 hours but I had to wake up to take care of my baby who was left unattended to. She was crying. I woke up when it was four hours and I am having a terrible headache because of not following what they told me.

Interviewer: What was the reason for telling you to clean the wound three times in a day?

Participant: So that it can heal fast. They also told me not to eat solids.

Interviewer: Why was that?

Participant: I don’t know, they didn’t explain.

Interviewer: Earlier you mentioned that you did not read the consent form, when do you think is the best time to educate the women about it?

Participant: I think women should be educated on the consent form before labour starts, like before the pain starts. It is better they start explaining before the woman is in pain because we just sign that form for the sake of being operated on without knowing the reason why we are signing it. That is the reason why I had a misunderstanding with one of the staff in theatre because at some point I didn’t have the courage to go to theatre. She asked me why I was in theatre and I said I don’t know then she said we are going to send you back because you can’t come to theatre without knowing why you are here. She called the nurse, you brought the person that doesn’t know why she is here. So that is how the nurse explained to her the reason she had taken me to theatre and that I should have caesarean section. My advice is it is better they explain the reason why someone is going to theatre so that they can have an idea why they are going to theatre because us we have never read about these things. They need to make us understand the reason we are going there. So like to me I was even not given a chance to deliver normally, I had wanted to try delivering normally but since I was having part of my stomach paining and it was 39 weeks I couldn’t understand what the doctor said. I would love where I have somebody to explain and make me understand so that even when I go home I am able to explain to the people about what happened and the reason why I went through caesarean section.

Interviewer: What advice have you been given on future pregnancies?

Participant: that one they have not told me anything.

Interviewer: Thank you so much for your time.

Participant: You are welcome.

PARTICIPANT NUMBER 12

Age: [late 30s]

Marital status: Married

Highest level of Education: grade 12

Religion: Christian

Occupation: Firefighter

Interviewer: How did the operation go?

Participant: the operation was just ok according to the reception I received everything went well.

Interviewer: How many children do you have?

Participant: four

Interviewer: Was this your first experience of emergency caesarean section?

Participant: this is my second.

Interviewer: What was the cause for the second emergency caesarean section?

Participant: the baby was breech and I was told it was big. Then the first emergency caesarean section I had twins. So I think that was the problem for the previous emergency caesarean section.

Interviewer: What was your reaction when you were told you were going to give birth through emergency caesarean section again**?**

Participant: my reaction ah I wanted help so it was just fine for me.

Interviewer: Were you afraid to have the operation?

Participant: No, I wasn't scared because I have gone through caesarean section before so I can even advice other women not to get scared when you go for operation they help.

Interviewer: the first time you had emergency caesarean section, were you nervous?

Participant: first time yes but when I went there I got experience and I know that they were helping me.

Interviewer: What information do you think calmed you down?

Participant: what calmed me down was the fact that they told me I would remain awake during the procedure unlike in the past where they used to put the entire body to sleep.

Interviewer: what did medical personnel tell you before you were taken for an operation?

Participant: they sensitized me so that I understand before going to theatre.

Interviewer: So what did they tell you exactly?

Participant: They told me not to be scared and that they were going to help me. They also told me that they needed to operate on me so that the baby does not die in the womb. So in short they told me that I needed to have caesarean section so that the baby and I can be alive. So I understood that they were helping me.

Interviewer: Please explain to me the process of consenting for emergency caesarean section?

Participant: there is a form they give you to sign before you are taken into theatre in case of anything. So they make you sign a form in case you died in the operation room at least there is evidence that you had agreed to be operated upon.

Interviewer: What was written on the consent form?

Participant: Mmmm I think I need go through it properly.

Interviewer: Didn't you read it before you appended your signature?

Participant: I tried to read it but I was not settled since I was a patient.

Interviewer: Did someone read the form to you?

Participant: No, I was just told to sign the form so that my baby could be saved and my life too.

Interviewer: Who gave you the consent form?

Participant: It was the doctor.

Interviewer: what made you to sign the consent form?

Participant: what made me sign was because the communication was good because they told me it was necessary to do the operation to save my baby and life. If I had refused to sign the form I wasn't going to be operated on and I don't know what would have happened to me and my baby.

Interviewer: So you signed because you wanted to be operated on?

Participant: I signed the consent form because I needed help.

Interviewer: What advice were you given after the operation?

Participant: I was advised to take care of the wound properly.

Interviewer: What else?

Participant: long silence ….. I don't want to say…. Another long silence …ok they said I should rest and not eat solids.

Interviewer: What reason did they give for not eating solids?

Participant: because the intestines and the stomach where you had the operation can burst because of the hard food.

Interviewer: Is that what the medical personnel said?

Participant: ha ha ha no I just made an assumption that this could be the reason why they advise us not to eat solids. They didn't tell me the consequences or reasons why I should not eat solids.

Interviewer: Earlier, you mentioned that you didn't have time to read the consent form, what would be the best time to educate the women about it?

Participant: I think the best time to explain the consent form would be during antenatal classes in case pregnant women end up having an emergency caesarean section. So they need to educate us in advance before a woman is due because when you have pain you can't manage to go through the paper. So we sign the consent form without any information. So it is important that they should find time to teach us about the consent form in our various hospitals we go to for antenatal classes.

Interviewer: As we conclude this interview, what would be your concluding remarks on the aspects of communication with medical personnel?

Participant: ok what I can say is that medical personnel help us a lot but they need to be lenient to patients because a patient is usually in pain. Sometimes they are rude but they need to understand the patient and talk to them nicely so that they understand and that way there will be proper

communication. In my case I was in pain so I didn’t want to ask questions for fear of upsetting them because I was the one who was in need.

Interviewer: When you say they should be lenient, what do you mean?

Participant: I meant they should treat patients in a respectful manner and not be rude. They should understand each patient and talk to them in a polite way because a patient is in pain and their attitude can worsen the condition. You know each person is different and we react differently.

Interviewer: Thank you so much for your time.

Participant: You are welcome.

PARTICIPANT NUMBER 13

Age: [mid 30s]

Highest level of Education: grade seven

Occupation: Business lady

Religion: Christian

Marital status: Married

Interviewer: How many children do you have?

Participant: I have six children.

Interviewer: Was this your first experience of the emergency caesarean section?

Participant: No, this is the third one.

Interviewer: what was your reaction when you were told that you would give birth via caesarean section again?

Participant: On this one, they wanted to tie my tubes (tubal ligation) but I told them that I needed to consult my husband first. I can’t sign for the surgical procedure without consulting my husband. So we will only discuss this issue with medical personnel when my husband brings me for review.

Interviewer: What caused the third emergency caesarean section?

Participant: Medical personnel told me that my vagina would get torn if I gave birth normally since I have had two operations.

Interviewer: What caused the first operation?

Participant: the first operation the baby was breech, the second one they just conducted the operation too early because the baby was already in a position to be born so they started shaking me so that the baby could pass where there was an incision.

Interviewer: Please describe the communication with medical personnel before they took you for the operation.

Participant: when I arrived at the hospital I requested medical personnel if they could give me a chance to deliver vaginally but they said no you will put us into trouble because you have already had two previous operations. So you have to be taken to theatre without delay. When I got to theatre they said I have too many children they should just tie my tubes because if you continue having children you will die and your children will be orphans.

Interviewer: So how did you take this?

Participant: I couldn’t agree to have my tubes tied because my husband wasn’t there. Its only when my husband agrees that we can allow the nurses to do it.

Interviewer: So before they took you into theatre, what information were you given about the emergency caesarean section?

Participant: No, they told me that I just needed my tubes tied.

Interviewer: I am referring to the operation for removing the baby. What did the medical personnel tell you before they took you into theatre?

Participant: they just said I can’t deliver normally even if they gave me a chance to do so.

Interviewer: Were you nervous about the operation?

Participant: No, because I have experience.

Interviewer: Were you given any form to sign before the operation?

Participant: Yes, before I was taken into theatre I was told to sign a form indicating that I am going into theatre.

Interviewer: So what was written on the form?

Participant: what was written on the form is that there was danger, there was danger that I needed to go to theatre they had even indicated it in red pen.

Interviewer: What else was written on it?

Participant: Mmm I didn’t see other things because I was in pain when they brought me to [hospital removed] but last month they had given me an appointment that I needed to be here on the 12th of April so that on the 15th they take me to theatre and they had emphasized that I should not wait until I went into labour but I went into labour before the 12th.

Interviewer: Did the medical personnel read out the form to you?

Participant: No, they just told me to sign.

Interviewer: What factors did you consider before signing the consent form?

Participant: I signed because they told me to sign it and that senior doctors would not allow me to go into theatre without signing.

Interviewer: Who provided the consent form to you?

Participant: There was a nurse and a male doctor.

Interviewer: Please describe the communication with medical personnel after you came back from theatre?

Participant: there is nothing else they told me after I came back from theatre. While I was in theatre they just said I need to have my tubes tied.

Interviewer: So even here in the ward they haven’t told you anything?

Participant: They have explained how I should take care of the wound, clean it with lifebuoy so that it heals fast.

Interviewer: What else?

Participant: Nothing.

Interviewer: what advice did they give you on laying down after theatre?

Participant: No they didn’t say anything about that.

Interviewer: I noticed that you were looking forward to this interview when I was taking you through the consent form but after I asked you to sign you hesitated, was that your experience with the consent form they give you before you went to theatre?

Participant: yes it is scary because it is like something bad is going to happen to you.

Interviewer: Did you have all your emergency caesarean sections here at [hospital named]?

Participant: Yes.

Interviewer: What differences did you notice in terms of communication?

Participant: the only difference is that this one they have told me to consider having my tubes tied but the first two emergency caesarean section they never said anything about tying the tubes. So for them to tell me now I think they know what they are doing.

Interviewer: Thank you for your time.

Participant: Thank you. I am grateful too.

PARTICIPANT MUMBER 14

Age: [early 30s]

Religion: Christian

Occupation: Business Lady

Highest level of Education: Grade 12

Interviewer: How did the operation go?

Participant: it was just ok. It was fine.

Interviewer: When was it done?

Participant: On 3rd April.

Interviewer: How many children do you have?

Participant: Two

Interviewer: Was this your first experience of emergency caesarean section?

Participant: No, this was the second.

Interviewer: What caused the first and second operations?

Participant: the first operation the baby was breech and then the second one ah the road is not ok.

Interviewer: When you say the road is not ok what do you mean?

Participant: Ok I should say the placenta was not fine for the baby.

Interviewer: what was your reaction when you were told that you were going to give birth through caesarean section for the second time?

Participant: nothing I just wanted to be helped.

Interviewer: How did that make you feel, were you nervous, were you scared?

Participant: I was not scared because I really needed help.

Interviewer: Describe the communication with medical personnel before you were taken to theatre?

Participant: After I went to the clinic they put me on observation, which is the second born baby. After some five hours they said time has passed and that I needed to go through an operation again and that is how I came here.

Interviewer: Which health facility was that?

Participant: [hospital name removed]

Interviewer: So when they brought you here, what information were you given, what did they say to you?

Participant: Ah they just told me that I needed to go straight to the theatre and I accepted.

Interviewer: What else did they tell you?

Participant: nothing.

Interviewer: So they just took you into theatre?

Participant: Yes.

Interviewer: What was going through your mind when they were telling you that you have to go to theatre?

Participant: nothing I just wanted help.

Interviewer: please describe to me the process of consenting to emergency caesarean section?

Participant: I accepted and I was the one who signed the form.

Interviewer: What was on the form that you signed?

Participant: they told me to sign. The other things I don't remember. But I finished signing the form and even indicated the time.

Interviewer: What else was written on the form?

Participant: Hmmm I was in pain so I was just signing where there was a provision for signing.

Interviewer: Did they read the form to you?

Participant: Yes, she did.

Interviewer: So what did she read out to you?

Participant: Ah I can't remember.

Interviewer: What factors did you consider before you signed the form?

Participant: I signed it because I am the one who needed help. I also did it for the safety of the baby and mine.

Interviewer: After you came back from theatre what information did medical personnel give you?

Participant: ah they didn't give me any information.

Interviewer: They didn't tell you anything?

Participant: Nothing. From theatre they took me to the side ward then they brought the baby there. Then in the morning they started giving medicine, that's all.

Interviewer: they didn't tell you how you should take care of yourself?

Participant: Actually they did. I forgot. They told me to lay down for eight hours and not eat any solids. After eight hours I started drinking pure juice.

Interviewer: What was the reason for telling you not to eat any solids?

Participant: Because the operation wound was still fresh and if I ate something I would get into problems.

Interviewer: is that what they said?

Participant: not really ha ha ha I just figured it out myself.

Interviewer: what was the reason they told you to lay down for eight hours after the operation?

Participant: I don't know the reason and I didn't ask them.

Interviewer: Apart from laying down for eight hours and not eating solids, what else did they tell you?

Participant: They also told me how to clean the wound and how to take care of myself as well.

Interviewer: Earlier you told me that you were in a lot of pain and could not read the consent form, when do you think is the best time to educate women who undergo emergency caesarean section?

Participant: I think during antenatal would be the best time for nurses to explain the consent form than when you are in labour they give a form to read, it is impossible. So you need to read it before so that when you are going to theatre you know everything.

Interviewer: Did you think it was ok for you to sign something you don't understand?

Participant: Kaili I was in pain, I didn't know anything when I went in theatre but I just signed because I really needed help. In fact some of the questions I was asking the doctor what I should write. I was just asking him. I didn't even read the form yeah.

Interviewer: What did you write on it?

Participant: my name, signature, the time I came and the date.

Interviewer: Ok, but how was the communication between you and the medical personnel?

Participant: it was ok because she saw I was in pain and she understood me. But I want to ask you a question.

Interviewer: Please go ahead?

Participant: How many caesarean sections can a woman have?

Interviewer: Thank you for the question but the best person to ask would be the nurse or doctor so you can ask them when they come. My specialty is in communication. Any other question?

Participant: No I just wanted to also learn from you since you are learning from me.

Interviewer: Thank you for your time.

Participant: You welcome.

PARTCIPANT NUMBER 15

Age: [early 40s]
Religion: Christian
Marital status: Married
Highest level of Education: degree in development studies
Occupation: Teacher
Interviewer: How was the operation?
Participant: It was successful.
Interviewer: How many children do you have?
Participant: Ha ha ha now they are four with this one. I had three.
Interviewer: nice, so is this a boy/girl?
Participant: ha ha ha it is a girl. I have two girls, two boys. The first was a girl, then the second and third are boys then the fourth one is a girl ha ha ha.
Interviewer: Wow that's great. Was this your first experience of emergency caesarean section?

Participant: yes, it was the first one.

Interviewer: What was your reaction when you were told that you were going to deliver through emergency caesarean section?

Participant: Well yeah I have always had phobia but because I was too excited to see my baby I just calmed myself down that my baby has to be saved.

Interviewer: You said you initially had phobia what information do you think would have calmed you down or relieve that phoebia?

Participant: just the excitement to hold my baby. This baby was not planned for. My last born is 13 years old so this one happened without me knowing I was expecting. I only got to know when I was 16 weeks. So with time I got too excited and I was really looking forward to having this baby. So when I was told that to save my baby the only way is caesarean section I had no option but to go for it because I really wanted to hold this baby.

Interviewer: How would you describe the communication with medical personnel before you were taken into theatre?

Participant: I think it was ok, it was good, it was excellent because when I arrived first it was normal labour, the nurse was able to communicate that it had just started and they were checking on me. When it was four hours later and the progress was not good so the doctor said no it has taken too long from the time the water broke so we need to save the baby. Four hours later it was still at 3cm and I was bleeding a lot. So the doctor felt that the baby was at risk and he needed to make a decision to save the baby. So they informed me that we need to take you for Caesar for the sake of saving the baby.

Interviewer: Apart from that what other information did they give you?

Participant: I don't think there was any other information. I was just informed about the emergency caesarean section and was given the consent form to sign because I was alone my husband was at home so they made me sign for myself to give consent for them to carry out the operation.

Interviewer: Please take me through the process of giving consent for emergency caesarean section?

Participant: I was given the form which had details that I needed to fill in where I was declaring that I was giving consent for them to carry out the operation.

Interviewer: What else was written on the form?

Participant: I think it just had the provision of the next of kin, my details and then I was to sign yes**.**

Interviewer: Who made you sign the consent form?

Participant: can I remember?

Interviewer: was it a nurse or a doctor?

Participant: I think it was a nurse, midwife if I am not mistaken. It was a midwife. In fact the doctor told me I was going to have caesarean section then the nurses who were preparing me for theatre are the ones who gave me the consent form.

Interviewer: what factors did you consider before signing the consent form?

Participant: hmm at that point I didn't consider anything. All I wanted was to save the baby that is all and somehow I felt it was a relief because I needed to do BTL. So this was the opportunity for me killing two birds with one stone.

Interviewer: Do you think the information you were given before going into theatre was enough?

Participant: mmm I would say yes I would say no because it was an emergency they didn't really like take me through, maybe counsel me I wasn't really like counselled because maybe under normal circumstances they would have taken me through the process this is what is going to happen and this is what you should expect and maybe we expect negativity in this way and positivity in this way. There was no time for that I was just prepared and taken. So I just had excitement to see the baby but I didn't have information on the procedure and what to expect or what I will go through whilst in the theatre.

Interviewer: What information would you have loved to receive before you went into theatre?

Participant: Kaili being the first caesarean section I would have loved if they counselled me to say this is what is going to happen to you and the aftermath is like this, you will be feeling like this you know so going in there I was just excited about the baby but coming out of the process was too much for me because the whole day I was laying in one position. I was told not to move, don't turn, the wound itself was so painful. I felt inshi if I had known I wouldn't have gone for it. So I wish I was counselled and taken through the process for me to know what to expect because hehehe I felt like I was going to die.

Interviewer: I am sorry about that, what were the reasons they told you to lay down for a long time without turning?

Participant: maybe it's due to side effects because if you move or turn you are likely to have migraine headaches which are bad because I am hypertensive so they just said you need to be still in one position for eight hours when I was brought here (in the ward). I wasn't even able to hold the baby or do anything.

Interviewer: What other advice have they given you since you came to the ward?

Participant: Ah there was nothing apart from just from laying in one position for eight hours. I was laying without turning because they said the side effects are very bad but it felt like torture ha ha ha there I was excited to hold my baby but until later in the day. I remember the nurses from here told me that after that eight hours I can't eat I can only take fluids.

Interviewer: What was their explanation why you shouldn't eat solids?

Participant: mmm I think there was no reason given but I just figured it out that because it was an operation I just needed to take fluids and not anything that would expand the stomach so much, it would affect the wound so maybe the whole idea was about keeping less food in the stomach so that the healing process can start without being disturbed and maybe because I was needed to lay down for eight hours the bowel movements were going to make me go to the toilet.

Interviewer: Apart from laying down for eight hours, what other advice were you given?

Participant: they have also given me hints on how to take care of the wound being the first caesarean that I have had. So just this morning they were showing me how to clean the wound so that it is not septic. That is all.

Interviewer: Did they say anything about how to take care of the baby?

Participant: Ha ha ha no they didn't I suppose they assume I know how to take care of the baby since I am a mother.

Interviewer: What recommendations would you make regarding communication with medical personnel before and after emergency caesarean section?

Participant: Ah that is a difficult question because it depends on the circumstances like if it is really an emergency where they have to save a child I wouldn't blame them for not taking me through the procedure and what to expect but if it is planned caesarean section I think someone can be prepared.

Interviewer: When do you think is the best time to educate the women on the procedure and what to expect and ensure that communication is there to allay the anxiety?

Participant: being an emergency no one prepares an emergency but maybe the time the nurse was preparing me to go into theatre maybe she should have given me a hint about one or more things that are important so that my mind is ready for it.

Interviewer: As we conclude the interview is there anything of importance that you would like to talk about regarding the communication between medical personnel and women who undergo emergency caesarean section?

Participant: it is important that medical personnel counsel the patients, bring out the expectations, bring out the side effects, the advantages and disadvantages so that even when you go there (theatre), you have an informed decision and your mind is ready for it so that you are not surprised because like when I came out I was literally shivering, I wasn't feeling cold but I was having this ah involuntary kind of shivering and then I was subjected to that eight hours, it felt like torture. But if they had told me that after the operation this is how you are going to feel my mind was going to be ready for it because the way I went through it ha ha ha I wouldn't advice any person under normal circumstances to go for it you know so that is how it is. So communication is very vital and medical personnel shouldn't take it lightly especially when we have women that are not educated or that are not iliterate so to say they don't understand much of the things. So it shouldn't be left to the patient to figure out, it is important that they communicate and not just saying we have to save the baby.

Interviewer: Thank you so much for your time.

Participant: You are welcome and hope your study brings in change in the care for women who undergo emergency caesarean sections. It's not easy madam.

PARTICIPANT NUMBER 16

Age: [late 30s]

Highest level of Education: Diploma in Institute for Management Information System

Occupation: Business lady

Marital status: Married

Interviewer: Was this your first experience of emergency caesarean section?

Participant: yes it was**.**

Interviewer: How was it?

Participant: mmmm it was mmmmm, ok I have normal births like the previous pregnancies were normal and I had a bit of experiences on these pregnancies where I would have developed high blood pressure towards the end of the pregnancy and I have two kids and I had two that didn’t make it due to high blood pressure all that. So when I got pregnant with this one I think I told myself that I will do whatever it takes to keep this pregnancy. So I think I started even preparing my mind that just in case they suggest caesarean because of these issues I told myself I think I will do it but I just had to do some. I told myself that this one so whatever it will take either caesarean or normal I just have to do it. So when I came here everything was ok but I think this is the furthest I have gone with a pregnancy and when I came there were like no since you have had problems with previous pregnancies I don’t think we can risk you going further like going upto your due date and after the history they read they decided to say as long as you are over 34 weeks we are going to deliver your baby because this might induce some other problems that you have had. So I told the doctor that I think I am ready for that. So everything went according to plan and they told me since you are almost there we have to induce you on Friday. I came here on a Wednesday. They said we have to induce you on Friday because we don’t want you to have the experiences you have had and I was ready for that so I told them ok. So on Friday they actually induced me and it was in the afternoons. As the day was progressing nothing was happening I don’t know if it didn’t work or something. Then they decided to give me some more medication but still it couldn’t work then they just decided we can’t risk anymore so the only option is that we just take you to theatre and just remove the baby. Before they could even finish I told them I was ready for

caesarean just like I said earlier I think I prepared my mind to say I will do whatever it takes to have this baby. That is what happened.

Interviewer: I am so sorry for your loss. What was your reaction when it really turned out you were going to have caesarean section?

Participant: I think I was relieved somehow because I know the previous ones I was induced and I went through normal labour, the first one I went through normal labour thinking the baby was fine and I delivered but the baby didn't cry, the second one the baby was fine and it cried but I wasn't based in [removed] my husband was transferred to [removed] but there the health facilities were not ok. They actually didn't have incubators, no oxygen so the baby only stayed for about six hours and couldn't make it on her on. She needed help with breathing. So when I discovered I was pregnant I decided to come back to [removed] at least I am going back to [removed] because I know at least [removed] should have everything, the facilities that are needed. So immediately I got pregnant we planned that I was going back to [removed] and that is what we did. So I think I was relieved.

Interviewer: What information did you think made you feel relieved?

Participant: Because of the experiences I have had, I just did research on my own like I would read this I would read that and what I found out was when you are having a normal natural birth somehow the baby develop stress like they are distressed. So with me I considered caesarean to be faster than the normal birth where you go through the process dilate all that. With caesarean I knew that the baby would come out faster and she or he would be attended to.

Interviewer: How would you describe communication with medical personnel before you were taken to theatre?

Participant: it was ok they actually counselled me, they actually had time to talk to me they didn't impose it on me so they wanted to seek permission whether to go ahead with the caesarean section. So I think it was fine because the doctor induced me around 11 hours and only decided upon taking me to theatre around 02 hours. So what they actually explained to me was we can't wait any longer because according to what they expected at least I should I have dilated like I was about to give birth so when they said they couldn't let me go back to dilate more until you are ready to give birth

so they explained to me about that. They suggested to say that maybe we go with the caesarean thing.

Interviewer: Were you anxious or nervous when you were being taken to theatre?

Participant: I wasn't nervous but I was anxious and it's like I was told around 02 hours that I needed to go into theatre. I am told there was an emergency that came like I was left there for some time waiting when all I wanted was to go into theatre.

Interviewer: what information do you think would have calmed down?

Participant: ah I think when I was told to say you are going to theatre I knew it was an emergency and I expected them to take me right there and then but I remained in the ward up until 7 in the morning, I went for my caesarean at 7hrs.

Interviewer: so what information would have allayed anxiety?

Participant: I think the thing I didn't do was to inquire if it was really really an emergency that they needed to operate on me right there and then. I think that is what I didn't do. I thought it was an emergency and all I wanted was just to go. So after they had left that is when it occurred to me after I saw they took a bit of time so I started wondering was it really an emergency so when the doctor came that is when I asked him is there a queue I thought I was supposed to go immediately you told me then he said I am very sorry yes you were supposed to go but we received an emergency that really needed attention or something. So I said ok.

Interviewer: Did you think the delay to take you into theatre should have been communicated to you?

Participant: probably, like immediately they noticed that they should have at least come back to me and informed me because even the preparations were done fast I was ready immediately I was told I was going for caesarean.

Interviewer: Was the information given to you before you went to theatre enough?

Participant: yes I think it was enough for me.

Interviewer: what other information would you have loved to receive before you went into theatre?

Participant: I think they should have told me how urgent it was that I should deliver there and then.

Interviewer: please describe to me the process of giving consent for emergency caesarean section?

Participant: after they counseled me they actually gave me a consent form to sign and I know usually that is signed probably by somebody else but nobody was there and I was alone, my husband wasn't there, my mum wasn't there so I had to sign it. They actually just explained to me that they needed more like permission from me to say I have allowed them to go ahead with caesarean section.

Interviewer: What was written on the consent form?

Participant: just accepting to say that I have allowed them whatever something like that hmmm like agreeing to them to go ahead with the operation.

Interviewer: who gave you the consent form?

Participant: There was a doctor and nurse. The nurse was the one who gave me the paper and the doctor did the explaining and I was able to fill in.

Interviewer: what factors did you consider before signing the consent form?

Participant: just like I explained earlier I just wanted this to be done with. I don't even know if I read everything but I just put my signature there because all I wanted was for the baby to be safe ha ha ha.

Interviewer: please describe the communication with medical personnel after the caesarean section.

Participant: ah after I came back from the theatre they were actually helpful. They answered all the questions I had because I didn't know what to expect I know people say it's painful but I had to go through it for me to know that it is actually painful. I was put in a certain room for observation and I was wondering why so I had to ask why I was being kept there instead of being taken to the ward. So they just said no this is the procedure we can't just take you to the ward because you have just come out of theatre. So they needed to observe me that I am ok. So at least they answered all my questions. At some point I was thinking they must be saying isn't it just the medicine

working the way she has been asking questions ha ha ha I really wanted to know because my stomach was paining and I wanted to know if they had given me pain killer. So I was wondering why my stomach was paining and why I was feeling very cold. But when I asked they answered all my questions – they were so patient with me. They told me no no don't worry we are going to give pain killers the nurses will when you go back to the ward.

Interviewer: So they actually didn't explain to you the aftermath of Caesarean section?

Participant: No, they didn't explain all that. It was only after I had asked.

Interviewer: what advice did they give you after theatre?

Participant: when I was brought here I was told to sleep up to eight hours and don't move because of the medication they gave me. They said I could only drink something after eight hours.

Interviewer: What was the reasons they gave for this advice?

Participant: they wanted me to start with fluids that are lighter so that the wound heals fast and they also said I should make sure I be strong that I should be walking for me not to have problems later for as long as it was ok and I was able to. They also told me not to lift heavy stuff until I had healed.

Interviewer: In your opinion, what other information would you have loved to receive before or after surgery?

Participant: I think on my part it was just the pain I felt I know people usually say it is painful but I wasn't told exactly what happens because I didn't understand why I was feeling very cold but later on after I asked they told me no it is the medication but it will clear afterwards and with me I felt hmm this is not normal am I going to be ok because when I came told mum to put blankets I told her to close the windows but nothing helped not until after everything. I think if someone had told me what to expect after caesarean section that would have helped.

Interviewer: did you experience some of the things they told you?

Participant: yes.

Interviewer: like?

Participant: like feeling numb they had told me that I would feel numb and be able to watch the procedure as it happened.

Interviewer: what recommendations would you make in terms of communication with medical staff for women who undergo emergency caesarean section?

Participant: hmmm probably with me I think I was ready like I told myself that I go for whatever decision they make but there are people who don't even think about that like in my previous ones I never thought I would go through caesarean section in my life so the thing is I think they should also make it a habit to explain to the mothers whether they decide to deliver normally or via caesarean section, they should be explaining both sides just in case because these are emergencies it's not like we plan them. So I think it would be better if they like maybe when you go for antenatal they explain both angles where they explain what goes on and tell you that even if your pregnancy is normal there are possibilities of going through emergency caesarean section because there people who get so confused and you know they have told you it's an emergency so you don't even have time to think about this process nicely. So I think if it would be nice if they were informing people in advance that this can also happen and that can also happen and these are the advantages and these are the disadvantages.

Interviewer: When they presented you with the consent form, did they explain the risks associated with surgical procedure?

Participant: No, they didn't explain any risks.

Interviewer: As we conclude this interview is there anything you would like to talk about regarding the aspect of communication?

Participant: I think just talking on behalf of other women who undergo emergency caesarean section, we need more information on things we go through as women like giving birth ha ha ha if they were to be explaining to women this would be helping a lot.

Interviewer: What do you think are the consequences of explaining there and then?

Participant: when they explain to you in advance you have more time to prepare and decide the more time to ask questions because if they tell you there and then I don't think sometimes definitely you have questions after like you start thinking about it why didn't I ask that why didn't I find out

about that but if you are told way in advance you have enough time to think, process and do research on your own and find out what you don't understand from them but there and then you don't have time to ask questions because it is an emergency so you might even miss out important questions.

Interviewer: your conclusion remarks?

Participant: nothing much but I hope from your research will help a lot of women I know not everything is taught there are things that we find out on our own reading or through someone asking questions and all that. So I pray this can help someone one day.

PARTICIPANT NUMBER 17

Age: [early 30s]

Highest level of Education: Grade nine

Literacy: able to read and write Chinyanja

Religion: Christian

Marital status: Married

Occupation: Business lady

Interviewer: was this your first emergency caesarean section?

Participant: no, this is the third operation.

Interviewer: how many children do you have?

Participant: I have three children and all of them were delivered through emergency caesarean section.

Interviewer: What was the cause of the current caesarean section?

Participant: there was a problem with the placenta.

Interviewer: What about the first two caesarean section?

Participant: the first born it was due to placenta problems again. Then the second child I just started bleeding without feeling anything so the doctors were afraid that the baby could choke with blood and had to do the operation.

Interviewer: Were you referred here from the local clinic?

Participant: I started bleeding around 04 hours then I noticed that it was becoming worse and I decided to come to [hospital name removed] since my card was written in red. When I got here they examined me and even sent me for the scan and was later taken to theatre.

Interviewer: Describe communication with medical personnel before you were taken to theatre?

Participant: ah they were just encouraging me because I was in big trouble. A lot of people are shocked that I am alive.

Interviewer: Were you given any form to sign?

Participant: Yes

Interviewer: What was written on it?

Participant: To sign if you want to be transfused and if you don't want to be transfused you don't select that part. But they don't force you to sign the form.

Interviewer: What else was written on the form?

Participant: There was a provision for blood transfusion and the surgical procedure but if you refuse they can't operate on you.

Interviewer: who provided you with the consent form?

Participant: it was the doctor.

Interviewer: Did the doctor read it to you before you signed it?

Participant: no they just give you the form then you have to decide either to sign it or not. In my case I only signed where there was a provision for the operation and left out the part for blood transfusion since I am a member of the Jehova's Witness and our faith is against blood transfusion. But when I fainted they transfused me when they realized that I was going to lose the baby. They transfused me on account that I didn't have a Johova's Witness card indicating that I am a member

although I had not signed. They later explained to me this morning that they had to transfuse me to save my life and that of the baby.

Interviewer: What was your reaction when they explained to you?

Participant: I can't blame them because they saved my life and the baby's. I can't even be angry because it now in the past.

Interviewer: what would have happened if you had a card from the Kingdom Hall?

Participant: they wouldn't have transfused me and I would have just died.

Interviewer: what information did medical personnel give you before going to theatre?

Participant: ah nothing they only told me that they would be taking me to theatre and the reason for doing so. They said the placenta had blocked the birth canal and that any mistake the baby would die.

Interviewer: After theatre what did they tell you?

Participant: they said it is good to have faith but that on issues to do with lives I should make decisions that save life. You should have signed all parts on the form. We had to look for blood and ask people to donate blood so that we could transfuse you at our discretion. But still more your HB is still low so you need more blood transfusions.

Interviewer: What else did they tell?

Participant: they only gave advice on the kind of food I should eat to have enough blood.

Interviewer: What about things like laying down for eight hours, not eating solids and how to take care of the wound?

Participant: No, they didn't tell me any of that maybe because this is my third operation so they think that I know. I am sure if they will have something to tell me they will in the evening.

Interviewer: what information or advice would you want medical personnel to give you?

Participant: nothing I am just happy that they saved my baby. They did a great job because I was really in big trouble. They saved my life and baby's.

Interviewer: I want you to focus on the aspect of communication.

Participant: I didn't notice any problem. I have never experienced any problems with nurses. They always take good care of me. I now remember they told me to lay down for eight hours and not to eat solids.

Interviewer: What was the reason they gave you for not eating solids after the operation?

Participant: they didn't tell me and I have never asked them but I think the reason is to prevent the synthetic thread used in suturing from breaking.

Interviewer: What are your concluding remarks?

Participant: nothing. I end here.

Interviewer: Thank you for your time.

Participant: Zikomo [Thank you].

PARTCIPANT NUMBER 18

Age: [early 30s]

Religion: Christian

Highest level of Education: Grade nine

Occupation: House wife

Marital status: Married

Interviewer: How many children do you have?

Participant: Six.

Interviewer: Was this your first experience of emergency caesarean section?

Participant: yes it was the first. All the children I have had normal deliveries except this one. I first went to [clinic named removed] where they told me that the baby was breech. So the doctors told me that they can't manage to conduct caesarean section and referred me to [hospital named removed]. I got here around 10hrs and they examined me and confirmed it was breech. So they were talking amongst themselves that I needed to give birth via caesarean section since the baby

was breech. The baby had also pooped inside the stomach. So they said it was a big problem and that I needed to be operated on so that the baby can survive. They said both the mother and baby could survive if we quickly do the operation and that if we delay only the mother or baby might survive. The baby was also already tired in the uterus (fetal distress) so that is how they conducted the caesarean.

Interviewer: what was your reaction when you were told that you were going for caesarean section?

Participant: I wasn’t afraid because I put everything into God’s hands. I had no choice so I said let God’s will be done.

Interviewer: Describe the communication with medical personnel before you went into theatre?

Participant: the nurse explained the problem I was experiencing because I had abdominal pain for three days and only went to the hospital on the fourth day. So I just followed whatever the nurse told me so that I can survive and be able to raise my children.

Interviewer: Do you think the information you were given was enough?

Participant: ok initially I thought they were going to put me on a drip so that I can gain some strength and I thought they were going to wait for the baby to turn like what the other nurse had suggested when they were talking. But the doctor is more senior so they nurse had to just follow what he had said.

Interviewer: were you scared of the operation?

Participant: yes I was scared at the beginning but with God on my side I eventually calmed down.

Interviewer: what information would have calmed you down?

Participant: having faith in God calms me down because he makes a way where there is no way. God has the final say. God guided me on what to follow and accept caesarean section.

Interviewer: Did they give you any form to sign?

Participant: yes, they did.

Interviewer: what was written on it?

Participant: Ah it was an emergency so I didn’t have time to read it. They just showed me where to sign and I appended my signature. I put my name, number of next of kin and my signature.

Interviewer: So what did they tell you before you signed the form?

Participant: they only told me that the doctor had recommended that I should have caesarean section and that I needed to sign the consent form. So they asked me if I accept or decline signing so I accepted and they gave me to sign. When I got into theatre the people there asked me if I wanted to have more children and I said only God gives children. So I can’t say tie my tubes because God is the one who gives children. So I told them I will continue bearing children as long as God gives me. I don’t know if they wanted to tell me that they wanted to turn the baby since it was breech but they didn’t maybe because I told them I didn’t want to have my tubes tied.

Interviewer: When you were being taken into theatre did you think they were going to turn the baby or to conduct the operation?

Participant: no they told me that I was going for caesarean section.

Interviewer: Who made you sign the consent form?

Participant: I don’t know if it was a doctor or nurse but they appeared like junior doctors and not senior doctors at mid - level

Interviewer: What advice were you given after the operation?

Participant: they told me to lay down for eight hours and after eight hours they told me to turn but because of the post-partum hemorrhage (PPH) I had it was difficult for me. But when I gained some strengthen I realized that they have really taken good care of me. I have been in the hospital for more than seven days because of low Hb. They also told me not to drink any water in the eight hours and no solids.

Interviewer: what was the reason for that?

Participant: I was told to lie down for eight hours to prevent a terrible headache so they want the anaesthesia medicine to reduce. Regarding food I think so that you are not in trouble with the wound. They also taught me how to clean the wound using cotton and a face cloth twice a day.

They have also taught me how to breast feed the baby. Then they also told me I should wait for two years before falling pregnant so that the wound can fully heal.

Interviewer: what would you have loved the medical personnel to tell you since it was your first operation?

Participant: I think they should teach us about emergency caesarean section at antenatal classes so that if we end up having the procedure we make an informed decision. It is too sudden to process everything when you are told you are going for an operation. So we need more time to understand and accept the procedure. If you learn earlier that there is unplanned operation during antenatal and that it is not always the case that you will have normal delivery even if you had a smooth pregnancy it would help a great deal because we would easily accept as we are being wheeled into theatre. I only accepted to sign the consent form because of the problem I had and doctors know what they are doing so you just follow what they are telling you. All they need to tell me is the problem I am experiencing. I couldn't ask any questions because of the way they handled me it really looked like an emergency.

Interviewer: Thank you so much for your time.

Participant: you are welcome.

PARTICIPANT NUMBER 19

Age: [early 30s]

Number of children: 7

Marital status: married

Highest level of Education: grade seven

Occupation: House wife

Religion: Christian

Interviewer: was this your first emergency caesarean section?

Participant: this is the second operation.

Interviewer: What were the causes of the first and second operations?

Participant: the first one the problem was that I had triplets so they had to operate on me. Then the second was an operation too because I conceived before the stipulated period advised by nurses and doctors. I fell pregnant again within a few months of giving birth to triplets. So the previous operation had not fully healed. The first operation of triplets was done about a year ago.

Interviewer: Describe the communication with medical personnel before you went to theatre?

Participant: It was just ok understanding you, you know those people save our lives.

Interviewer: What was your reaction when you were told you were going to have an operation again?

Participant: I accepted it because the doctors wanted to save my life. If I had refused there are a lot of things that happen.

Interviewer: What kind of things happen?

Participant: the previous operation wound had not healed so I thought my uterus would get destroyed or I might just get other complications. So it is a good thing I accepted the operation. I wasn't even scared because my first operation was successful so I knew that even this one was going to be fine.

Interviewer: what kind of information would you have loved to receive before going to theatre?

Participant: Just words of encouragement.

Interviewer: what did medical personnel tell you before they took you to theatre?

Participant: they just asked me if I had eaten something and encouraged me that everything was going to be fine because three quarters of women are scared of the operation.

Interviewer: Please describe the process of giving consent to emergency caesarean section?

Participant: there are many reasons we sign the consent form.

Interviewer: What was written on the form?

Participant: Ah I didn't pay attention.

Interviewer: So you just signed?

Participant: yes there was a provision where I should sign and I did so. You know when you are going for an operation you have to accept even if anything happens you would have already signed.

Interviewer: so who provided the form?

Participant: it was the nurse.

Interviewer: So what made you sign the form?

Participant: I signed it because I was going for an operation.

Interviewer: What advice were you given after the operation?

Participant: they told me to lay down for eight hours and that I should clean the wound for three times per day so that it heals quickly. The reason they told me to sleep for eight hours was for the medicine to work well. Headache would be prevented too. I can’t remember other things they said.

Interviewer: What other advice would you have loved to receive from medical personnel?

Participant: Just encouragement because three quarters of people think that they can die when they are operated upon they are usually scared but for me I have vast experience that is why I was not scared.

Interviewer: Thank you so much for your time.

Participant: You are welcome.

PARTICIPANT NUMBER 20

Occupation: [post removed]

Highest level of Education: Diploma

Religion: Christian

Age: [mid 20s]

Interviewer: How many children do you have?

Participant: this is my first child.

Interviewer: Since this is your first pregnancy, what was your reaction when you were told you were going to deliver via caesarean section?

Participant: ok what came to my mind was that the baby will be fine and I will be fine as well due to the complication of me having high blood pressure I was expecting such. My mind was prepared I knew that something like this would happen all I wanted was for me and the baby to be ok at the end of the day.

Interviewer: Please describe to me the communication with medical personnel before you were taken to theatre?

Participant: the communication wasn't all that good whereby I was referred from a [hospital name removed] to here as an emergency then I was told they would take me into theatre but I kept on waiting and waiting from 18 hours up to 12 hours the following day that is when I was taken into theatre whereby they knew that my case was also an emergency but they didn't treat it as such but anything can happen at any moment looking at the high blood pressure I had then my baby would have gone into distress it can affect her and also me. So when you try to talk to them they were like no we will take you we will take you until the following day at 12 hours that is when I was taken into theatre. There was no communication and the worst part is that you are made to starve you don't get to eat anything I think the maximum that you are supposed to stay without food is like six hours but I had to stay from 18hrs the previous day to 12 hours the following day without eating anything when I was previously starved at [hospital name removed] where I was coming from due to the caesarean section that was supposed to be conducted there then I come here I am starved more until I give birth. So the communication wasn't good it would have been better if they had given me a timeframe to say from this time we won't be able to attend to you so you can eat something we will let you know when you can start starving but there was no communication in that regard until my husband became upset and I also became upset then my blood pressure started shooting again, that is when they had to take me into consideration. So communication wasn't that good.

Interviewer: I am sorry about your terrible experience, what information did they give you before taking you to theatre?

Participant: since I already knew my condition I was just told that now we are going to theatre ok let us go. They took me that is all then the procedure began.

Interviewer: Please describe to me the process of giving consent for emergency caesarean section.

Participant: usually they just tell you your case is like this like this do you want caesarean section or not then they give you a form you go through then if you are willing you sign I don't know but mostly the only option is to sign.

Interviewer: What was written on the form?

Participant: I can't really remember the words exactly but it was like myself you write your name then you say you give consent to the caesarean section that is about to happen and the other words I can't really remember what was written but it explained everything that was to take place.

Interviewer: What factors did you consider before signing the consent form?

Participant: the major thing that was just on my mind was for me to be ok and the baby to be fine. That was the only thing that was on my mind. So even if I was signing whereby I knew my condition so it was easy for me to sign. I already knew that this is what was happening to me and the only way out to save my child is through an emergency caesarean section that is why had to sign the consent form.

Interviewer: Were you anxious the period you were waiting to be taken into theatre?

Participant: yes I was anxious because whereby you see people who have just been admitted being taken to theatre. So I couldn't figure out what was happening I think that is what made my blood pressure to start rising why aren't these people attending to me as soon as possible.

Interviewer: What information do you think would have calmed you down?

Participant: The information I would have wanted was for them to say from this period to this period we won't be able to attend to you. Then for now ok they just explain that we are having conditions for this and this and this very critical at least they explain in that manner unlike whereby I am just waiting idle without knowing what is happening they come and pick this

one, they come and pick this one they go just like that while I am just sitting there, communication could have helped.

Interviewer: How did the lack of communication make you feel?

Participant: I felt neglected of course like my case was not important because I was also fighting for my life whereby they are just quiet, it made things worse.

Interviewer: Could you describe the communication with medical personnel after you came back from theatre please?

Participant: I was taken to where they put patients from C-section I was asking about the baby because it was too big but they said she was in [removed]. The problem that I had with them was that they couldn't allow any family member of mine to go and see the child maybe to say this this is the baby that has been brought whereby even my husband was denied. So due to the pain I had I couldn't manage to see the baby and know this is my baby. From there I was brought here drugs were given but I couldn't see any of my family members until the morning nayo yachiba yaku donsela whereby they won't give you time to see them or talk to them bwino bwino (nicely) to say how are you just like that. Then the worst experience is that having a fresh wound then there is no one to help you around so you do everything on your own. When the nurse comes you tell them I am feeling like this they say no you should be moving yes you should be moving but like in the first day where you are just from theatre and you are in pain and have a fresh wound you need someone to help you what if it [surgical site] opens up due to the pressure I would be putting myself I need to be moving I need to be moving. So I find it challenging.

Interviewer: what other information did they give you when you came back from theatre?

Participant: when I asked about the baby they said since the baby was too weak they wanted to conduct some tests to check if the child is ok maybe she has some underlying problem. That is all. Afterwards they were like the baby is just fine. They also told me that I am supposed to lie down for eight hours.

Interviewer: I have noticed that most women are signing the consent form without really understanding it, what recommendations would you make?

Participant: I think the best time that can be done is during antenatal visits then when also when you are admitted when you go into labour at least they explain to say if such a thing occurs to you they repeat the information so that you are psychologically prepared in case such things happen whereby you are expecting to deliver normally then suddenly things go wrong at least you would be prepared psychologically.

Interviewer: what impact does signing of the consent form have on you when you do not fully understand it?

Participant: it is terrifying because you are asking yourself am I going to come back alive, is my baby going to be ok or are we both going to be ok, so it is kind of traumatic and terrifying at the same time. At least maybe if the mind is prepared it would help.

Interviewer: Anything else you would like to talk about regarding the patient – health care provider communication?

Participant: They need to improve the communication and the wellbeing of the patient especially after the caesarean at least there should be a bed sider to ensure that the patient is fine until they are able to do things on their own then they can let go of the bed sider not whereby I am weak I am a patient and I am struggling to do things on my own I need help from someone. I can't bend down or do strenuous things due to my operation so it would help a great deal if this can be improved.

Interviewer: Thank you so much for your time.

Participant: Alright, you are welcome.

PARTICIPANT NUMBER 21

Age: [early 30s]

Occupation: Business lady

Religion: Christian

Highest level of Education: grade 10.

Marital status: Married

Interviewer: How many children do you have?

Participant: Three.

Interviewer: Was this your first emergency caesarean section?

Participant: this is the second one.

Interviewer: What was the cause of both caesarean sections?

Participant: The first one the baby was breech, the second one mmmm actually this one it was due to mmmm I took a long time in labour, I was given something for forced labour but it didn't work out so they just decided to take me to the theatre yeah. Actually at [hospital name removed] they did the caesarean section but couldn't take out the baby. They had to call [removed] to say they had an emergency so I came here with an open wound and the baby was still inside when we reached [removed] that is when they baby was taken out.

Interviewer: What was going through your mind when you were being moved with an open wound and the baby still inside your womb?

Participant: hmmm I didn't react I was actually too tired because I took too much time.

Interviewer: What was your reaction when you had your first emergency caesarean section?

Participant: Actually I was scared because I didn't even know how it feels, I didn't even know the experience over that so I thought I was going to die but I was told no you will be fine and the baby will be fine, you won't feel anything by the time you wake up you will just find the baby beside you yeah and that was what happened exactly.

Interviewer: What information do you think would have calmed you down or allayed your fears in your first operation?

Participant: Actually I was calm because I was told everything will be fine, nothing is going to happen there will be no complications whatsoever so I was being encouraged before the operation was done yeah. So actually they were just talking to me by the time I looked they showed me the baby so I said wow so this is what happens, they said yes so I said ok. When having my second operation actually I wasn't scared. I thought the same thing was going to

happen but this one was different yeah this one was really different because I was in so much pain actually this one I went through pain yeah.

Interviewer: What information did the health care providers give you before you were taken into theatre?

Participant: when I came here it's like everyone was surprised to say hey who does this, how can you bring a patient with an open wound and the baby inside? This is very dangerous, the baby can die or we can lose both the baby and the mother because the chances of surviving here is 50-50. So but I thank God that they did everything, they did it fast just to save the life of the baby and my life yeah.

Interviewer: So were you not scared being moved from [hospital name removed] to [removed] with an open wound?

Participant: I was scared I felt this is the end you know mmm ah I was very scared I can't lie I was very scared. I just thank God that I am still alive today. It is just by God's grace.

Interviewer: What kind of information do you think would have allayed your fears or anxiety?

Participant: Actually at that point no one was talking it was going to be better if at least someone was talking to me like no you should not get scared we will do everything possible so you shouldn't get scared but it was like they were also scared because they were saying hmmm the chances are 50-50 here so ah everyone was like ah you know so even me myself I lost hope but I thank God that everything worked out for good.

Interviewer: So did you sign any consent form before the operation?

Participant: Yes I did

Interviewer: What was written on it?

Participant: **(**she hesitates**)** hmmm actually I didn't sign the consent form I just did this where you put ink on your thumb and press on the form (thumbprint).

Interviewer: Even a thumbprint is considered signing, what factors did you consider before signing the consent form?

Participant: I signed the consent form because emergency caesarean section was the only option for me to be given help yeah.

Interviewer: After you came back from theatre what information were you given?

Participant: Actually no advice was given, the only thing I was told was that the uterus has been removed and that I won't be able to conceive again, it was the only thing I was told. So I said ok it's fine from there no one has talked to me or has given me any advice on what to do yeah, on how to go about it no one.

Interviewer: How does that make you feel?

Participant: you know it feels bad but what can I do? Nothing, it feels bad but there is nothing I can do since there is no one to advise me so I just comfort myself ha ha.

Interviewer: What sort of advice would you have loved to hear?

Participant: In a situation like this at least you need someone like to comfort you like to tell you, you don't have to worry everything will be fine, there is a God in heaven who works wonders you might not know what is going to happen tomorrow. Something like that yeah.

Interviewer: I am so sorry to hear that. Did they make you sign for the removal of the uterus?

Participant: No, I didn't sign for that they just made a decision because they said it (uterus) was damaged, it was worse they couldn't repair it so they just had to remove it. I remember they told me to lie down for eight hours and how to clean the wound

Interviewer: Who provided you with the consent form?

Participant: the nurse was the one who handed over the form but doctors were also there.

Interviewer: As we conclude this interview is there anything of importance that you would like to talk about regarding patient-health care provider communication?

Participant: Yes for women who have emergency caesarean section many times whereby women go for caeser they go there maybe he she lost the child then she she has just been left with the what, the wound, a lot get depressed you know. That is where you find a situation whereby bp, stroke so at least if someone is in this kind of a situation she needs to be counselled it is better she has to be counseled you counsel the person you at least counsel her you advice

the person just talk to her encourage the person I think this is what should be done because women come out from there without being counselled and that is not good. No wonder you find that people get depressed others end up committing suicide because of the same but when you are being counselled at least you have that hope to say ok if it happened next time I will conceive again and nothing of the sought will repeat itself. I feel the counselling is not there. I wish they had counselled and encouraged me after they removed my uterus.

Interviewer: Your conclusion on experiences of communication with medical personnel before and after emergency caesarean section?

Participant: I think on that one communication is very important yes they have to communicate with the patients and also us the patients we have to communicate to them so that we go along.

Interviewer: Were you free to ask health care providers any questions?

Participant: no I didn’t even ask them any questions because I just didn’t feel like yeah.

Interviewer: Thank you so much for your time.

Participant: You are welcome.

PARTICIPANT NUMBER 22

Age: [early 30s]

Occupation: Business woman

Religion: Christian

Marital status: Married

Highest level of Education: Grade seven

Literacy: Not able to read and write

Interviewer: How many children do you have?

Participant: I have four children.

Interviewer: Was this your first emergency caesarean section?

Participant: Yes, it was the first operation.

Interviewer: What was the cause?

Participant: the passage (birth canal) was there but the baby wasn't coming out.

Interviewer: So what was your reaction when you were told you would give birth via caesarean section?

Participant: I felt good because I suffered a lot and wanted to rest.

Interviewer: Were you scared?

Participant: No, I wasn't scared.

Interviewer: Could you describe the communication with medical personnel before you were taken into theatre?

Participant: Ah they were just talking to me nicely because they tried everything including forced labour with hope that I would deliver within two hours but it failed. So in the end I just asked them if they could just perform caesarean section then they said we can but we need to wait for your blood pressure to drop. So I begged them to just do the caesarean section because I was very tired.

Interviewer: Did you come here directly to give birth?

Participant: No, I was referred to [hospital name removed] from a local clinic.

Interviewer: What information did health care providers give you before you were taken into theatre?

Participant: They told me that they had tried all options available but that caesarean section was the last resort so I told them I was fine with the surgical procedure as long as I feel better because I was very tired.

Interviewer: What else did they tell you?

Participant: there was nothing.

Interviewer: Did you sign any form before you were wheeled into theatre?

Participant: yes I did.

Interviewer: What was written on the form?

Participant: They (healthcare providers) just told me to write my name and the doctor put his name as witness.

Interviewer: Ok but what was written on the form?

Participant: mmm I didn't pay attention because I was in pain.

Interviewer: What factors did you consider to sign the consent form?

Participant: mmm it was tough ha ha ha I went through so much pain, it was very painful so I had no option but to sign. Hmm you can die blood pressure is not visible it is just rising so I really wanted to be out of that situation.

Interviewer: Since it was your first operation, were you scared?

Participant: No, I wasn't scared, I just wanted to survive mmm and for sure I survived yeah.

Interviewer: When did the operation take place?

Participant: On 6th April.

Interviewer: So what information were you given after the operation?

Participant: After the operation they asked me the sex of the baby and I said it was a boy. So they told me they were taking the baby to [removed] because he was tired. So I said ok. Then they said I will be taken to the ward. So I said thank you very much may God bless you I have survived.

Interviewer: After the operation, what did they tell you?

Participant: Ah I had a bandage so they told me to lay down straight I was brought to the ward around 11 hours, I didn't drink any water, I didn't even eat anything until 19hrs then they told me to drink a bit of water. Then I felt a lot better and that is how they brought me here yeah.

Interviewer: What reasons did they give for telling you to lay down for eight hours?

Participant: Because the medicine had not yet worked properly yeah.

Interviewer: Describe the communication with medical personnel after theatre?

Participant: No, they treated me well and spoke to me nicely as a patient is supposed to be handled.

Interviewer: What would you have loved to hear from nurses and doctors after you came back from theatre?

Participant: Ah nothing because they worked on me and I have seen a lot of change I didn't feel any pain after I came from theatre, I just had pain from the surgical wound but there is a lot of improvement and I am very grateful to the doctors for saving my life and my baby's life. So everything was just ok.

Interviewer: I want you to focus more on the aspect of communication.

Participant: Ah we just talk fine. The nurses and doctors greet me and ask me how I am doing and I tell them I am fine. They also tell me how to clean the wound.

Interviewer: What would be your concluding remarks?

Participant: No, I am just grateful to you, you asked me questions and I answered.

Interviewer: Ok, what questions do you have for me?

Participant: Why are you conducting this study?

Interviewer: I am doing this study in order to document patient – health care provider communication and inform changes in the care for women who undergo emergency caesarean section as you are aware the procedure is traumatic.

Participant: Ah that is good. So are you are from the government?

Interviewer: No, like I said before we started this interview, I am a student at the University of Zambia but the government uses research findings to inform policy decision making.

Participant: Ok.

Interviewer: Do you have any other questions?

Participant: No

Interviewer: Thank you so much for your time.

Participant: Thank you too for talking to me.

PARTICIPANT NUMBER 23

Age: [late 20s]

Highest level of education: Certificate in entrepreneurship

Religion: Christian

Marital status: Married

Interviewer: How many children do you have?

Participant: I have one.

Interviewer: this same one?

Participant: Yes.

Interviewer: is it a boy or girl

Participant: it is a boy.

Interviewer: Congratulations. How was the operation?

Participant: Thank you. The operation was ok I would say yes ah I thought it would be the worst but it went well than I really expected.

Interviewer: What was the cause for emergency caesarean section?

Participant: Ok ah I was really failing to push at first my labour started on Monday in the early hours of Monday so I was admitted around 10, 11 hrs somewhere there. So when I was checked the doctor kept on telling me no you will deliver. I was just going 2cm after some few hours they come to check it is just 2cm so ah the doctor decided to induce me saying it will really open up the cervix and at least to contract more so that you give birth and looking at my body weight he tried to avoid the caesarean section and all that. So after sometime that was on Tuesday, Monday I slept here Tuesday around 14hrs I was induced then the pain started around 16 to 17 hrs so I was really in pain. I have never experienced that but you know that experience of ya I don't know how I can describe it. I was in pain I

don’t know if it was labour pain or the medication and I kept on pushing the nurses and the doctors were there to help me push but I couldn’t push I was told the head was too big so my bones they couldn’t open. So after 21hrs when the doctors checked they said no, the fetal heart of the baby is really faint they can’t pick it up so they decided just to do a caesarean so that they save the life of the baby. So that is how I ended up in the theatre.

Interviewer: When was that?

Participant: that was Tuesday around 22hrs.

Interviewer: Were you nervous?

Participant: No, I also decided to tell the doctor that this is too much just take me to the theatre and do a caesarean section because really I don’t think I will manage because I was also tired. It was my decision anyway even when the doctor was telling me that I was already decided in my heart.

Interviewer: Were you scared since this was your first time to have emergency caesarean section?

Participant: No actually I wasn’t scared I was just eager to see my baby ishi it is a rough journey from day one where I was whatever will happen will happen I just wanted to see my baby. So I wasn’t.

Interviewer: so how would you describe the communication with medical personnel before you went into theatre?

Participant: I would say my communication with them was good. Yes from day one that I was admitted here. There was this nurse who kept on encouraging me when I was in pain. She was saying no don’t do that if you do that the baby and all that it was really good.

Interviewer: What was good about it?

Participant: Hmm it was just good.

Interviewer: What information were you given by health care providers before going into theatre?

Participant: Ah actually there was no information given to me and I was really in pain and I kept telling them you take me to theatre so there were just encouraging me you do this do this ok and I was in pain I kept on demanding for a painkiller they said no if we give you a painkiller now it will affect your baby. You find that if you give birth the baby

will go to [removed]. So I said no I don’t care about that just give me the pain killer they kept on saying don’t worry you will be fine.

Interviewer: What information do you think would have calmed you down?

Participant: the information that I would have loved them to tell me was actually let me say they gave me all the information. But I would have loved them to explain the reason why we want you to do this is because of this and that but I feel like there is a certain part where they hide that the baby is big all that. Of course the baby was big but not that much I feel there was something that they couldn’t disclose as medical personnel yes.

Interviewer: What information?

Interviewer: the information I would have loved them to tell me, I think there was a big reason why I failed to deliver normally but they kept saying the baby’s head is big but the weight of the baby was 3.4 kg so I think there was something they were really hiding and didn’t want to tell me. They were saying the bones cannot open up. But I really wanted to know why the bones were not opening, could it be my age or what? That is the information I would have really loved to hear from them.

Interviewer: So at what point did you know you were going to deliver through emergency caesarean section?

Participant: That was after the nurse decided to call the doctor to come and check me to do a recheck yes because I was really in pain and the water broke but the baby was not coming and I was still on 4cm so that really got them worried to say when the water breaks that means the head is about to come out so that was ah I think that was it yes yeah.

Interviewer: So what was your reaction when you were told you are going to deliver via caesarean section?

Participant: Actually I was really happy because the pain I was feeling I have never felt that pain so me going to that side (theatre) I was really happy to say I would be relieved because even my mum is a nurse here she works in [removed], which is [removed] so she was there to comfort me, I told her that the pain is too much for me I think I am going to die and she was like no you are not going to die but once you go that side (theatre) they will give you an injection on is it spine she said yes you will feel better so I was really anxious to really get there and them give me that injection so that I feel better.

Interviewer: What information do you think would have made you feel less anxious?

Participant: I think at that time I didn't want to hear any information apart from telling me that now we are going that side (theatre) you will be fine, the baby will be ok and all that.

Interviewer: What really matters to you?

Participant: what matters to me is seeing my baby, seeing my baby grow and actually even me getting better because I am in a bit of pain I would love to get better and stop relying on my relatives to change the baby's nappies and all that. I would love to get better and take care of my baby.

Interviewer: Please describe to me the process of giving consent for emergency caesarean section.

Participant: wow ah on that one I really don't know the process of giving consent but on my part I think it is good depending on yourself and what you are feeling. For me giving that consent to say you people really do this for me I think it was really nice on my part because if I delayed I would have lost my baby or probably lost my life. So I think that is the way I would describe it myself.

Interviewer: Were you given any form any consent form to sign?

Participant: yes that ah a form of consent you sign to say I have willingly decided to deliver through caesarean section.

Interviewer: What were some of the contents of the consent form?

Participant: Actually the pain that I had to say the truth I didn't have time to read I was just told sign here and I signed. It was done yes.

Interviewer: What did they explain to you about the consent form?

Participant: Actually the sister (mid wife) didn't have that time to explain because even her she was told by the doctor that let us do a caesarean section on her so even her she was busy rushing to do this and that because I had no catheter they had to put what is this a cannula on me so she was alone in the labour ward with me so she didn't have that time to explain what was written on the paper there yes. So she just said ok sign here to show that it was your decision and willingly you have to do this. So I got the pen and signed. Then she started preparing me to go to theatre.

Interviewer: What are some of the stories that you have heard about emergency caesarean section?

Participant: Actually the story that I have heard from my mum being a nurse and she works in the labour room so she was telling me there is a lady that came she had high blood pressure, she would get swollen and all that so the time she was supposed to deliver she was told to go to theatre and then she kept on refusing so ah by the time she was taken that side they found that the baby was rotten so that really got me scared but when I reached there I never even thought of that. I just wanted to get there and have this thing down on me. I really had hope that when I go there I will be ok.

Interviewer: What made you to have hope?

Participant: I have a young sister she underwent an operation. She had appendix (Appendicitis) she had it removed because she used to get swollen though ah she also used to refuse to go there (theatre) but the time she went there she was operated on and today she is ok, she is better, she is a medical student so that was what really motivated me that I will be fine yes.

Interviewer: Kindly describe to me the communication with medical personnel after you came out of the theatre.

Participant: Actually my communication with them was good with the doctors that operated on me. Immediately we reached there after the doctors did handovers, the doctor took me in started putting these ma cannulas he was very good he said let me do this to help you get better. After they were done I thanked them to say thank you if it weren't for you I don't know where I would have been. They said we also thank you because you cooperated so that we help you. So communication was really good. They kept on asking how are you feeling do this don't do this.

Interviewer: What advice did they give you?

Participant: Actually the advice they game me before I was brought back to the ward that side (recovery room) a nurse was telling me that just lay on your back don't move you will have a headache and all that. So when I came here I got the same information to say don't lay on a pillow just lay on your back we will tell you after eight hours when to turn and all that so that was the information after theatre. Then this morning the doc (doctor) was telling me after he checked my wound you are ok just take some exercises don't sleep, don't sit you move around so that you get better. A process of healing you need to do exercises.

Interviewer: So how are the exercises going?

Participant: I am trying by all means, they have chased our bed siders and all that so I have to change the baby on my own, and I have to go to the bathroom. But though I am feeling pain I am able to manage.

Interviewer: Apart from giving you information on exercises, to lay down for eight hours, what else did they advise you?

Participant: The other advice I was given was no when do this you will have a bad headache because I was tired I wanted to turn, I wanted to hold my baby and all that but I was told it is better to listen to the advice and then later on you will see your baby its better you are well so that you look after your baby and all that. Then on how to take care of the wound I haven't yet been told because they haven't told me when they will discharge me so for now I think the nurses will take care of it.

Interviewer: What information are you really itching to be told?

Participant: Hmm actually when I would be discharged, I can't wait to go home.

Interviewer: Apart from being discharged what other information would you want to be communicated to?

Participant: I think the other information I would love to hear is to be told that you are able to do this and that I would love them to tell me what type of food to eat because I was told that there are certain food stuffs I shouldn't take yes. That is the information I would love to hear.

Interviewer: What were the reasons they told you not to eat solids?

Participant: hmm on that one I am not sure, they didn't tell me the reasons but I was just told to take fluids, orange and just those tuma lighter food stuffs.

Interviewer: What complications did they say you would experience if you did not lay down for eight hours?

Participant: they said I would have a headache but I don't know the connection. They told me just to lay down and that if I did this and that I would have a bad headache. So I am not really sure.

Interviewer: As we come to a close of this interview, is there anything of importance that you would like to talk about regarding communication with medical personnel before and after emergency caesarean section?

Participant: I think ah the other part of communication I would love to be changed is there are some nurses that are rude ah you can't tell them to say ok I don't know if they know that or what but for them to be medical personnel they know that so when you beg them they are supposed to listen, they are supposed to have a listening ear, they have to attend to you because we have read papers in the hospital to say a patient has got a right. So when you complain if it is not making sense to them I would rather they explain that when you do this and that it will affect you this way (consequences of not doing certain things you are advised to do) so than them some other nurses come out to say I am not the one who impregnated you, such words are not good. So I would really love to see a change on that.

Interviewer: Do you think that the information they gave you is enough?

Participant: yes I think it is enough because it is really helping me when I follow what they are telling me. It is really helping because I wasn't able to wake up but when I was told to lay down for that eight hours and after sometime they told me that you can wake up so it is really helping me yes.

Interviewer: What would be your concluding remarks on communication with medical personnel before and after emergency caesarean section?

Participant: I will just thank you for coming to have me share my experience with you. I know it will go a long way and it will help others out there. It is not easy but with hope and prayer of course I would like to say I was praying at that time but if you have faith and pray about it, it goes well. So thank you for coming.

Interviewer: I really want you to focus on the aspect of communication.

Participant: as I conclude I would just say thank you to the doctors and nurses that communicated to me because the communication between me and them was really good if we had such doctors and nurses around I think the hospitals would be a better place because we have heard stories of people complaining people not going to the hospitals in fear of what they will find there and how they will be attended to. But I would say let us try it is not everyone who is like that. The bad experience you had with a doctor or nurse would happen again but this is where we get help.

Interviewer: What information have you been given on future pregnancies?

Participant: hmm that one I would lie I wasn't given. So I would just say the time I was induced the doctor said just looking at your body, you are huge and having a caesarean

section on you it will really affect your body because you are fat and your recovery won't be good looking at that it is your first pregnancy we don't advice that. We hope to try and deliver this baby. So hmm I think that is it.

Interviewer: So are you worried about what the doctor said?

Participant: No, I am not worried because just the way I am feeling I have already healed. The wound is not big it is just a ka small cut they did so it is not really big such that I can worry may be it will affect me in future no.

Interviewer: Thank you so much for your time.

Participant: Thank you.

<u>PARTICIPANT NUMBER 24</u>

Age: [mid 30s]

Highest level of education: Diploma

Occupation: Stenographer

Religion: Christian

Marital status: Married

Interviewer: Was this your first emergency caesarean section?

Participant: yes.

Interviewer: How many children do you have?

Participant: This is the second one.

Interviewer: What was the cause of the emergency caesarean section?

Participant: fetal distress.

Interviewer: What was your reaction when you were told you were going to deliver via caesarean section?

Participant: Hmm my reactions were good because I was already in labour and I was expecting to have a normal delivery and I was told it was not possible because the baby is breath was low and they were worried about it. They said you might lose the baby or have it. The baby will have difficulties breathing or after delivery the baby will not cry so we will have to resuscitate. So it will be a 50 – 50 chance because they couldn't wait for my labour it was moving at a very slow pace so the worry was my baby will be stressed and then for us to wait for the labour process it will take long. So it is better we do caesarean

section, the emergency one even inducing because we have induced also have contributed to stressing the baby. Yes.

Interviewer: So were you nervous?

Participant: Yes, I, no I wasn’t but I was just sad that I was going to have a caesarean section but I had accepted that if that is the case then that is fine. Let’s do it so that we are safe together with the baby. I was scared for it but I wasn’t scared.

Interviewer: You mentioned that you were scared to undergo emergency caesarean section, what sort of information do you think would have allayed your fears?

Participant: what the doctor said because she assured me don’t worry you will be fine and the baby will be fine. Ah then I was assured that you are going in theatre just now because we had a challenge with the theatre it was fully booked a lot of people were supposed to go for caesarean section. They assured me that yours is very emergency we will go there and then. For sure it was very emergency I went in in the shortest possible time. But it was painful.

Interviewer: Please describe the communication with medical personnel before you were taken into theatre?

Participant: the communication was just ok at least the doctor who was attending to me the one who assured me she was quite ok we really communicated well and she really acted professionally.

Interviewer: What information were you given before you were taken into theatre?

Participant: She assured me that you would be fine together with the baby if at all the caesarean section will be down from now so don’t worry. She reassured me I think that is the only information she gave me.

Interviewer: Describe the process of giving consent for emergency caesarean section.

Participant: Ah it was, since it was emergency yeah the consent was given to me so I thought now my husband could come and sign but he was at work. The time I was calling him he left the phone in the office. So I had to call the colleague to look for him that it is emergency but despite that the trolley was already there waiting for me. So I couldn’t wait for him so instead I had to sign.

Interviewer: So what was written on the consent form?

Participant: the contents were I was supposed to sign in case of anything, in case of eventualities.

Interviewer: Who read out the consent for to you?

Participant: I read it myself.

Interviewer: Apart from the part where you are supposed to sign what other things were written on the consent form?

Participant: Ah the other things were I was acknowledging in short that I have accepted to have a caesarean section in case of any unforeseen there is no one who is going to be blamed.

Interviewer: Who provided the consent form?

Participant: The nurse of course gave it to me. Then I read and signed.

Interviewer: how would you describe the communication with medical personnel after you came back from theatre?

Participant: ah it was ok, it was just ok. The communication was just fine just like it was before.

Interviewer: What was ok about it?

Participant: they gave me instructions of course concerning somebody who has come out of caesarean section, like you should wake up, I had to lay just like that. I was told after six hours that is when you wake up we will come and inform you when to wake up and the consequences if I wake up before six hours I will have a severe headache which will affect me because the injection which was given here behind my back it was a lumber puncture so to say that made me to be numb so that as they are doing the caesarean section I am not feeling the pain but though I felt the pain because the other leg took long but they couldn't wait they said at least the other one is numb even this one bit by bit it will be. They assured me that I experienced that because it was done fast, quickly. But they really took good care of me while in theatre and the communication was ok even after the caesarean section**.**

Interviewer: Apart from telling you to lay down for six hours, what else did they tell you?

Participant: after six hours I was told to turn, I can turn to any side. I was told to have something warm like tea and I was told now you can wake up and start walking of which I did. I had two cups of tea but I was laying on both sides, I was able to turn in short.

Interviewer: Earlier, I heard you talk about solids.

Participant: yes I was told that I can have solids, in the morning I had tea with bread they said it is fine and they are encouraging me to have a lot of fluids and fruits.

Interviewer: what were the reasons for them to tell not to have solids?

Participant: hmm I think the reason is more like because of what I went through and I shouldn't just start to take hard stuff which might bring other complications. So bit by bit because I took long, yesterday I didn't eat better I started with fluids tea at least because it is more like yesterday the whole day I didn't eat.

Interviewer: As we come to a close of this interview, what would be your recommendations with regards to communication between women who undergo emergency caesarean section and health care providers? What kind of scenario would you love to see in terms of communication between the two parties?

Participant: I would recommend my fellow patients because that is where I think most of the challenges are because sometimes they don't understand these medical personnel we would want to do our own way but them they know what they meant. Like in my case exactly what they assured is what happened despite they are just human that is why there is that consent form in case of eventualities that might come but it is better to listen to them because they have experience, they have experience in their area of study through these same practicals and when they say this the fact that you are not the first person to go through that. It is important that us patients abide by what they say. Like sometimes most of us come with a bed sider they might say no no meanwhile you the patient has said yes you find that the person you are with discourages you to go through that process (caesarean section). So I would encourage my fellow patients to listen and take advice from medical personnel.

Interviewer: with your experience, what aspect of information would you love improved?

Participant: Ah like on this one ah would I say sensitizing because these are just emergencies I don't think if I say sensitizing the people would be a word no, I think the communication before the emergency caesarean section is just vital and maybe because we are calling it emergency caesarean section so I think on that one even communication it's there and then like the way it came to me it was just there and then and I had to go although it is not easy for somebody to accept in the shortest possible time like I did. So in terms of communication you really need somebody who is fast in thinking otherwise if you say let

me think somebody about to go in theatre they prepare their minds mentally and physically but this one hmm it was just there and then. So it is very difficult for others to understand it.

Interviewer: To understand what?

Participant: the same, to accept emergency caesarean section because of the shortest possible time so that in terms of that it could be improved in the sense that they inform you in advance.

Interviewer: When do you think would be the best time to talk to these women about emergency caesarean section and the consent form?

Participant: I think during antenatal because you would never know that you will go through this. So I think that could be recommended even during antenatal when they talk about caesarean section because they come for antenatal they will tell you are are in this week next time you come it will be late labour so next time you come we will admit you or induce, book for induction or caesarean section because you have proceeded the period. So I think before that women should be sensitized that there is such (emergency caesarean section) because me that was news and it was late for me apart from the caesarean section (planned) I know. So I think us women we need to be sensitized on late delivery, emergency caesarean section and this normal (planned) caesarean section so that we are educated. To me that one (emergency caesarean section) was news because it was my first time. I know there is caesarean section but I didn't know there is emergency. But so we need to be sensitized and have knowledge of some kind, during antenatal visits they explain to us, early labour, late labour, emergency caesarean section, normal caesarean section, inductions so that we have an idea or knowledge about it so that when I am being told about emergency caesarean section I would have already learnt about it.

Interviewer: do you think that would calm you down if you had prior information about emergency caesarean section?

Participant: Ah something which comes as an emergency it is always shocking but when you have heard about it you are aware you know something about it. It helps.

Interviewer: And about the consent form, when would be the best time to tell women who undergo emergency caesarean section about it?

Participant: even the consent form should be part of the teaching at antenatal clinic because you need to know others don't know that there is a consent form. Because even unfortunately after somebody has died it is everyone who knows that there is death certificate given so even the consent should be also taught about there is this when you are going for a caesarean section, the people who are supposed to sign are this and this but in case of emergency you can sign yourself or any other person who is with you. Sometimes I hear even the doctors sign if there is no any other relative and if that person is an able to sign. So that also should be taken into consideration.

Interviewer: how did you feel when you were signing the consent form? Did you really understand it?

Participant: Ah me I just said anyway whatsoever outcome but the fact is I have been assured that everything will be fine but they made me sign because it is a mandate I wouldn't have gone into theatre without signing that.

Interviewer: Apart from it being a mandate what other factors did you consider when signing the consent form?

Participant: Ah safety of course, I considered safety of myself and the baby. That is the part I figured out. At the end of the day you might be saying no shani shani (blah blah) then you regret. But otherwise I was told if I wasn't told it would have been different but this is a procedure or a guideline which should be followed.

Interviewer: As we end this interview is there anything of importance that you would like to talk about on the aspect of communication between patients and medical personnel?

Participant: hmm aspect of communication us who come here to seek medical attention these people have been entrusted with handling people's lives. I don't think three quarters of them their intention is to harm us but they are giving us the correct information which we need. So I think we just have to listen to them and get the advice and follow because in most cases like I was told I am going for emergency caesarean section somebody can even run away then at the end they lose the baby or something unusual happens. Who do you blame when you were told you see then you go out there to say [name of hospital removed] is not good they killed my baby. They didn't take good care of me I lost my baby because of them which is not true.

Interviewer: Thank you so much for your time.

Participant: you are welcome.

PARTICIPANT NUMBER 25

Age: [mid 20s]

Marital status: Married

Occupation: House wife/currently unemployed

Highest level of Education: Degree in Environmental Health

Religion: Christian

Interviewer: Was this your first experience of emergency caesarean section?

Participant: yes it was my first experience of caesarean section and ha ha also of giving birth. This is my first child.

Interviewer: Great, how was it?

Participant: Wow ha ha ha I didn't expect that I would be giving birth this week I was three weeks away to the due date. So I was admitted for in pregnancy induced hypertension when I got to 36 weeks of pregnancy my blood pressure just rose and it kept rising. When I came they measured my blood pressure it was 157. So they decided they would have to admit me. They checked for gestational age. I was at 37 plus three so the pregnancy was term that is how they said they were going to induce me the following day that was the 30th they said if induction failed they would have to do an emergency caesarean section. So that very day on Monday they admitted me and did a few tests, here and there, did a scan, they connected me to the CTG to monitor the heart rate and then they said it was good enough for induction and around 14 hrs they gave me the first dose of miso…. (Medicine). I was ok until 18hrs that is when contraction started the doses were six hours apart and I was going to be given three doses. Around 20 hrs I was checked and I had dilated to 2cm they gave me the second dose. The third one was going to be at 02hrs so contractions kept getting` tense but it was not bad such that I even managed to get some sleep. Around 02hrs I went to see the doctor I was checked and I was still at 2cm so that was a bad sign. Then they told me that there is a possibility that we will

have caesarean section like I had stopped progressing. That is how at 08hrs they checked me again still at around 2-3 cm and then they decided to connect me to the CTG to check the baby's heart rate again. Then they said the baby's heart rate wasn't that stable. That is when it went into distress so that is how they took me for caesarean section. It seemed it was a very packed day, the theatre was busy so I ended up being attended to quite late. I went into theatre around 16hrs and by 16:29hrs the baby was out and she was fine, she is fine. She was born at 3kg and everything was perfectly fine yes basically that is what happened.

Interviewer; what was your reaction when you were told you were going to give birth via caesarean section?

Participant: Ishi on Monday I was in shock they asked me if I understood what they said I said yes they asked me if I understood why they had to do it because I have some medical knowledge I know how dangerous hypertension is in pregnancy so I think before I came to be checked I knew that if my blood pressure was going to be too high it's a possibility, that was when but when it happened ha ha ha I was in shock really. I had to take time to process everything I think by Monday that is when I accepted I said whatever happens, happens as long as it is for the good of the baby and me.

Interviewer: What information do you think would have made you calm down?

Participant: Hmm ok I don't really know but I think generally in the country ah caesarean section is kind of frowned upon there are people who think that it could be very risk people think SPD is better so I think just information to the general public, that certain situations require the mother and the baby to be safe so I think there is so much misinformation about it because after the caesarean section was done I just said ha ha ha I think even my next pregnancy I will do a c-section it is quick though it was painless though I experienced a bit of pain when the anaesthesia wore out I think it was good (caesarean section)I would have risked the baby's life because when the blood pressure was high I had no signs and symptoms I was just fine doing everything normally, so I could have just collapsed and died. So I think there is just so much misinformation and something needs to be done about that.

Interviewer: I want you to focus on the aspect of communication, how would you describe the communication with medical personnel before you were taken into theatre?

Participant: the communication was really good because ah from word go they had explained to me what the options were and why we had to go that way. So I think the communication was quite good. It only became a problem ha ha ha on the actual day of the caesarean section there were so many cases coming in, there was just this long queue and I had to wait for a long time contractions hadn't stopped because I was already in labour, contractions hadn't stopped they were getting intense even if there was no progression so at that point maybe if they had communicated maybe reassured me a little because at some point I was so much in pain I just wanted to take myself in but generally the communication from word go I understood the risks I knew what I was going into.

Interviewer: What information did they give you before you went to theatre?

Participant: they did tell me about the type of anaesthetic they were going to administer to me, they told me they would have to inject me through the spine and then obviously they told me I might lose so much blood and require transfusion, they asked me if I have had a blood transfusion, if I have had any surgeries before all those things so I had to sign a consent form after they explained everything to show if I understood or not and I was allowed to ask questions where I didn't understand.

Interviewer: Please describe to me the process of giving consent.

Participant: ok the process of giving consent so ah as I said from word go they did explain to me the risks I was facing and why they made that decision since the baby was already term because the pregnancy is term at 37 weeks and I was at 37 plus few days yes so they explained that to me because I had lost the previous pregnancy at 12 weeks so they didn't want me to have that risk again where I could lose another child so we prevented that. Also they gave me the options that were there and each doctor that attended to me would explain and would ask me do you understand why we are doing this if I said no they would explain this is why we are doing this because you risk losing both you and the baby. The hypertension they carried out many tests to ensure there wasn't anything there I understood what they were doing and also on the nurse who came to give the consent form she asked me questions she explained to me and before I signed I knew what I was signing so I understood the entire process.

Interviewer: I am so sorry for your loss, what were the contents of the consent form?

Participant: I had to indicate my name because at that time I was alone in the ward so I had to indicate my name and who I was signing for ha ha I was signing for myself, I had to indicate where I was coming from I had to indicate if I had understood and I had to indicate what surgery I was signing up for yes I had to sign somewhere and there was also a witness obviously to show that I was in the right state of mind when I was signing yes.

Interviewer: What factors did you consider when signing the consent form?

Participant: Ah basically it's just my baby ha ha ha basically it was the life of my baby because I heard that she was going into distress yeah so I couldn't risk it anymore. I have never had hypertension before throughout the pregnancy my blood pressure was ok but all of a sudden it started spiking it could be at 140 but for the first time that Monday it was 157 so I knew that if I decide to let this go on I might just collapse especially that I had no symptoms whatsoever yes I took that into consideration. So my life and my baby's life were at risk. That is that.

Interviewer: please describe to me your experience of communication with medical personnel after you came back from the theatre until your discharge today.

Participant: after the theatre they took me to recovery room yes where they had to make sure that the anaesthesia was wearing off while I was there because I was feeling cold I do not know why ha ha ha but I think there is something they gave me which made me cold but they told me we have to keep you warm in here and they were also checking my vitals. So they explained that to me. They kept me there for hours and then they also explained to me after that I was going to have my first meal which was going to be liquid eight hours from there to here. Whilst in theatre they made sure I saw the baby I saw what baby I had they communicated to me how much it weighed so I had that knowledge even if I couldn't see hold her rather. So that part they did communicate. After I came here they explained I had to lay flat on my back so the care and the communication was there. After surgery and then the following day throughout the night they kept checking my blood pressure, checking my body temperature. Then the following morning they told me how to care for the wound so I had information on how to care for the wound all that and also any signs to look for yes they asked me if I was experiencing gastritis apparently it's common after caesarean section ha ha so they did explain which ones

were good signs and which ones were bad signs so they kept on checking on me and the baby up until today I am getting discharged.

Interviewer: What were the reasons they asked you to lay flat and also not to take any solids?

Participant: not to take any solids because I had major surgery and my body had to adjust so I that I don't over burden the digestion system. Then laying on my back that one wasn't explained ha ha ha they told me to lay on my back because at that time I had anaesthesia on my legs it hadn't worn off. So they didn't tell me why I had to lay flat on my back. They didn't.

Interviewer: Apart from laying on your back, not eating solids and how to take care of your surgical wound, what else did they tell you?

Participant: they also told me when they removed the catheter because they had put a catheter when drips are done I had to walk around so that it can help in the process of the wound healing. Also they told me not to strain myself yes but to relax so that the wound was quick to heal. That is the advice they gave me.

Interviewer: What recommendations would you make in terms of communication between patients who undergo emergency caesarean section and medical personnel since the procedure is traumatic?

Participant: so I think ok really it becomes difficult to communicate when there are so many misconceptions on caesarean section. Otherwise from my experience, communication was good enough. I understood everything that was going on so I couldn't refuse the procedure done yes. So the real issues are the misconceptions people have about caesarean section as patients. So you find that when you get here they will tell you the risks and everything, you know people risk losing their baby, people risk getting badly injured to give birth vaginally so really from my experience the communication was good. I can't lie they explained to me so many times why they had decided to go that route. So even my acceptance meant I knew what I was doing.

Interviewer: As we conclude this interview is there anything that you would like to say on the aspect of communication, which you haven't mentioned already?

Participant: Hmm I think I have said everything so far yes.

Interviewer: Thank you so much for your time.

Participant: You are welcome.

Interviewer: Congratulations on the birth of your baby once more.

Participant: Ha ha ha thank you ha ha.

PARTICIPANT NUMBER 26

Age: [late 20s]

Occupation: Marketeer (I sell in the market)

Religion: Christian

Marital status: married

Highest level of Education: Grade three

Literacy: Can't read and write

Interviewer: How many children do you have?

Participant: Hmm so far they are four. I have two children so plus the twins I am holding they are now four.

Interviewer: Was this your first experience of emergency caesarean section?

Participant: it was the first.

Interviewer: what was going through your mind when you were told you would deliver via caesarean section?

Participant: I was thinking of refusing.

Interviewer: Why?

Participant: Hmm I was afraid of the operation and just having a wound. So the medical personnel told me that it was important they saved my babies and me.

Interviewer: so what information did nurses and doctors give you before you were wheeled into theatre?

Participant: they told me they wanted to save my babies and me, agree.

Interviewer: What was written on the consent form you signed?

Participant: they wrote my children's names, they asked me how many children I had, they also put my age on the form then told me to sign. So I refused to sign. I told them I needed to first contact people at home. They waited for me but later I signed the form.

Interviewer: So did you understand what was written on the consent form?

Participant: yes I did.

Interviewer: What was written on the consent form?

Participant: they just wrote my age and the number of children I have.

Interviewer: so who told you to sign the consent form?

Participant: it was the nurse.

Interviewer: what information did they give when you came back from the operation?

Participant: hmm they didn't tell me anything. I am the one who had to ask them questions, but they told me how to take my medication and I don't know the other things they said.

Interviewer: When did you have the operation?

Participant: this is my second day after the operation.

Interviewer: so how are you feeling right now?

Participant: I am feeling just fine. But the surgical wound is painful.

Interviewer: What would be your conclusion on the aspect of communication with medical personnel?

Participant: I am just grateful that they saved my twins and me.

Interviewer: Thank you so much for you time.

Participant: Thank you.

PARTICIPANT NUMBER 27

Age: [mid 20s]

Occupation: Nothing

Marital status: Single

Highest level of Education: Grade nine

Interviewer: How many children do you have?

Participant: Only two.

Interviewer: Was this your first experience of emergency caesarean section?

Participant: yes.

Interviewer: How did it go?

Participant: It went well.

Interviewer: what was the cause for emergency caesarean section?

Participant: The baby was breech then after that I couldn't give birth well so no wonder they did the caesarean section.

Interviewer: Did you come directly here?

Participant: No, I first went to [removed] then was referred to [removed]**.**

Interviewer: Please describe your experience of communication with medical personnel before you were taken into theatre?

Participant: ah it was just well because they took me after they found out that I have to go to the theatre. The communication was just good, ok.

Interviewer: What was good about it?

Participant: they took good care of me.

Interviewer: I am talking about the communication aspect and not the care aspect.

Participant: oh on the communication ah it was just ok but mwe they took me to a room while there was another person in theatre. They told me they would take me inside after the one who was inside comes out.

Interviewer: What information were you given before you were taken into theatre?

Participant: There was nothing they told me.

Interviewer: So they just wheeled you into theatre without saying anything to you?

Participant: Ah ok they told me I have complications since this wasn't my first operation. So they told me I can't delivery normally unless I go for an operation.

Interviewer: But you didn’t mention this when I asked you earlier if this was your first operation.

Participant: ok I meant to say the first operation the baby was breech then the second one the passage was small and the baby was big so they said I should go for an operation.

Interviewer: So what were you told before you were taken into theatre?

Participant: there is nothing. They just told me that I would be next to go in after the one who was inside.

Interviewer: So they didn’t even tell you what to expect after the operation?

Participant: ok they told me that I didn’t have enough blood and that I needed to be transfused after the operation and that anything can happen.

Interviewer: did they give you a form to sign?

Participant: for going to theatre? Yes, they gave me the form (consent form) but I think it is with them. I haven’t seen it.

Interviewer: What was written on the consent form?

Participant: I was just required to sign the consent form because when going to theatre you need to sign it or your family signs on your behalf. The doctors and nurses can’t sign for you. They make you sign the consent form so that they are not blamed in case of anything. If you die they will say no no no here is where she signed or where her relatives signed. So it is like they want to keep the form as evidence.

Interviewer: So they didn’t explain what was written on it?

Participant: No, they didn’t explain and I was in a lot of pain so I didn’t pay attention to see what I was appending my signature on. They just told me to sign and I signed since it was a theatre form.

Interviewer: Were you not afraid signing something you don’t know?

Participant: I knew it was a theatre form.

Interviewer: What factors did you consider before signing the consent form?

Participant: They told me that I was experiencing complications and that it would be dangerous if the previous operation scar burst open. It is either the baby can die or both the baby and I can die. They told me that I just needed to sign the consent form so that they can save the baby and me.

Interviewer: Describe the communication with medical personnel after you came back from theatre?

Participant: like what?

Interviewer: What information did medical personnel give you after the operation?

Participant: kaili when you come from theatre you are drowsy so there is usually no communication. So they take you directly into a room (recovery room). When you are no longer drowsy that is when they ask you, how are you feeling? Then they bring you the baby when you gain strength.

Interviewer: Ok, what information did they give you after you came to the ward?

Participant: Ah here there is nothing they said, I am just waiting to get better I am still experiencing pain in the body. In fact I am waiting to be discharged from hospital. At the beginning they told me not to drink water unless after eight hours. Then they told me to take water and juice. But before you go to theatre you are not supposed to eat anything and after theatre you are also not supposed to eat anything. All you need to do is sleep for eight hours after the operation.

Interviewer: What reasons did medical personnel advance for laying for eight hours?

Participant: So that the surgical wound doesn’t get torn then also you can’t manage to sit up because you are heavily sedated and your legs are paralyzed.

Interviewer: Is that what health care providers told you?

Participant: they didn’t tell me the consequences of not laying down for eight hours. But they just said I should lay down that much.

Interviewer: Didn’t you ask them?

Participant: No.

Interviewer: Why not?

Participant: ha ha I was just waiting for eight hours to elapse so I kept on looking at the time on my phone.

Interviewer: What were the reasons for stopping you from eating solids?

Participant: because of the wound but they didn’t explain the reason but I just figured it out it is because of the operation wound. I think if you eat solids the wound would be overstretched when going to the toilet. So I think they tell you not to eat solids so that you stop standing to go to the toilet. They also told me to be washing my wound using lifebuoy

soap so that it remains clean. You can fail to walk if the wound is dirty because you will be in pain and it will become hard from the outside such that you can't stretch. But when you clean it you even manage to walk.

Interviewer: Ok, earlier you told me that you didn't read the consent form because things were being done fast, when do you think would be the best time to explain it to the women who undergo emergency caesarean section?

Participant: maybe they should also include a component of emergency caesarean section and the consent form during antenatal visits like the way they teach us how to take care of the baby. They should be able to teach us at antenatal so that we are able to process everything or at least be prepared mentally in an event that we are told to go for caesarean section. They should also give us a copy of the consent form so that we take to our husbands at home so that they are also prepared mentally so that they are not caught unaware. Like in my case when they told me to sign the consent form I first had to call my husband who expressed surprise. He said such things only my parents should sign because he was afraid that if anything happens to me he would be blamed. So I ended up signing the consent form because the nurses said I was running out of time.

Interviewer: Which aspect of communication do you think should be improved in this hospital?

Participant: Hmm I don't even know because to me everything looks normal because they are giving us medication in good time, they care for us. So I don't see any problem.

Interviewer: Thank you so much for your time.

Participant: ok, thank you.

PARTCIPANT NUMBER 28

Age: 19 years old

Occupation: House wife (stay at home mom)

Religion: Christian

Marital status: Married

Highest level of education: Grade 12

Interviewer: How many children do you have?

Participant: I have two.

Interviewer: Two? Are the twins your first babies?

Participant: Yes.

Interviewer: Congratulations

Participant: Thank you.

Interviewer: How did the caesarean section go?

Participant: Not so bad, it was ok…. (long pause) it was friendly.

Interviewer: What was the cause of the emergency caesarean section?

Participant: I was actually having twins but unaware so after being aware the pregnancy started to be in labour then they found out that I am going to have twins then they found out to say one of the twins was in breech and so they had to do an operation to save both the children and myself.

Interviewer: Describe the communication with medical personnel before you went into theatre?

Participant: it was ok, very much ok when you call for assistance or you need to find out something they would rush yes.

Interviewer: What about on the communication aspect?

Participant: on the communication aspect they use all languages that you are comfortable with. If you are comfortable with Bemba they will use Bemba if you are comfortable with English they will use English so any language you are comfortable with. The nurses were available.

Interviewer: And for you which language were you comfortable with?

Participant: I understand English, vernacular and Bemba as well.

Interviewer: So what information did medical staff give you before they took you to theatre?

Participant: I was told what to wear, how I am supposed to sleep on the bed, what I need to carry there (theatre) what they are going to need and what they are going to need of me to do when I am in there.

Interviewer: Since this was your first experience of emergency caesarean section, were you nervous or anxious?

Participant: I was nervous, yes I was very nervous because it was my first time.

Interviewer: What information do you think would have made you calm down or would have helped you allay your anxiety?

Participant: Speaking to nurses and doctors, them being close to me and giving me information and keeping me calm yes because they understand what is going to happen, if they give you information it becomes easier to calm down.

Interviewer: Did you sign any form?

Participant: Yes I did.

Interviewer: Please describe to me the process of consenting for emergency caesarean section

Participant: I was referred here so when I reached here they checked my papers and asked if I am ready to go ahead with the caesarean section and I said yes and after saying yes they explained to me the reason why I was supposed to go for caesarean section and that is when I was asked to sign the form with information described what was on the form.

Interviewer: Which hospital referred you here?

Participant: [removed]

Interviewer: What was written on the consent form?

Participant: They couldn't do the operation there because there was no doctor capable of doing it and there was also no doctor who could manage to do a caesarean section that is why I was referred here.

Interviewer: Ok but what was written on the consent form?

Participant: I can't say much because I don't remember I don't remember much of what was written on it.

Interviewer: So what was required of you?

Participant: What was required is my signature and also give addresses and numbers of family members who could be contacted in case anything so that they know who to call.

Interviewer: Did you understand the consent form?

Participant: Yes

Interviewer: What did you understand about it?

Participant: it meant that if I sign then I am saying yes to doing caesarean section at this hospital and whatever outcome comes out comes out.

Interviewer: What factors did you consider before signing the consent form?

Participant: I actually considered the life of the children and of course my own life because even when I was being brought here I was told the reason why I have to do caesarean section.

Interviewer: How would you describe the communication after you came back from the theatre?

Participant: it was ok.

Interviewer: What was ok about it?

Participant: I had nurses who were attending to me fast.

Interviewer: I mean the communication aspect.

Participant: the communication aspect was also good. It was ok.

Interviewer: What was ok about it?

Participant: The language they were using was understandable and they could easily reprimand you if you are doing something wrong. They tell you what you are supposed to do in a way that you can understand it.

Interviewer: What information did they give you after the operation?

Participant: I was told not to take anything, I was told the time to start taking liquids fluids, I was told not to move my legs and other stuff yes.

Interviewer: what was the reason for not moving your legs?

Participant: Because the medicine I was given hadn't yet settled in my body so if I had to move them harshly then it would mean that the medicine would move to my head and I would have a major problem by the time I am leaving this hospital.

Interviewer: What about the food, what was the reason for stopping you from eating?

Participant: because they had done an operation on my stomach and it is not yet strong to eat something strong. Then as for the fluids and water they were giving it to me in drips because it hurts when you take it directly when you from the theatre, the operation can hurt yes.

Interviewer: What other information did they give you?

Participant: Ah I can't remember much but this is what I can remember.

Interviewer: Earlier, you mentioned that you were afraid of the operation, why was that?

Participant: Ah I have never been to a theatre before and I haven't had any operation done on me. Before I had caesarean section I heard stories that the procedure hurts and it does

hurt. You lose a lot of blood and the likes if you are not taken care of properly you can have problems. So that is the reason why I was afraid.

Interviewer: what recommendations would you make with regards to communication with medical personnel and women who undergo emergency caesarean section?

Participant: maybe be for this hospital they use a lot of Bemba and Nyanja and we see a lot of people in here we are not all Bembas and Nyanjas yes. Maybe if there could be a diversity of language so that medical staff can give information that everybody requires regardless of the language the patients speak.

Interviewer: As we conclude this interview is there anything of importance that you would like to talk about regarding patient – health care provider communication?

Participant: not really because I didn't encounter any problems with the medical personnel. I might lie if I say this and that should be changed. Maybe it is just the issue of language which I have mentioned earlier.

Interviewer: When was the first time you heard about emergency caesarean section?

Participant: just when I was brought to [removed]. I didn't know about the emergency one but I knew about the planned one.

Interviewer: When do you think would be the best time to educate women on caesarean section?

Participant: As soon as the person is of child bearing age we should be educated about emergency caesarean section.

Interviewer: Thank you so much for your time.

Participant: Ok, you are welcome.

PARCIPANT NUMBER 29

Age: [mid 30s]

Religion: ha ha ha I don't belong to any because I can't call myself Christian because it's a big term which means Christ like and I am not to that point of Christ like.

Highest level of Education: Diploma in Tourism

Occupation: Service personnel

Marital status: Single

Interviewer: Was this your first experience of emergency caesarean section?

Participant: Yes, this was my first experience.

Interviewer: What was the cause of the emergency caesarean section?

Participant: Ah my water broke while I was in town. The cause of the emergency caesarean section was because ah I was told the baby is big and the head was slightly bigger than the 10cm they always measure so they just had to take me in for caesarean section of which it was very difficult for me to comply but they managed to convince me because I had a different perception of caesarean section, it was never my idea I never looked forward to having my baby through caesarean section because I believe that it is a sore that doesn't heal for a very long time until you die. The doctors explained the reasons why but I seemed to convince them that I give it a try that they should induce me but at the time that they were trying to induce me after being given medicine, they gave me the medicine but the baby could not breath properly that is how they just took me as an emergency yeah though it took time for me to cope with it but I had no choice. I had to save my baby's life. They were good to me I was handled properly even as much I was in the theatre they still explained and from there I just found myself I am awake

Interviewer: What was your experience of communication with medical personnel before you were taken into theatre?

Participant: The communication was fine I think from day one I had good doctors who attended to me and who took the time to explain what is going on with me and they were very patient with me. They tried their best and so far so good I think they did their profession very well and they made me feel comfortable.

Interviewer: What information did they give you before they took you to theatre?

Participant: they explained to me why I should take the ceasarean section even as much as I was opposed to the surgical procedure because yeah they went step by step to make me understand and they made me see the other side of caesarean section that it is not that bad but a thing that you undergo because you really need to survive life which is required. They explained to me in detail but I just kept on resisting to say no do me an induction but after that I had no choice but to accept because I was already prepared, I was already counselled and I had no choice but to go through it.

Interviewer: Were you afraid?

Participant: yes I was a bit scared but because I was in the hands of people whom I thought knew what they were doing so that made me calm, they were with me throughout the whole time and they made me feel comfortable. I didn't feel anything until the time I woke up.

Interviewer: What kind of information do you think would have helped allay your fears?

Participant: I think if we as women can be sensitized about the caesarean section so that they don't have a perception of it being bad and be sensitized about saving lives then that can be ok. They should sensitise women who might have a different perception like me.

Interviewer: Was this the first time you were hearing of emergency caesarean section?

Participant: Yes, it is my first time.

Interviewer: Please describe to me the process of giving consent to emergency caesarean section?

Participant: Hmm emergency caesarean section because you are pre counselled you know like they touch all points even from the time that they start with you when you have your pains and everything. They make sure you are checked from point one that determines if you are going for caesarean section or you will be induced or you will be what, so they make sure they lay the information properly of what you are going to go through. Then from there you have it your way but they don't really impose they listen to your reason but if it is an emergency if they say you have to go you just have to go. They try their best to educate you on that one.

Interviewer: Were you given any form to sign?

Participant: yes I was given a form to sign.

Interviewer: What was written on the consent form?

Participant: Hmm it was me giving them permission so that they can go ahead with the caesarean section yeah.

Interviewer: What else was written on it?

Participant: Ah it required a lot of things but when I remember I will let you know I was being taken fast even if I was already prepared so I just had to give the consent so that they can save the life of my baby.

Interviewer: What factors did you consider before signing the consent form?

Participant: The first thing I considered was the wellbeing of the baby yeah.

Interviewer: Mother's instinct.

Participant: Yes ha ha ha because you cannot carry the baby the whole nine months then come and lose it I would rather save my baby and have my baby home.

Interviewer: Describe the communication with medical personnel after you came back from theatre?

Participant: Ah the communication with medical personnel was still ok because you come with the same people you were with in the theatre upto the time they hand you over to the nurses here (ward) and they read through what is written on your file, they give the necessary requirements, they help you get into bed come check on you when it is time. It was a good reception. So far so good. I can't complain of anything.

Interviewer: What information did they give you after you came back from theatre?

Participant: they were just asking questions how I am feeling and teaching me how I am going to handle myself so when I should stand, when I should drink, when I should eat stuff like that and ah being in that pain and situation how I should handle the baby.

Interviewer: What were some of the reasons for some of the explanations they gave you for instance not eating solids?

Participant: because for me it was the first time to have caesarean section – for not taking solids it has got its own disadvantages and advantages because it is a sore by the time you go to the toilet when you are trying to push you can rip the inside so they prefer you take liquids then at a certain stage they tell you you can now start taking soft things that can help you to go to the toilet.

Interviewer: Was that what they said?

Participant: Yeah picking to what they explained to me. That is what I got.

Interviewer: Earlier you mentioned that emergency caesarean is too sudden for someone to process the information, when do you think is the best time to educate women on the surgical procedure and also about the consent form?

Participant: ah this one can be done like even just to sensitise women out there teach them about caesarean section so that even if they are going in there even if you are told you are going to have an emergency caesarean section at least they have got information they won't be traumatized they won't have a different perception at least they would have learnt one or two things yeah.

Interviewer: When you say teach them about emergency caesarean section, where would these women be accessed?

Participant: ha ha ha women are all over ha ha ha just like the way people sensitise about HIV, the way they are sensitizing about the COVID-19 I think even the caesarean section can be down that way. If they can be sensitized like that women will be ready and they will have information even when they are told you will have a caesarean section due to the child blah blah or any medical condition blah blah then they will be ready for it.

Interviewer: What was your reaction when you were told you are going to have emergency caesarean section?

Participant: Hmm actually my reaction at first I didn't want it I even made it clear I cried most of the time because I had a different perception about it because I had an emergency where they had to save the life of the baby and the baby was not breathing I had to accept it yes.

Interviewer: what are your concluding remarks regarding communication with medical personnel and after emergency caesarean section?

Participant: I can say communication with the medical personnel is fine so far from my experience because they were accommodating, they could listen and teach me and make me ready to understand, they did and they are still doing their job.

Interviewer: Anything else?

Participant: I think medical personnel need to communicate in the best language they can so that the patient is also able to understand that communication system. If they need to use Nyanja they should use Nyanja. If they need to use Bemba let them communicate in Bemba with the patient that they are dealing with right now.

Interviewer: Thank you for your time.

Participant: You are welcome.

<u>PARTICIPANT NUMBER 30</u>

Age: [early 30s]

Marital status: Single

Occupation: Business Lady

Highest level of Education: Grade eight

Religion: Christian

Interviewer: How many children do you have?

Participant: This is my second born child. My first born is 12 years old.

Interviewer: What was the cause of the emergency caesarean section?

Participant: They said the passage (birth canal) was small and the baby was too big. Yes that is what they told me.

Interviewer: Did you come here directly or you were referred from a clinic or district hospital?

Participant: No, I came straight here.

Interviewer: what information were you given before going to theatre?

Participant: They told me sign the consent form and not waste time and that the baby might die from my womb so I refused.

Interviewer: Why did you refuse?

Participant: I was afraid because I have heard the surgical wound is too big.

Interviewer: So what kind of information would have allayed your fears?

Participant: You know people talk to people differently. Others instill fear in you. So they should have explained and talked to me properly and in a respectful manner. They were very different from the people I found in theatre. The people I found in theatre properly explained things to me then I was even able to come to terms with my predicament.

Interviewer: What about the first medical staff you encountered?

Participant: Ah I had refused to have the operation because they didn't explain properly. They were shouting at me.

Interviewer: Ok what information did they give you before they took you into theatre?

Participant: they told me the baby was too big and couldn't be delivered normally and that I shouldn't waste time in signing the consent form for me to go to theatre so I refused. I told them to do the other procedure (induction) that is how they tried the procedure but it failed and that was how they took me to theatre around 04 hours.

Interviewer: So what was written on the consent form, which you signed?

Participant: what was written on the consent form is that I should accept it and when I accept that is when they can conduct the procedure, so they told me sign here and there that was how I signed.

Interviewer: Did they read out the consent form to you?

Participant: yes they did and showed me where I should sign.

Interviewer: so what information were you given after the operation?

Participant: they just brought me here. The day before yesterday they removed the bandage and explained to all women in the ward (at once) that we are supposed to clean our surgical wounds and that if we don't we will get rotten and that this can cause a problem. That is how I understood their explanation and that is how we started cleaning our wounds. They also encouraged us to clean our wounds so that we can heal fast.

Interviewer: What else?

Participant: That is all.

Interviewer: What factors did you consider when signing the consent form?

Participant: Hmm it was very painful kaili they tried both procedures (normal delivery and induction of labour) they failed yeah. That is the reason I signed the form.

Interviewer: Before we end this interview is there anything else you would want to talk about that you haven't mentioned?

Participant: you did well to come because your coming encourages us to fight for our rights. If there are bad things happening here you need to help us so that they are improved.

Interviewer: Thank you for your time.

Participant: I am also grateful.
