# Supplementary material for: Women’s experiences of communication with medical staff before and after emergency caesarean birth in Zambia: A qualitative study
Source: PLoS One. 2026 Apr 9;21(4):e0346694. doi: 10.1371/journal.pone.0346694 (PMC13065054; doi:10.1371/journal.pone.0346694)
Supplement: S6 File — (DOCX) [file pone.0346694.s006.docx]

| **Code** | **Definition** | **Direct quotes** |
| --- | --- | --- |
| Mode of communication | The medium or channel in which information is exchanged between women and healthcare providers, including verbal, non-verbal, and written. | *“The doctor told me I was going to have caesarean section then the nurses who were preparing me for theatre are the ones who gave me the consent form,”*  *“It was the doctor who told me the need for C-section because when I came, I just came for a normal check-up and I was told it is important that I go through the C-section if the normal one doesn’t go through. So it was not planned that this day I would have an operation. So he gave me everything that I needed,”*  *“I encountered a number of them (healthcare providers), it was a chain of them but I think it was the consultant who informed me that I was going to have emergency caesarean section. She was working hand in hand with the midwife. So with the consultant like I said it is something that they do she just said this is a caesarean case which I understood because I am a health practitioner as well but where I had issues is the consenting process. So the one who brought the consent form was the midwife. So you can see the information gaps from the midwife’s point of view but I was made to sign, sign here, do this, do that and because you are running out of time for the baby just sign it, but you just accept the communication gaps and do what they are telling you to do for the sake of the child.”* |
| Emergency caesarean birth communication experience | Participants’ perceptions and evaluations of their interactions with healthcare providers, including both verbal and non-verbal communication, the quality of rapport established, how information was conveyed and understood, and their feelings and satisfaction regarding the overall communication process. | *“I kept on asking questions and you could see they were irritated so even me I equally got irritated the fact that they were irritated but now the focus was okay let me keep quiet it is not about me let me just be hopeful and have this baby just come out.”*    *“You can imagine the damage I would have done to myself. Just that piece of information, which they know and could have communicated,”*  *“There was no communication and the worst part is that you are made to starve, you don’t get to eat anything. It would have been better if they had given me a timeframe to say from this time we won’t be able to attend to you so you can eat something. We will let you know when you can start starving but there was no communication in that regard until my husband became upset and I also became upset then my blood pressure started shooting again, that is when they had to take me into consideration.,”*  *“Ok but when I was taken to theatre I didn’t know they were going to operate on me. I thought they were going to turn the baby and put it into the right position. A lot of people say when the baby is breech, medical staff can turn the baby and put it in the right position. They didn’t tell me I was going to have an operation. I honestly had no idea it was an operation. So when I was in theatre I asked if it was an operation. Then medical staff were surprised. They asked me, you haven’t been told it’s an operation? I responded, no, they didn’t tell me anything. The people in the theatre are the ones who told me that I had been taken there for an operation,”*  *“When I was taken to theatre I thought they were going to perform a BTL, I didn’t know that I was going for caesarean section,”*  *“I was explained to how to clean my wound. They told me to clean the wound three times with lifebuoy soap in a day. They also told me not to eat solids. They also told me to lie down for 8 hours but I had to wake up to take care of my baby who was left unattended to. She was crying. I woke up when it was four hours and I am having a terrible headache because of not following what they told me,”*  *“I know it was an emergency but I think the health professionals or clinicians were supposed to even read it (consent form) or summarize it but it is just where they give you, you need to sign this, it is an emergency. There was more of fear instilled in me. I am making a decision based on fear and not based on information. They came out like you are going to die or the baby is going to die if you don’t do this. So I was in panic mode because the information was more like an alarm and so when the forms came I just signed, they just said sign here without reading through and because I am a health professional, it felt so wrong because whatever decision you make in a rush without really having to think about it, it shows that there is a problem somewhere,”*  *“Ah ok it (*communication*) wasn’t good because some of them were shouting at me over things that I did not even understand. There were some things that I did not understand but I was surprised that I was shouted at. Some of us don’t understand medical issues because it is not in our line of work. So it is better they explain instead of shouting. Like in my case this was my first emergency caesarean section but they shouted at me on things that I didn’t even know or understand.* *But some of the medical staff were understanding.”*  *“At least they should have explained how things were, the process of the procedure but they just came and said sign the consent form, you are going into theatre and told me the reason for emergency caesarean section that the child was overweight,”*  *“I think when it comes to consenting you have to make somebody understand what they are getting themselves into and you can even mention the risks which are there how they should behave, you can mention the odds of a successful emergency caesarean section because I didn’t even know anything about the procedure, how much risk do I have, is it possible that it might not be successful, I didn’t even have that information you know so if I who is a health professional don’t know what more somebody for instance these guys from rural areas who have never even gone to school? ”*    *“I think on my part it was just the pain I felt after the operation, I know people usually say it is painful but I wasn’t told exactly what happens because I didn’t understand why I was feeling very cold but later on after I asked they told me it was the medication that it would clear afterwards but with me I felt hmm this was not normal am I going to be ok because when I came (to the ward) I told mum to put blankets and close the windows but nothing helped not until after everything. I think if someone had told me what to expect after caesarean section that would have helped,”*  *“It is important that medical personnel bring out the expectations, bring out the side effects, the advantages and disadvantages so that even when you go there (theatre), you have an informed decision and your mind is ready for it so that you are not surprised because like when I came out I was literally shivering, I wasn’t feeling cold but I was having this ah involuntary kind of shivering and then I was subjected to that eight hours, it felt like torture. But if they had told me that after the operation this is how you are going to feel my mind was going to be ready for it. So communication is very vital and medical personnel shouldn’t take it lightly especially when we have women that are not educated or that are not literate so to say they don’t understand much of the things. So it shouldn’t be left to the patient to figure out, it is important that they communicate and not just saying we have to save the baby,”*  *I feel the counselling is not there. I wish they had counselled and encouraged me after they removed my uterus. Actually no advice was given, the only thing I was told was that the uterus has been removed and that I won’t be able to conceive again, it was the only thing I was told. So I said ok it’s fine, from there no one has talked to me or has given me any advice on what to do yeah, on how to go about it, no one,”*  *“They explained to me what I went through before they conducted a caesarean section on me. I was told what I would experience, I was told the pain would be there for about two weeks,”*  *“Like feeling numb they had told me that I would feel numb and be able to watch the procedure as it happened,”*  *“At least the doctor who was attending to me, the one who assured me she was quite ok we really communicated well and she really acted professionally.”*  *“On the communication part they did a good job even the time they were carrying me they encouraged me, they told me to have hope and that I would be fine.”*  *“In my case I was in pain so I didn’t want to ask questions for fear of upsetting them because I was the one who was in need.”*  *“The communication was really good because ah from word go they had explained to me what the options were and why we had to go that way. So I think the communication was quite good.”*  *“They didn’t really like take me through, maybe counsel me I wasn’t really like counselled because maybe under normal circumstances they would have taken me through the process this is what is going to happen and this is what you should expect and maybe we expect negativity in this way and positivity in this way. There was no time for that I was just prepared and taken. So I just had excitement to see the baby but I didn’t have information on the procedure and what to expect or what I will go through whilst in the theatre.”*  *“Ah I think when I was told to say you are going to theatre I knew it was an emergency and I expected them to take me right there and then but I remained in the ward up until 7 in the morning, I went for my caesarean at 7 hrs.”*  *“Like feeling numb they had told me that I would feel numb and be able to watch the procedure as it happened.”*  *“There are things that we find out on our own reading or through someone asking questions.”* |
| Anxiety | Feeling worried or unease about emergency caesarean birth. | *“I was already mentally challenged thinking that the worst will come to the worst with my baby.”*  *“I was in panic mode because the information was more like an alarm.”*  *“I was anxious and it’s like I was told around 02 hours that I needed to go into theatre. I am told there was an emergency that came like I was left there for some time waiting when all I wanted was to go into theatre.”*  *“I was anxious because whereby you see people who have just been admitted being taken to theatre. So I couldn’t figure out what was happening I think that is what made my blood pressure to start rising why aren’t these people attending to me as soon as possible.”*  *“I was really anxious to really get there (theatre) and them (healthcare providers) give me that injection so that I feel better.”* |
| Fear | A woman’s emotional response to emergency caesarean birth. | *“It is terrifying because you are asking yourself am I going to come back alive, is my baby going to be ok or are we both going to be ok, so it is kind of traumatic and terrifying at the same time. At least maybe if the mind is prepared it would help.”*  *“I felt like I was going to die.”*  *“I was scared, I felt like this is the end you know mmm ah I was very scared I can’t lie I was very scared. I just thank God that I am still alive today. It is just by God’s grace. I had lost hope but I thank God that everything worked out for good,”*  *“There was more of fear instilled in you. You are making a decision based on fear and not based on information really so yeah.* Y*ou even know that if anything was to go wrong they wouldn’t even be accountable because apparently that is the system in place. So yeah it was scary, very scary.”*  *“I was scared ha ha because it is my first time. So I was very scared ha ha.”*  *“Hmm I was afraid of the operation and just having a wound.”* |
| Frustration | An emotional response arising from communication experience and unmet expectations (feelings of irritations and annoyance). | *“I kept on waiting and waiting from 18 hours up to 12 hours the following day that is when I was taken into theatre whereby they knew that my case was also an emergency but they didn’t treat it as such but anything can happen at any moment looking at the high blood pressure I had then my baby would have gone into distress it can affect her and also me.”* |
| Helplessness | Feelings of lack of control and powerlessness. | *“It’s just like you are a rat or a rubber, they just throw you here and there.* *You are just at the mercy of healthcare providers, you are just being experimented on you know, had I been on the upper hand it is not something I would really advocate for,”*  *“Sometimes they are talking to one another but not talking to you it makes you feel more of an object, you know objectifying you, when they are supposed to explain to me.”* |
| Worthlessness | Feelings of unimportant, meaningless and inadequacy arising from perceived neglect or lack of support. | *“I felt neglected, like my case was not important because I was also fighting for my life whereby they are just quiet, it made things worse,”*  *“Ah I felt like I was just an object ha ha like I was just an object on a bed, like I had no feelings, yeah like an object like I had no feelings whatsoever because this was my life they were talking about and I wasn’t even involved in the process of delivery or being communicated to. So I just felt like an object, nothing much, just like an object. It’s more of mechanical, like lie down, do this, and do that not really being reassured that you will be fine,”* |
| Nervousness | Emotional response of unease, apprehension as well as restlessness. | *“I think to ease my nerves a little bit, they should have explained that this is what is happening and we have to do this to save your life and the baby’s life and the procedure will be just like this.* *Even just a few sentences just to put me at ease but that wasn’t done,”*  *“I was nervous, yes I was very nervous because it was my first time.”* |
| Surprise | Emotional response to unplanned caesarean birth. | *“I expected something less stressful because I was even surprised by how long it took, they didn’t even tell me how long the procedure is.”*  *“To me that one (emergency caesarean section) was news because it was my first time. I know there is caesarean section but I didn’t know there is emergency. Ah something which comes as an emergency it is always shocking but when you have heard about it you are aware you know something about it. It helps.”*  *“I didn’t expect that I would be giving birth this week I was three weeks away to the due date. I was in shock really. I had to take time to process everything I think by Monday that is when I accepted I said whatever happens, happens as long as it is for the good of the baby and me.”*  *“It is too sudden to process everything when you are told you are going for an operation.”*  *“It was just there and then and I had to go although it is not easy for somebody to accept in the shortest possible time. So in terms of communication you really need somebody who is fast in thinking otherwise if you say let me think somebody about to go in theatre they prepare their minds mentally and physically but this one hmm it was just there and then.”* |
| Coping | Strategies participants used to handle stress | *“I felt sad and prayed to God because most people don’t survive the caesarean section especially when they sign the paper (consent form). Before you have an operation you have to sign a consent form so that if anything happens to you, the doctors are not held responsible. You just have to rely on faith to survive.”*  *“I felt very sad and bad because my first child was delivered through an emergency caesarean section. I have four children. The second and third were normal deliveries. The fourth one was an operation again. Anyway, I just prayed to God.”*  *“I wasn’t afraid because I put everything into God’s hands.* *I was scared at the beginning but with God on my side I eventually calmed down. Having faith in God calms me down because he makes a way where there is no way. God has the final say. God guided me on what to follow and accept caesarean section.”*  *“It is by the grace of God that they chose this path for me, I did not chose it for myself.”*  *“Because of the experiences I have had, I just did research on my own like I would read this I would read that and what I found out was when you are having a normal natural birth somehow the baby develop stress like they are distressed. So with me I considered caesarean to be faster than the normal birth where you go through the process dilate all that.”*  *“It feels bad but what can I do? Nothing, it feels bad but there is nothing I can do since there is no one to advise me so I just comfort myself ha ha.”*  *“It is not easy but with hope and prayer of course I would like to say I was praying at that time but if you have faith and pray about it, it goes well.”* |
| Communication barrier | Any factor that hinders effective communication between women who underwent emergency caesarean birth and healthcare providers. | *“In my case I was in pain so I didn’t want to ask questions for fear of upsetting them because I was the one who was in need,”*  *“I was in pain, I didn’t know anything when I went in theatre but I just signed because I really needed help. I didn’t even read the form(consent form),”*  *“I was afraid because I have heard the caesarean section wound is too big. They didn’t explain properly. They were shouting at me. You know people talk to people differently. Others instil fear in you. So they should have explained and talked to me properly and in a respectful manner. They were very different from the people I found in theatre. The people I found in theatre properly explained things to me then I was even able to come to terms with my predicament,”*  *“I couldn’t ask any questions because of the way they handled me, it really looked like an emergency,”*  *“I would rather they explain that when you do this and that it will affect you this way than some other nurses come out to say I am not the one who impregnated you, such words are not good. So I would really love to see a change on that,”*  *“For this hospital they use a lot of Bemba and Nyanja and we see a lot of people in here, we are not all Bembas and Nyanjas yes. Maybe if there could be a diversity of language so that medical staff can give information that everybody requires regardless of the language the patients speak,”*  “*I told them (medical personnel) I will continue bearing children as long as God gives me. I don’t know if they wanted to tell me that they wanted to turn the baby since it was breech but they didn’t maybe because I told them I didn’t want to have my tubes tied,”*  *“I never looked forward to having my baby through caesarean section because I believe that it is a sore that doesn’t heal for a very long time until you die,”*  *“The communication wasn’t good because some of them were shouting at me over things that I did not even understand. There were some things that I did not understand but I was surprised that I was shouted at. Some of us don’t understand medical issues because it is not in our line of work. So it is better they explain instead of shouting. Like in my case this was my first emergency caesarean section but they shouted at me on things that I didn’t even know or understand. But some of the medical staff were understanding,”*  *“They should treat patients in a respectful manner and not be rude. They should understand each patient and talk to them in a polite way because a patient is in pain and their attitude can worsen the condition. You know each person is different and we react differently. Sometimes they are rude but they need to understand the patient and talk to them nicely so that they understand and that way there will be proper communication,”*  *“They treated me well and spoke to me nicely as a patient is supposed to be handled,”*  *“Actually the sister (midwife) didn’t have that time to explain because even her she was told by the doctor that let us do a caesarean section on her so even her she was busy rushing to do this and that because I had no catheter they had to put what is this, a cannula on me so she was alone in the labour ward with me so she didn’t have that time to explain what was written on the paper (consent form). So she just said ok sign here to show that it was your decision and willingly you have to do this. So I got the pen and signed. Then she started preparing me to go to theatre,”*  *“It would be nice if it (information)was more elaborate, a bit in detail even told the risks of not doing certain things they tell you to do but it is just listed one, two three no explanation. Maybe if you ask but you also have to ask in a certain way otherwise you will be shouted at and seen as if you are wasting their time.”*  *“They should treat patients in a respectful manner and not be rude. They should understand each patient and talk to them in a polite way because a patient is in pain and their attitude can worsen the condition. You know each person is different and we react differently.”*  *“You don’t have time to ask questions because it is an emergency so you might even miss out important questions.”*  *“I don’t know if they wanted to tell me that they wanted to turn the baby since it was breech but they didn’t maybe because I told them I didn’t want to have my tubes tied.”*  *“I think medical personnel need to communicate in the best language they can so that the patient is also able to understand that communication system. If they need to use Nyanja they should use Nyanja. If they need to use Bemba let them communicate in Bemba with the patient that they are dealing with right now.”* |
