# Supplementary material for: Women’s experiences of communication with medical staff before and after emergency caesarean birth in Zambia: A qualitative study
Source: PLoS One. 2026 Apr 9;21(4):e0346694. doi: 10.1371/journal.pone.0346694 (PMC13065054; doi:10.1371/journal.pone.0346694)
Supplement: S7 File — (PDF) [file pone.0346694.s007.pdf]

<u>DISCUSSIONS WITH HEALTH CARE PROVIDERS</u> (5 Consultant Obstetricians, 2 Key Informants, 5 Midwives, and 5 Registrars)

CONVERSATIONS WITH CONSULTANT OBSTETRICIANS

PARTICIPANT NUMBER ONE (CO1)

Interviewer: Doctor, welcome to this interview and thank you for agreeing to speak with me.

Participant: Thank you [name of interviewer removed]. May I congratulate you for undertaking this important research?

Interviewer: Thank you for your kind words doctor. Who attends to women who need emergency caesarean section?

Participant: Right, so at [hospital name removed], for women who are just referred coming into the hospital, the Registrar is usually the first contact. These are post graduate students who tend to specialize in obstetrics and gynaecology. So why do we assign a Registrar because these are referrals so they are coming into a tertiary hospital where doctors are trained even as specialists. So we make sure that a higher level cadre of health workers see the women so that decisions are made instantly if emergency caesarean section is to be taken, there should be no delay so that the mothers and newborn's lives are saved. So the first contact is the Registrar.

Interviewer: The other day I contacted you over the phone you were talking about [clinical teams], what are these and what are they all about? Could you please elaborate more on this?

Participant: A [clinical team] is a unit, it is for administrative purposes to manage patients. So it is headed by a Consultant. In [hospital X], there are [clinical teams, which rotate according to a weekly schedule]. So, the number of doctors in the [clinical team] varies depending on the intake of post graduates. If that year we have enrolled more post graduates, the Registrars then the [clinical team] has more numbers of doctors but as a rule of thumb a Consultant heads the [team]. There could be one or two Consultants followed by a Senior Registrar who is a Post graduate who has already obtained the qualification, they are still being under studied before they can become Consultants. And these we have the Registrars, these are doctors in training, their training is [text removed]. So each [clinical team] has first year post graduate, second, third and the senior is in [removed] year who reports directly to a Senior Registrar who just qualified also and has his

Masters’ degree and also reports to the Consultant. Then we have resident, these are junior resident medical officers, they are interns, these are new graduates in the medical training. So they are doing their rotations. So they are working under supervision and also among the people the Registrars work with them, so they are on the ground. They tend also to be the first contact but in a [removed] we ensure that the Registrars see together with the intern because the intern may not make an urgent decision. So we make sure that the Registrar is the one who sees a woman coming in for a potential emergency caesarean section.

Interviewer: Where do the mid wives fit in the [clinical teams]?

Participant: Mid wives as you know especially in obstetrics and gynae its team work. The mid wife is what we call the backbone of maternal care. So they do the initial assessment in terms of vitals, checking the heartbeat of a baby, she records her own findings and informs the Registrar about her findings. It is not far - fetched to say that the trainer of any obstetrician has been moulded by a midwife because these are the people who are the backbone, they see and do initial assessment on the patient, they can alert you because there are colour codes, red means urgent, they will just say doctor can you see this mother immediately. Needless to say even those supposedly straight forward cases the midwife always just has a look in case I miss something. So they are the ones who are a backbone and they alert the obstetrician if there is a problem.

Interviewer: Please explain to me what happens when a mother walks in or is brought to [hospital X] up to theatre and after the operation until they are discharged.

Participant: [sentence removed]. So we get referrals, there is a communication casket. The hospital is informed for example the referral centre says this mother is bleeding in labour, so the hospital is alerted and then we offer the initial management we advise for example if the baby has what we call fetal distress we advise them can you make the mother lay on the left side, give oxygen, <u>radiate</u> that mother before you put her in the ambulance. So we give pre referral advice. The mother is usually accompanied by a midwife, at the receiving bay which is in [name of place removed] labour, the midwife who brings the patient hands over to the fellow midwives, they give a summary, this woman has been in labour for how long, how far is the dilation, what is the fetal heart rate and then the receiving midwife together with the one who has brought the patient reexamines and verifies those findings and together they check the vitals bp, pause, check fetal heart again and record because there could be no fetal heart at arrival so the midwife who is

receiving needs to verify that indeed this baby is alive at [hospital X]. So once they agree on those findings the midwife then calls the Registrar, the Registrar then verifies oh yes there is a distress, fetal heart is 180 for example, mothers vitals bp is normal what what what. Immediately the Registrar makes a decision and orders an emergency caeser, these other measures have already been started oxygen, iv fluids and left lateral laying on the left side all that, there is enough blood returning to the mother's heart. So the Registrar then activates caesarean section call to theatre. Theatre then activates its system which includes the Anaesthetist, the porters to pick the patient. The Registrar in labour ward who has diagnosed the need for caeser communicates with the colleagues in theatre, one doctor is usually stationed in theatre, we have fetal distress at 36 weeks mother's vitals, gives a very quick summary of the assessment. The Registrar there together with the theatre staff prepare, the idea is to have limited or little time to decision to incision time because the longer the decision is made to actually conduct the operation, the worse the outcome. So we always note what time was the decision to do caeser made and what time was caeser done**,** that was one of the research one of the doctors did and then the patient is taken from labour ward, again the hand over goes into theatre, the labour ward nurse, midwife does the handover to the theatre nurse, what is the vitals, that nurse also verifies oh there is a fetal heart, the Registrar also verifies who is in theatre because it would be a very big disservice where you don't listen you don't reexamine and lets cut and remove a dead baby or you start cutting the baby is already coming out. That is why that reexamination even in theatre is advised. So the caeser is done, the midwife who brought the mother remains in theatre because she has to receive the baby, she has to go back. She has to go in theatre receives the baby hands over the baby to the surgical team tells them you have extracted a male or female birth weight, what is the apgar score, that is the score we assign at birth, one minute and …….. and then the surgeons remain to finish because the mother actually the reason why the operation is done it has to be closed monitored after surgery, initially we monitor them in what is called a recovery room within theatre where the vitals are monitored frequently we have to make sure there is no bleeding that mother is recovered. When they are fully conscious that is when they are transferred to the ward again the handover continues, theatre to the postnatal ward where she is going, vitals are given inter checked so that the one taking her indeed verifies I have received a mother whose bp was normal, pause was normal because they can refuse to get that mother if they discover bp is not recorded. So it is a very tight procedure at any level, the idea is to ensure safety of the mother and the baby at all levels. It's quite tight so no where can you

receive a mother who is already in shock without questioning the one who has brought her, but what have you done, you are handing me over a patient whose bp has dropped what have you done? You haven't put a cannula so the process ensures that the mother's life is protected and the baby's. Even when they go back to ward we advise monitoring for 24 hours in an area where I am sure in the wards there they showed you where it's called acute bay or they reserve a room specific for post caeser. Why should they have their own room because we want to make sure this woman doesn't bleed they have no blood coming out of their genitals or the wound site, the vitals are maintained within normal. The baby and the mother are fine. That is why the first 24 hours is critical. So at each stage the vitals are monitored and the next team that takes over if it is theatre to postnatal they make sure after surgery that patient is stable.

Interviewer: What is the role of the Consultant in obstetrics?

Participant: the role of the Consultant is like the overall supervisor, so the Registrar I for one I am a consultant I am kept informed of whatever case is going on and I can even advise no this case not your level, this one requires a senior Registrar or consultant or someone who has finished their specialist training for example someone with four previous caeser and then a first year wants to go in I say no no this is a difficult case because of adhesions you may injure a bowel or bladder don't do it. Give a senior Registrar. So the Consultant is like the overall, we make sure there is quality care that is the aim, quality care for our mothers and the newborn.

Interviewer: Are there times when the consultant has direct contact with the patient? I mean do you speak with the woman directly when they are scheduled for an emergency caesarean section?

Participant: The ones who talk to them (women) are the midwives and Registrars. There are only few cases where we are called for example someone who can't consent under 16 years or imbecile or unconscious patient without relatives then we have to take responsibility. They are the ones where we consent.

Interviewer: How would you describe communication between healthcare providers and the women who undergo emergency caesarean section?

Participant: what I can say is being an emergency nature operation, I would say that the counselling could be perceived not adequate because of the emergency nature. So you have more time to save that woman so it is not like you are going to spend a lot of time explaining why

someone is bleeding so you expect the woman to understand her condition because she is seeing the blood and you just say mum you are bleeding, your life is at stake your baby is at stake or someone maybe has distressed baby, so you say we are trying to save your baby's life. So in short the counselling is quite specific and relevant, very specific and relevant.

Interviewer: What kind of information are women given before they undergo emergency caesarean section?

Participant: they tell them the indication for emergency caesarean section, that one is very important you will be driven to theatre, she has to understand and her risk if she doesn't take that operation. In terms of explaining the risk of surgery and anaesthesia I think that one is never given, I can't say it is never given but what is of important is to understand why they are being taken to theatre, explaining the risks like you may die on the table, it usually comes secondary because at that moment your aim is to save life of the mother and the newborn baby. So critical information is explaining why you going to theatre, mum you are bleeding, mum your baby is not breathing well, mum you have fitted so there will be a risk of another seizure if we don't take you to theatre. So the most important thing is explaining why we are taking this woman to theatre and the consequences of not going there. Those are very critical.

Interviewer: please describe the process of consenting to emergency caesarean section?

Participant: so what it is the process is once an indication is explained, you explain to them this is the reason we are taking you to caeser you are bleeding, the baby is still alive so we want to save the baby and yourself. Once you explain that then you call the midwife, the midwives help, and they are always there with the doctor. After explaining the indication, the consent form, I think you saw the consent form which elaborates that I have undertaken this operation it will not be necessarily be done by a doctor of my choice that is again to cover whoever again is going to do the surgery and understands the risks which mostly anesthesia, the bleeding and so forth. So we run through those but the most important is primary understanding of why they are going to theatre. So after the woman is explained they consent. There are things we avoid asking them to consent like BTL because they may be under duress to sign to stop giving birth. So that is why if we have issues with ligating the tubes, BTL we are supposed to do it at the same time to say in case you undergo caeser can we also do BTL. For example someone with four previous caeser we are supposed to already get consent for BTL before the emergency.

Interviewer: So who gives this consent form to the woman?

Participant: the midwife, so the midwife will explain having examined that patient with the doctor she knows the indication, she has explained to the midwife, the midwife understands then they explain to the women. So the midwife like I said is the backbone of maternal health. So they are the ones who hold the consent form and explain mayo muleya ku (mum you are going to) theatre and then explains why and being told that don't have a choice of a doctor to perform the surgery and then on the consent there are also risks, they have explained to me and I have understood and sign.

Interviewer: please explain to me the communication between health care providers and women after surgery?

Participant: What happens now we are using spinal anaesthesia, which allows a woman to be awake so we just numb them unless there are contra indications to spinal anaesthesia. So you numb them, the communication doesn't just end at consent during the surgery the anaesthetist keeps talking to the woman, explaining to her how she is feeling, are you having difficulties now they have removed the baby and the baby is shown to the woman at delivery. That is the advantage of spinal anaesthesia. So they see the baby, the baby is breathing well, you have seen the baby everything, we are taking it to the ward you will find the baby. The communication is like throughout. After the operation she is explained to how she takes care of spinal anaesthesia because the complaint is the headache, they are told to lay flat, they are also told to take lots of fluids and then they are explained also when to start eating. So the goodness of spinal you continue counselling them even what you expect after surgery they are explained to, you will still feel numb but six hours later you will be able to stand and use your limbs. How do you take care of the wound, this wound will be exposed after two days, wash the wound with lifebuoy soap after apply spirit ….you know the counselling is ongoing. Even the ones under general anaesthesia will sleep soon after they are fully conscious we advise them now you can start liquids then later light meals mobilise and come out of bed. We even encourage them to chew chewing gum and how to look after the wound we explain. When to take their first bath and so forth. So immediately if it is under general anaesthesia they are fully awake they are explained to. Sometimes we even explain it if we have enough time before surgery what to expect after the surgery. But each day more information is being given, mum this is now six hours after the operation you can start taking

fluids, 8 to 12 hours later they can even start to eat light meals especially with spinal anaesthesia. They ambulate fast short time they are out of bed that one also helps to recovery.

Interviewer: Earlier, you mentioned that you only explain the indication for emergency caesarean section and the consequences of not consenting to the surgical procedure, why is that?

Participant: some women refuse for example someone has fitted they say no I don't want the operation my baby is still small but you know eclampsia or any hypertension is a progressive disease as long as she has not delivered the fit may occur and the next fit may cause a stroke so you have to explain. So the idea is conditions where they may feel it is not necessary to undergo any emergency caesarean section.

Interviewer: Sorry what I meant was why is it that you only explain two things – indication for caeser and consequences.

Participant: In an emergency you have very little time to start talking about risks. Long pause follows – I think you and I have boarded a plane, if the airline starts concentrating on the risks of air travel someone will turn back at the door of the plane. So you give mostly about the positives of emergency caesarean section. Of course risks of the surgery can be given but it shouldn't outweigh the positives and then you assure the patient that these are trained people, the whole team anaesthetist, the surgeon, theatre nurse, and the midwife so we are trained to have no room for error that is why we don't emphasise on the risks of surgery. So we give mostly the positives.

Interviewer: what are some of the challenges that you face in communicating with the women that undergo emergency caesarean section?

Participant: I think the challenges are those who don't understand their conditions. I think one condition we usually have problems with is eclampsia or severe pre-eclampsia, some women do not understand how severe this condition is, they only look at one side no you can't terminate my pregnancy at this early gestation period of twenty five weeks or twenty six weeks. Then with pre-eclampsia when it's a small pregnancy or severe pre-eclampsia you have to terminate. So those are the challenges we face if the patient does not understand the situation they are in but there are clear situations where there is no problem like bleeding she is seeing the blood and she knows the consequences of not consenting to a caesarean section if she is bleeding. So the challenges are some of the conditions which some patients may not perceive as an emergency, they may not

agree. The other issue is about teenagers I have had challenges with them, convincing them if there is no older relative you just have to sign the consent on their behalf. The hospital takes responsibility. So the under 16s they present a challenge because they may not understand what is going on, they are children. The other challenge, it's a small number, women who have had several deliveries we call them multigrand…more than five to six babies, they feel they can make it (deliver normally) normally even when you tell them no no this time around you can't deliver mum but they will say that I have delivered before. So those also present with some challenges. They feel they have always managed so there is no need to go for caesarean section.

Interviewer: You mentioned that one of the challenges is that women don't understand how severe their condition is, why do you think that is so?

Participant: it is literacy levels, it is all about education. That is how I can sum it up. Yeah education, we have no challenges with somebody who reads on their own, they even tell you doctor I have read about eclampsia just remove the baby. So with the educated we have no problems most times. There are times we talk to the partner who maybe more enlightened to convince the woman. But the final decision is in the hands of the woman as long as she is of consent age. The final decision is the woman.

Interviewer: As we conclude the interview, is there anything of importance that you would like to talk about regarding the communication between healthcare providers and the women who undergo emergency caesarean section?

Participant: my last words, I usually talk to my Registrars no matter how emergency a situation maybe take time to explain what you are doing don't just tell this woman you are going for caeser just sign here. I tell them explain because not all the time can things go right someone may die on the table and the relatives will say this woman was forced to undergo surgery. So I always tell doctors no matter how emergency, one of you can be assigned to talk to the relatives about the condition of the woman so that relatives understand what is going on. Apart from the woman herself she is part of the bigger family you need also talk to the husband and other relatives why you are taking her for caeser. So that is my last message communication, communication, communication counselling very critical and it makes a good doctor a good midwife if you spend time explaining but not really at the expense of saving her life but it is part of good clinical practice, explaining what you are doing and giving the woman if there is any alternative but it is very tricky

to give an alternative when it is an emergency caeser. But just explaining to them what you are doing, I always emphasise to my doctor you won't lose anything explain why you are taking the relative for caeser, they will understand and they will easily consent. So it's very important that at every stage you explain what you are doing because the women are the reason we are working in this facility. So there shouldn't be one way communication, it should be two way, listen to them also their concerns.

Interviewer: Earlier you mentioned that you only inform the women the indication for emergency caesarean section and consequences of not consenting to the surgical procedure due to limited time, what other factors apart from time do you think constrain medical personnel from fully communicating with the women who undergo emergency caesarean section?

Participant: the other reason is the woman who is undergoing an emergency caesarean section might be not in the right state of mind to be fully explained to, she is bleeding, she is restless so what she needs from you is immediate intervention so those are the constraints which stops you from explaining in full because the woman will be bleeding and she is restless and you need to act and if you look at her eyes she is pleading save my life.

Interviewer: Doctor, thank you so much for your time.

Participant: You are welcome [interviewer name removed]. Thank you.

PARTICIPANT NUMBER TWO (CO2)

Interviewer: Doctor, welcome to the interview.

Participant: Thank you.

Interviewer: Please take me through the process when a woman is brought in for emergency caesarean section, what goes on?

Participant: ok yeah so when a woman is brought in for emergency caesarean section either they would have come from home or a general hospital, which is the first level hospitals and would have referred as such for as emergency and so once she comes in then for us who receive will have to assess her meaning just confirm what the problem is, examine, assess and inform her about what our findings are whether she also agrees with what she was told where she is coming from being referred from also inform her if it is an emergency she needs emergency operation and usually it will be either to save her or to

save her baby depending on whatever the problem is. So she needs to understand that and then once she understands she needs to sign the consent to agree to whatever we have explained to her yeah.

Interviewer: Please describe the communication between Consultant Obstetrician and women who require emergency caesarean section before they are taken into theatre.

Participant: Ok the communication, I will just give an example, for instance if it is fetal distress meaning that the baby is distressed or the heart beat is not good if she is not delivered by caesarean she might end up losing the baby. So once she is examined and you confirm that I am not happy with the baby's heart beat maybe she has had prolonged labour ah then she needs to understand what is going on that she has been in labour for some time maybe seems to be tired and we can no longer wait for her to have a normal delivery and if we wait too long we may end up losing this baby so we need to take you for an emergency caesarean section to save the life of the baby and the type of anaesthesia normally the one we use is spinal anaesthesia which she doesn't need to sleep but in case of any eventualities we may have to put her to sleep. So she needs to understand that she maybe awake during the procedure and the procedure will take about thirty minutes or an hour. So then she knows that the procedure will be done. Of course sometimes there are complications of even the operation of the anaesthesia that might happen but most times you don't want to emphasize on that because it scares the woman. You may say ok you may end up bleeding too much so you don't want to scare the woman you want to reassure her about the procedure and the outcome as well yeah.

Interviewer: What is the standard of communication in this hospital?

Participant: the standard of communication is verbally like one on one you explain to them I don't know maybe you can elaborate when you say the standard of communication.

Interviewer: What is expected of you to communicate to an emergency caesarean section patient?

Participant: Most important is the reason why they have to go through that emergency operation and most times they want to know how long it will take and will I be ok, so the outcome most times they will need to know how long it will take and of course they will ask is everything going to be ok, so is to reassure them and also they need to know that

they might be any complication. For instance, you start off by saying you will not sleep but you find that due to whatever reason to achieve your local anaesthesia you have to end up making her sleep and other complications for instance you start by saying we are going to do just a simple caesarean section and then if she bleeds too much during that operation and to save her you need to end up removing the uterus, those are complications that can happen of course it is not every day once in a while it can happen and while she needs to know you find that you do not have time to explain to her, she just needs to know the procedure this has happened we need to proceed this way.

Interviewer: So at what point do you communicate if for instance her uterus has been removed?

Participant: That will be communicated after the operation has been done, you need of course to allow her to wake up, to fully be awake because by the time you end up with a complication it means you would have converted her to general anaesthesia where she has to sleep. So she has to be fully awake and that can take some hours it may even take 12 hours later sometimes depending on the condition even the following day we don't have to communicate like immediately she is waking up boom ha ha ha what has happened so she needs to really be fully aware of the surroundings that she is fully conscious like 24 hours later then we communicate this is what transpired this is what we needed to do so the implications for instance the uterus these are the implications you will not be pregnant again, the implications of whatever has been done has to be conveyed.

Interviewer: Since it is an emergency, how much time is required to communicate to a woman?

Participant: It is just a few minutes normally within five minutes you would have actually communicated, explained and she should be able to understand during that time. Most of them will understand, a few may not understand or they may need more time to think about it or they may need to consult usually when they want to consult it is either their husband or their mother which we do allow them to do that especially when they have a phone with them it easy for them to call. If they don't have talk time you just have to offer your talk time and say you can use my phone to communicate so that either the mother or husband are aware that this is what is going on, it is an emergency they didn't expect it then suddenly she has to go to theatre. The husband also needs to know. So that usually can take even up

to thirty minutes but it is an emergency. So a quick decision has to be made as soon as possible. Very few still insist that no give me time and there is no way I can force her, she needs to be given that time but she needs to understand what it entails the long we wait what it entails if she is bleeding she needs to fully understand.

Interviewer: Please describe the process of consenting to emergency caesarean section.

Participant: Normally like after you give the information, you can see that she is fully conscious and understood then we give her that form. I don't have one here (starts searching for a copy of the consent form on her desk).

Interviewer: I have a copy of the consent form

Participant: Oh you have one, so the consent form is where they need to sign that they have agreed to the operation and then there is also a witness to it. Basically it is for them to say I have been explained to and I understand.

Interviewer: Is the removal of the uterus included on the form?

Participant: No, no so those are complications which are not really indicated on the form that this can happen because it can be anything, those are not indicated specifically. So if it happens then it is communicated later on yeah so unfortunately they go in (theatre) without being told that if complications arise there are possibilities of removing the uterus.

Interviewer: What are some of the challenges that you face with communicating with women who undergo emergency caesarean section?

Participant: Some of the challenges are making them really understand yeah so I think some could have been in pain so whatever you want to do she says do whatever you need to do to help me and because of pain they want you to go ahead with whatever, there will be those ones who will be like that others you will explain and you know that they have understood we don't have any other way I understand, there are few who would actually say I need to wait for so and so to come so that is challenge because you are hoping that this person can understand and quickly make up a decision especially if it is an adult but you do understand that our communities usually an individual may feel like I don't want to make this decision alone, this happens people have to know but that process of waiting someone may take a while and that might be a challenge. So I say do understand that this may happen if we continue to wait so she says oh yes but I really have to wait for someone. Sometimes we don't face much of a challenge if someone is bleeding because they are

seeing that they are bleeding it doesn't take much to convenience. But here comes a woman with very high blood pressure that ok they have all these signs, they are vomiting the next thing they will be fitting so you don't want to wait for too long but the woman may not realise because they may not see it, yes I was told my blood pressure is high, yes I am vomiting but other than that so they may want to wait for someone to come they discuss and yeah you may find that you don't have much time because it is an emergency you don't want to wait for too long as a doctor.

Interviewer: What information are women given when they come back from theatre?

Participant: once they come back from theatre depending on the outcome really and most of the times the outcome for emergency caesarean section is for both the mother and baby to be ok. So if the baby is ok everyone is happy everyone is congratulating them that is what they want to see and them also being ok**.** I think we don't give much information other than ok everything went well we are happy and yeah if unfortunately the outcome of the baby was not good we have to find a way to communicate because you know that already this is stressful and as you communicate you don't what them to feel bad of course it is bad especially if the baby didn't survive yeah and worse still if you end up removing someone's uterus. That has to be communicated but you have a way of communicating it is not just like oh by the way this happened and we removed your uterus you know because it is a human being and there are a lot of expectations for a woman even to have another child so we have to gently rely the information.

Interviewer: Who is responsible for obtaining consent for emergency caesarean section?

Participant: Ah normally mmm I would say we end up leaving it for the attending Nurse/Midwife to actually to ensure that that is signed. For some reason ha ha ha ah but that is how we have done it. So you explain and once you explain then you actually say sister, this is the situation we are taking her to theatre ensure that the consent form is signed. So at that time you would have done the explanation and hand it over to my midwife like let her sign the consent, she will be the witness.

Interviewer: Is that the correct procedure?

Participant: Ah I think it is both as a doctor also the midwife yes because she is part of the team but if the doctor is going to be leading that team I feel that the doctor must ensure that he or she sees what has been signed for yeah. I think we just tend to use a lot of short

cuts and be quick when you have explained she has understood maybe you are attending to another person then you involve the nurse to take over that. It is like that has gone on and on and yeah such that it looks like it is right but when you really think about it really I should be the person to ensure that even what is signed for this is what the woman has actually signed for yeah.

Interviewer: How does the Consultant Obstetrician ensure that the midwife obtains informed consent?

Participant: I think we do the explanation and we ensure that ok this woman is understanding ah and so normally when they sign they would sign for caesarean section because that is the procedure you are going to do and so I am assuming I have explained to her and she has understood and then she will sign for the same procedure I said I will do yeah.

Interviewer: You mentioned that time is limited for an emergency caesarean section and that you have a workload, when do you think would be the best time to communicate with women who undergo emergency caesarean section?

Participant: Just as the word says it is an emergency, so at the moment you have realised here is the problem, that time that communication needs to be done because you don’t have time to wait so the moment you make a diagnosis you say ok this is the situation I think we cannot continue waiting you communicate. I feel at that time is the right time though sometimes it may just come suddenly to a woman like oh really sure, the ones who have been labouring for some time and have failed to progress can understand to say ok i have been in labour for so long and I think hmm if things are not working well sometimes they cry for the caesarean section themselves but so for someone ok suddenly you feel the baby's heart beat is not good you don’t have much time to wait and tell her even an hour later. She has to be communicated to immediately, those are the ones you find that she is suddenly taken aback because things were going well but suddenly you are telling her oh there is a problem but they need to understand this is the reason you come to deliver in the hospital that we need to be monitoring you at any time things can change we will be able to detect that there is a problem and as such things can change when you think it will be a normal delivery suddenly we are telling you oh

no we need to rush you into theatre. I think the best time to communicate to these women is when you have made the diagnosis and tell them you don't have much time to wait.

Interviewer: What is your assessment of antenatal services countrywide?

Participant: I don't think most of them are prepared mentally, the few that are mentally prepared are the ones that you are able to detect as they come for antenatal for instance if she has had two caesarean sections before definitely you know that she is not going to have a normal delivery so the moment they start their antenatal we explain to them to say ok because of abcd we are not going to allow you to have a normal delivery and as such as we get close when you reach 38 or 39 weeks we shall do an elective operation, we will not allow you to go in labour unless if labour starts early yeah and maybe there could be other problems where you feel this one will deliver via caesarean section. So those ones are prepared as they come for antenatal. The other clients whom everything appear normal probably may not be prepared because even when they come for antenatal we do not normally emphasise the fact that when this occurs we may end up doing emergency caesarean section I dont know whether it would be right to do that, my thinking is sometimes you end up scaring people away hmm kuja bafuna bazichita chabe ma (there they just want to be conducting surgeries) operation and sometimes you do get to hear women say ah they just want to be doing operations. So if there is nothing to indicate that this one will end up doing an operation if everything appears she may have a normal delivery you tend not to talk about the operation although it is something that can just happen as an emergency.

Interviewer: As we conclude this interview is there anything of importance that you would like to talk about regarding communication between medical personnel and women who undergo emergency caesarean section?

Participant: What I would say is I think it is just for us to put it in such a way that the women understand ah because it shouldn't be like something you are forcing you know that this operation must be done to save a woman or to save the baby but it shouldn't be like a forcing matter ah what is most important is for the woman herself to understand and even when antenatally she is set out knowing that I will have a normal delivery but emergencies can happen. So I think in the communication what is vital is just that understanding making sure that the message is relied properly so that the other party also understands yeah which is the

indication and the consequences of if I don't do the caesarean section do I have an option because if there is an option probably it will not be an emergency most times when it is an emergency it means that is the best way to save the two lives or one of the lives. So the important thing is for the woman to understand the reason for the emergency caesarean section and the consequences.

Interviewer: Doctor, I would like to sincerely thank you for your time.

Participant: You are welcome.

PARTICIPANT NUMBER THREE (CO3)

Interviewer: doctor, welcome to the interview.

Participant: thank you very much.

Interviewer: please take me through the process of when a women is brought for an emergency operation.

Participant: they come straight to the labour ward so there, there is always a team of nurses to receive the woman. They will do the vitals that is checking of the bp, the pulse, the temperature, they will check the urine, they will check the weight of the woman and also just a quick assessment by the midwives in terms of why has the woman come to labour ward and then being referred or have they just come for delivery services or you know ah so the background is very important and then after that there is a quick assessment just to know how the woman is doing breathing you know how the baby is by listening to the baby's heart and then also to check if it is labour at what stage labour you know are we at. It is a quick process by the way that same room called the admission room in the labour ward there are nurses and the doctors. So the nurses just do a quick summary to prepare the doctors to see the patient and after the nurses quick review the doctors come to get the detailed history. The importance of that history it will inform you what you are dealing with for example, what number of pregnancy is this, what previous circumstances of pregnancy that may affect this delivery for example have you had caesarean sections before, have you had any operation before like for fibroids, how has been your antenatal care where were you receiving it from what were the discussions during antenatal in terms of your delivery plan and so on and so on. Are there any

medical conditions that can affect this pregnancy in the background it could be diabetes it could be sickle cell ah what about the growth of the baby is it ok, is it one baby or two or three babies, how is the baby laying you understand all that is quickly checked through the antenatal like to give a summary of the history. After that the doctor now exams to see are there any danger signs that to make it a delivery requiring an emergency caesarean section. So together with that history with the examination finding is the head down or is it a transverse lie is it the breech for example is it a two previous caesaer or whatever is it a bigger baby than the passage of the birth canal or those now this is what informs the final diagnosis on whether the woman will be requiring an emergency caesarean section or not. Now once the decision is made because caesareans are not just from nowhere the normal birth is supposed to be vagina and we desire as much as all women could deliver vaginally. But there are medical or obstetrics indications for caeser where you are left with no option, and those are the ones that fall into the category of emergency caesarean section. I just wanted to say a bit that emergency means that it is zero option you have to do the caeser anyway to have a good outcome both for the mother and the baby**.** Then there also other caesarean sections which are elective and you already know the background and plan that this later cannot go through the normal vaginal delivery of birth and you plan a date for caeser before they go into labour ok I will give you an example if someone has had two or three caesers you can't allow them to labour so we will plan for a caeser before the onset of labour another example if one had previous caeser for many pregnancies in short maybe eighth pregnancy they had one previous caeser then you plan examples of emergency caeser ok maybe a two or three previous caeser then the labour starts before the planned date you gave for the operation maybe the cord has come out the water is broken called prolapse maybe there is a transverse lie meaning the baby is laying across in the womb there is no way it can come out you understand or maybe there is a low laying placenta meaning the placenta is on the lower side of the uterus and then the womb is bleeding we call it antepartum hemorrhage within such that if you don't intervene the woman will die. You can also have … placenta where the placenta has prematurely separated and so the .. bleeding. If the baby is alive you want to salvage the baby ok. Another example of an indication for emergency would be ah for example you have fetal distress meaning labour has started and it could be progressing well in terms of the passage opening but then the baby has gotten tired and you pick it by either the heart of the baby going too slow or going too fast, the baby has pooed inside, those are the

cases. So once the obstetric indication has been made that has to be communicated to the woman to say from what we have found out from our history especially the examination it has confirmed that you will not manage to go through labour we will need to deliver you through the alternative delivery which is caesarean section and we cannot wait because probably you are already in labour that is what makes it an emergency because we cannot wait. So you need to communicate the condition very well to the woman where I have highlighted some of the indications to tell her that the baby is tired if we don't deliver you now the baby will die inside your womb. For example to say the cord has come out and the cord, if this cord is exposed to the air within five minutes the baby dies. So we will need to keep the baby alive by holding a hand inside you to keep it until the operation is done, you understand? to say if for example if you had two three caeser if we don't operate your uterus will rupture chizang'ambika chibalilo and that would give a risk to loss of life of you the mother and baby. If the woman understands, they will need to show that they have understood and it is at that point that they are asked to sign a consent form. A consent is an official agreement of the information given and the decision made. It is an informed consent meaning the consent is being signed after understanding the information and then we proceed with the emergency caesarean section. There also others that probably it could be their first experience to undergo the caesarean section we also delve into sharing what caesarean section is how it is done, the risks of the caesarean section for example we have to explain that you will go into theatre you will be given an anaestetic drug those are drugs that are meant to take away the pain as the operation is taking place and those also may have their own potential complications. So we explain everything. So when the woman consents then the name is taken to theatre we have an emergency the name is taken there and the woman is brought to theatre. Even if it is an emergency all the same the midwife that is bringing the woman to theatre will still need to explain to hand over to the theatre team, the theatre team there are theatre nurses, anaesthetists and there is also the surgeons ourselves so they will need to hand over to make sure that all the preparations for surgery are well done. It means that the consent has to be approved that there is a consent the patient knows what she has come to theatre to do. You can't do anything without the patient knowing. We will need to know the hb what is the level of the blood, hb is also part of the hand over we will need also to confirm that certain antibiotics are given before the operation. If a bleeding patient there also other medicines we give to reduce further continued bleeding all

those they are handed over if it means catheterized the patient is shaved whatever everything is handed over and the anaesthetist will not take it for granted that ah you know he will do his own assessment is this patient fit for spinal anaesthesia where you numb the patient, is the condition of the patient dangerous for spinal maybe we need to go for general anaesthesia where you put the patient to sleep. Does the patient react to any medicine least they react to what you are going to give them? All those are important things that are done but it happens simultaneously you are taking the history, they are handing over uku (there) things are happening you understand so that they are as quick as possible. The aim is that for an emergency case it should be done within thirty minutes or sixty minutes the delivery should have occurred unless the circumstances are extenuating where for example you have so many similar emergencies yet you, you have immediate operating space then you find that others go up to two hours ok. Basically that is the scenario for emergency caesarian section. And then after the operation, these women need to be counselled further on what to do to avoid further complications for example if it is spinal anaesthesia they may not need to rise up in bed they need to lay facing up for at least eight hours or so because if you rise up early there will be complications of the headache because of spinal anaesthesia. We also counsel them that the patient will start after the anaesthetic drug finishes in the body but then we transition them to the post-operative medicines, which is a concoction so that the woman is as comfortable as possible as they can be after the operation yeah. Basically that is what it and the team remains open.

Interviewer: So apart from communicating the procedure, indication for emergency caesarean section and the risks, what else do you communicate to the woman?

Participant: ah basically it depends on the time of emergency because for others you may not even know of what will come out even as the medical team for example, cord prolapse or fetal stress you give hope to this woman that the situation for example is not good and so you need to give them reassurance that the team will do their best but sometimes the actual outcomes may be outside your power you understand? And so you want to give hope to this woman hoping for the best both for the baby and woman.

Interviewer: How much time do you have to communicate to a woman who require emergency caesarean section?

Participant: it is very difficult to allocate time. It is scenario based for example others come in shock will not even be part of the consent. It will require you know a third party consent, collateral ok yeah so it depends, it is case to case. Those that are ok they are well oriented they are not stressed. For example within five to ten minutes you should be able to get the information and remember most of them are not medical people so there is also you develop a skill of communicating the most important things if you give them too much information in a stressful situation they may not hear anything. So you become selective and carefully professional, what are the main things that I need to communicate even before the consent so it is not like a package of everything it is scenario based case to case. The most important things are to make them understand why they cannot deliver vaginally and why it needs to be done as an emergency that is very key. Sometimes even as you are operating you communicate other things which you thing could have been important to the patient. That is how it is done.

Interviewer: what are some of the challenges that you face as medical personnel to communicate to the women that undergo emergency caesarean section?

Participant: it is ignorance, there is a lot of illiteracy yeah around and unfortunately that is the majority. It is very difficult to communicate with an illiterate person because conventionally they are illiterate but customarily and traditionally they think they have a lot of information that may not conform the conventional information. So sometimes you have difficulties to get the understanding of this person. Others would come so maybe the baby is too big for the passage which is normal people come with a five kg baby and then the passage maybe can only allow three kg you tell them the baby is too big and traditionally they were told if the baby fails to come out for example it means your husband was hitting out (cheating on you) you know what I mean? So now in our context you need to have some cultural understanding ok so that as you are communicating you also put yourself in their shoes but at the end of the day the goal is to have the information communicated. Then there are other people it is an emergency then they don't want to believe the medical advice. They instead believe the advice from home either from the parents, from the in laws or husband. So you find that someone so is gasping almost dying and then the people she has come with are saying we can't consent or this woman is conscious she says I can't make a decision on my own then the one who is not even a doctor dillydallies to make a decision and they delay the intervention.

By the way we have had bad outcomes because of delays in making a decision remember we will not operate on someone that has not consented. We cannot. In medicine the patient has the right to refuse to be treated. Others have refused and have ended up dying and there is nothing we can do. **S**o it is ignorance, illiteracy very bad. The other categories is the google literacy, literate but through doctor google this person thinks that they can have better information than you because they have been googling. It will never happen you understand because they say no when the baby is what I have googled and you know with uncle google if you just google headache just have a headache and google headache what can cause a headache you will have one thousand diagnosis my dear you can even become depressed and die yourself because you are thinking I have all these conditions ha ha ha so there are people with uncle google's attitude, very difficult to counsel because they think they know it all ok. The other challenge we have religious beliefs very very tough someone will come with three previous caeser and they will tell that they were told they will deliver vaginally we had a scenario by the way two ladies from Nigeria one was a previous caeser one was two previous caeser and they were next to each other so they have all these oils they are applying they are saying we were told. So we said ah telling nimwamene (it is like that) we are also believers ayi (right) but God has given us understanding and so with these you risk developing complications on your lives let's do the caesarean sections and they were both in labour. You know what happened they refused and then the one who had the two previous caeser the uterus ruptured and then the babies crushed she went in shock the whole army of health workers is called to resuscitate to bring her back to life. The other, other one started shouting ati why are you delaying do the caeser on me you understand ayi so I was like we need to attend to your friend first you will be done. So those are some of the issues. I think it is the way as Africans we have taken religion it should make us shutter our thinking it is wrong. We really face challenges. They will just say papa is praying for us and they are on the phone they will say let me get the guidance from papa. But basically speaking it is supposed to be an emergency operation. Those are some of the key issues and then sometimes the underage we have a lot of emergency caesarean sections for underage and so huuu God and sometimes you have no one to consent for them. They are children 14 years to 15 years, they are kids so even to understand what is happening they have no clue. It is difficult so if you receive an emergency and you have an underage they have to sign the consent even before they are seen to avert problems who challenges because we will

not operate when there is no consent. However in the absence of collateral consent or by the guardian whatever the consultant or the coverage nurse or superintendent we are able to sign on behalf of these underage. Sometimes even the mentally disturbed yeah.

Interviewer: How best do you think you can deal with some of these challenges that you have mentioned?

Participant: I think the summary of what I have given is what we currently do try your best but I think there is a lot of public health education that is required we have not done well as a society as a community as a country you will only understand lack of information when you work in our environment. Maybe one day just come and sit with us even in the emergency room you will cry that there is absolutely no information going out there. So a lot of people are doing research like yourself and it will end in the dissertations and degrees. We need a lot of public health communicators communicate to people at their level but driving through the key messages and I don't know it just needs everyone's efforts deliberate public health communication tools religious leaders, traditional leaders and so on. Maybe taking advantage of the social media platforms also put up gazette sites that can give out correct information to people and not try to tap from the ocean google.

Interviewer: When do you think would be the best time to speak to these women?

Participant: it is antenatal so remember that when someone is pregnant we advise them to start antenatal early and a good antenatal service will show the attitude of these women even though they were to convert to emergencies, it is during antenatal that you would have more time to talk about the potential complications and potential emergency situation including the delivery plans you tell someone who has four caesers that you will deliver by caeser we will set a date. But in case labour starts before that date wherever you will go you will be delivered by emergency caesarean section so and what happens at antenatal when they women come they take the vitals and there is health education and at the contact of the one offering antenatal it could be a doctor, nurse whatever it is emphasis now which is tailored to the individual because every pregnancy is different so and individualized care and then you discuss everything. The thing surrounding that pregnancy, what is the best mode of delivery in case things happen so the woman is supposed to becoming empowered but to add to those challenges we have some women that have very poor health seeking behaviour maybe

antenatal someone has just been there once or twice so others have never been to antenatal and this is when they coming and it is an emergency. They have no room for contact or information empowerment during antenatal services.

Interviewer: What is your assessment of the antenatal services countrywide?

Participant: we are still struggling and the factors are multiple, I will start with the commonest problem that has given substandard antenatal is the lack of skilled man power to conduct antenatal care services. So in other parts of the country you find there is even no midwife so the ones that conduct antenatal are not midwives, we still have some rural areas where antenatal care is offered by none medical staff environmental health officers, the cleaners, the guards offering services for antenatal including conducting deliveries. It is very sad ok, later on the antenatal suppliers like the blood boosters you find they are out of stock most of the time so you find some complicate with anemia are not supplemented later on the outra sound scan which should have picked this low line placenta may not be available everywhere and you find that this woman has no clue where the placenta is so labour start innocently the placenta is low line and they will bleed torrentially ok yeah and sometimes its long distances, traditional beliefs. Later on patient factors they are just patients that you never understand they just choose not to go for antenatal they have the facilities within reach but they are just bad people.

Interviewer: As we conclude the interview is there anything of importance that you would like to talk about regarding communication between the women who undergo emergency caesarean section and health care providers?

Participant: I think generally with the little experience I have, health workers do their part but if these women were primed during antenatal the work would be much easier if the negative pieces of information whether religious or traditional could keep reducing then this job of communicating could become very easy. Otherwise I think the health workers do their best but I would implore the public health specialist not to be a class based professional we need to feel your impact that is why it is public it is not classroom it is not private it is public health take that health information to the public and it will show.

Interviewer: Sir, thank you so much for your time.

Participant: bless you ha ha ha ha.

PARTCIPANT NUMBER FOUR (CO4)

Interviewer: Sir, welcome to the interview.

Participant: Thank you. Good morning.

Interviewer: Good morning. What is your role when dealing with women who require emergency caesarean section?

Participant: My role is seeing and attending to the patients and being able to come up with an appropriate diagnosis with somebody who needs caesarean section being it elective or emergency caesarean section.

Interviewer: Please describe the communication between health care providers and women who undergo emergency caesarean section before they are wheeled into theatre.

Participant: Well, basically my role because I am a Consultant, so in most cases these patients would have been seen by juniors sometimes you will find the diagnosis or decision already made and my role is to verify and maybe augment the information or the management of treatment and just to confirm whether that line of management has been initiated or not. Then sometimes a patient could be on another line of management it is my job to determine whether that patient should go ahead with that line of management or indeed change the decision, prescribe what is appropriate as a consultant.

Interviewer: Kindly describe the process of consenting to emergency caesarean section.

Participant: So the process is as follows, first of all upon discovering that there is an indication for caesarean section be it emergency or elective, the patient is told the findings and the reason why we are proposing or rather we are telling her that is the best line of management. The indications could be maternal or fetal factors. So if it is the maternal factors we tell her our findings and whatever the particular diagnosis issue or clinical manifestations that are indicative for one to undergo emergency caesarean section and indeed if it is a fetal indication we communicate with her. So there are so many indications for caesarean section being elective or emergency. So emergency in most cases that means if it is maternal indication that means it is risk for the mother to go on with that pregnancy because it will cause other complications maybe morbidity or indeed death or mortality. The same applies maybe somebody could have

had a pelvic fracture that is also an indication but depending on what type. If the patient is not in labour you will treat as an elective, if they are in labour and somebody has the history of caesarean section it is dangerous for her to undergo labour it is also prescribed that the patient undergoes caesarean section. But again like I said a scenario like that if a person is in labour that becomes an emergency but if they are not in labour you treat it as an elective. So depending on the scenario you explain to the patient that this is what we found and this is what we are recommending as the best management based on the findings and communicate to the patient. After we tell the patient then we also tell them that you need to help us sign an informed consent form then that is done.

Interviewer: What are some of the challenges that you face when communicating with the women that require emergency caesarean section?

Participant: So the challenges first of all, let me put them this way, it is an emergency case, somebody who is a minor cannot consent and the next of kin are not around yeah. So we will have to communicate to them (patient) can you call your parents or whoever the guardian is because age of consent is 16 years. Now in an emergency situation there is no time for that there is a provision according to standard practice and I think the law but that you need to check, the administration or the senior most in the team consent for on behalf of that patient in a life and death kind of scenario, if it is at night the senior most signs, if the system is operating optimally like during the day, the [name of position removed] and if it is at night the night [post removed] does that, the Consultant or the Senior most person who is available is mandated to consent for and on behalf of that patient. It does not only apply to the minor but even an adult patient who can't consent maybe in an event that they are in a confused state we communicate to the next of kin or they are comatose yeah you know scenarios like that. If it is a life and death scenario, the system comes into play. The other challenge is when denial, this is a patient who is just in denial you know there are perceptions, this is good for you maybe in another setting sort of qualitative research. Certain beliefs that for you to be respected as a woman this is just what we hear if you to be respected in the community you need to have experienced vaginal delivery by yourself as opposed to be operative. So we see certain number of women though they may not directly communicate to you directly but we see no I want to deliver on my own things like that even when you told them during antenatal that you need to

come to the hospital early because you cannot undergo a normal delivery, you will need to undergo caesarean section they deliberately come late with the hope that they will waste a bit of time and be able to deliver and sometimes unfortunately we end up with bad outcomes. There is that denial aspect which becomes a challenge yeah. So then sometimes lack of autonomy it is against the human rights because again the human rights a person over the age of 16, a client has got the right to access health and also access to confidentiality all the privileges of the whole continuum of care, they have got the right so but again despite that we have a good number of women who cannot make independent or who don't have that autonomy to make a decision, they have to wait for the husband or the relatives and things like that so that is the other challenge. So sometimes you find that there is a delay in conducting the procedure because of such challenges.

Interviewer: Even when they are fully conscious and of consenting age?

Participant: Yes, of sound mind but in denial. Denial can be because of some many things because of myths, maybe we have to intervene because of prematurity but its life and death because of maternal and fetal aspects and they are wishing otherwise but our medical expertise find otherwise that this pregnancy cannot go on, they are wishing things should be different but if the pregnancy goes on we can lose the mother or baby. That is the other challenge which delay to obtaining consent at an appropriate time.

Interviewer: Who obtains consent or who reads out the consent form?

Participant: So normally the attending doctor will tell the patient the findings that this is what we found and this is what we think you should undergo so after that obtaining consent the nurses will come on with further counselling until the consent is signed. So the doctor will start then further counselling follows, the nurse or sister attending, that is the person who does that. The other scenario recently it's been kind of adopted as standard practice especially in the emergency points where even when somebody is stable we call them cold cases. An informed consent for surgery is obtained at admission. This is something that has been adopted as standard practice over time because of the challenges I mentioned earlier somebody is in shock and there is now a challenge to get consent. So that has also been adopted right on admission patient is counselled that is this blah blah do you accept the hospital can take these life saving measures and what so yeah straight away somebody consents. So that makes life a little easier

because sometimes you have a scenario where somebody has a caesarean section that is a complication but that patient let's say there was hemorrhage and the uterus maybe cannot be preserved for us to preserve the life of that person we have to remove the uterus but the patient is under anaesthesia so that gives us a leeway in dire situations give us a leeway otherwise if that was not obtained but you are in an emergency the decision is now made by the senior most even if he is not physically in theatre he has to be informed that these are the findings, permission to go ahead and the senior gives guidance whether there is an alternative to go about it or goes ahead. So the system is in seniority. So that is what happens.

Interviewer: So apart from informing the patient on the indication for caesarean section, what else do you tell these women?

Participant: we tell them on complications of surgery, complications can stem from anaesthetic, there can be excess bleeding, there could be injury to the surrounding organs, injury to the bladder, injury to tissues like urethra that is where the urine passes into the bladder, there can be an injury to the bowel yeah then after that sometimes there can be infection immediately after the operation the wound may fail to open, to heal. You can end up with an abdominal infection. There could be subsequent surgery because the healing process is not ok, one might end up losing the …. If there is bowel injury the operation might be bigger. So all that information is supposed to be communicated to the patient though the depth of counselling is also dependent on the case and complexity of the operation but where you don't anticipate such the counselling of obtaining consent is not that detailed yeah. So normally people will just touch there might be complications but the patient is just told what the procedure will be, the likely anaesthesia they are likely to undergo whether they will be awake or completely asleep yeah

Interviewer: Since it is an emergency, how much time do you have to communicate to the woman?

Participant: no it depends if it is an emergency it is a very short time, five ten minutes as you are counselling the patient someone is preparing the patient. Then sometimes ok we are talking about consent so it is relative. Sometimes there is no time you need to save this patient you start doing what you have to do. You just tell the patient look this is what is going on blah blah we are going to start doing abcd maybe they are bleeding, we need to save this baby.

Sometimes there is no time for the patient to sign you just use your thumb so depending on the urgency.

Interviewer: When do you think would be the best time to speak to these women?

Participant: the best time is immediately you diagnose emergency caesarean section but during antenatal. It is the moment you diagnose it because they need to understand, understanding is easier to accept consent but if the understanding is not good then you face challenges they will be can't we do that, what about this can I get a second opinion [hospital name and additional text removed]. However with an elective case the patient needs to be counselled and prepared psychologically during antenatal period when there is an indication because it also covers elective, isn't it?

Interviewer: No, my focus is on emergency caesarean sections.

Participant: You should have included electives as your second objective.

Interviewer: Yeah, maybe next time. Please describe the communication between health care providers and women who undergo emergency caesarean section after theatre.

Participant: Communication, normally it is purely ward kind of arrangement. We get feedback after ok if it was a normal operation where the patient is not in a critical condition, the patient will go back to the ward and they are taken care of by the junior staff. Critical patients are taken to the critical care wards, there we are able to see them and find out their recovery. Normally it's the opportunity to explain to them depending on their state of mind what was done and what we anticipate. If it was done by somebody else we want to know if the procedure done was communicated to them if it was not done we get the details and communicate them to the patient, what was done and what is implied and then we just manage the way the patient is.

Interviewer: As we conclude this interview is there anything of importance that you would like to talk about regarding communication between health care providers and women who undergo emergency caesarean section?

Participant: Thank you very much for taking this initiative it is an interesting one. In our set up most of the people doing research concentrate more or our research is predominantly

quantitative so it is a very important undertaking that you have you have conducted. The findings will give us feedback on our care and it is a kind of assessment on our care how best we can do things and also inform not only policy but also standard practice and care and how best we can improve our protocols. Yeah so it is an interesting one I wish you collaborated with one of our guys on the ground because the set up in a tertiary hospital is different from a set up in other hospitals because the circumstances are different. I guess those will be your recommendations because here we are a referral hospital and receive complicated cases yeah but for normal caesarean section but I guess it gives room for somebody else or you yourself at PhD level, you might start thinking about your PhD. Otherwise thank you so much.

Interviewer: Sir, I would like to sincerely thank you for your time.

Participant: You are must welcome.

PARTICIPANT NUMBER FIVE (CO5)

Interviewer: please tell me your role in dealing with women that require emergency caesarean section.

Participant: my role is talking to the women about the decision for caesarean section, often doing the emergency caesarean cases as well.

Interviewer: So what is the standard of communication for emergency caesarean section?

Participant: Typically we try to use simple language that we can if possible that we can use local language we try to communicate what the patient understands best where we are able to utilize that language as well and then try as much as possible they understand a little bit about the condition that has necessitated the need for that emergency operation yeah

Interviewer: Please describe the communication between health care providers and the women that undergo emergency caesarean section before they are taken to theatre.

Participant: so typically what we will be trying to put across because when a decision has been made for an emergency operation the thing that is cardinal is first to explain to the mother the benefit of why we have to do what we want to do and possible consequences because most of the time these are not absolute things you sort of pick a few things that are likely to give you a bad outcome you try to sort of bypass those anticipated challenges by offering this other

option that is there. So we usually try to give a few positives of what we are trying to do and our anticipated negatives of course which sometimes is lacking especially when we are going for an emergency procedure because that length explanation you don't have time to explain a lot of those things. Most of the time we are focused on the benefits and the disadvantage of probably proceeding with the method the normal process that is non operative intervention. Once we have passed that information onto the mother and she is somehow acknowledged that she understands then we proceed to offer consent for the operation which most of the time most mothers will agree because it is going to benefit them and their babies. We obtain written consent.

Interviewer: You mentioned that time is limited, how much time is needed to communicate to a woman who requires emergency caesarean section?

Participant: typically probably **t**he whole process of counselling will probably not take more than ten minutes depending on the circumstances. Most often under five minutes would have at least managed to communicate a few essential that we need for the woman to consent.

Interviewer: Please describe to me the process of consenting to emergency caesarean section.

Participant: So the process of consenting always starts with information providing information to the mother about why we are going to do the operation and the operation we are going to do. Most often there are no details per say or the technicalities of how there are going to do it but we do give information on the process of doing an operation which we call a caesarean section implies delivering a baby through the abdomen, which means we are going to cut to remove the baby at least we give this as basic information and we do inform them of course about anticipated challenges tentatively they tend to bleed more compared when they have a normal delivery and things like they usually tend to stay in hospital a few more days to allow for healing and monitoring post operation yeah and once that has been done then we go to the actual process of then consenting in this case we provide a form to them so that they can read through either append a signature or put a thumb print.

Interviewer: Who obtains written consent from the patient?

Participant: typically it is the medical doctor who obtains consent from the patients but most circumstances we don't get the written we usually do it in collaboration with the midwives.

Consent is usually obtained by the midwives, the counselling and the verbal consent is done by the doctor who is doing the operation.

Interviewer: Is that the way it is supposed to be done?

Participant: the standard is the person who is offering the procedure is the one who understands the technicalities and should get the written consent because the information on the consent has to tally with the procedure, the things that you have discussed pertaining to the intricacies of the operation. So it is the person who is offering the caesarean section who has to obtain the consent.

Interviewer: But why is this not being done?

Participant: I don't know why it is like that but that is the organization we have inherited and we have continued with that practice yeah.

Interviewer: what are some of the challenges that you face when communicating with women that require emergency caesarean section?

Participant: commonly sometimes you find despite the counselling someone might still want to have a try at the normal birth even when you have communicated that there are challenges that are involved. Sometimes there is always usually thoughts that perhaps you did not communicate adequately to the woman most of the times we may have someone else try to talk to her, other doctors including the midwives to try and talk to her whether they will manage to get through to the patient. Occasionally communication, the reason for wanting to do the operation sometimes it is very difficult to simplify it to where a layman can understand and you find that we usually tend to give half-truths so that the patient can just try to understand what you are trying to say.

Interviewer: Describe the communication between health care providers and the women when they come back from theatre.

Participant: I think post operation that is where we probably lag a little bit because most of the uncomplicated operations we rarely see them after the operation. So most of the communication if at all there was a challenge it will be during the time of the operation and

post operatively the care will be under a different officer where they will communicate and counsel the patient, sometimes what was done in theatre lacks there.

Interviewer: As we conclude the interview is there anything of importance that you would like to talk about regarding communication between health care providers and women who undergo emergency caesarean section?

Participant: I think from my observation I feel like we never have a chance to give adequate information concerning the procedures that we do especially where problems are involved most of the time we give information about the good things but we don't communicate the potential bad things that may happen and we keep seeing patients coming back one, two three five years after the operation and they are complaining about something that we never mentioned during the time that we had done the operation. What I would love to see is something that someone can read reference people can carry home with them some of the things we miss in terms of communication about the procedure that they can read and even come and ask after we do the follow ups I think that is something I would love to see included in our system as well.

Interviewer: Doctor, thank you very much for your time.

Participant: you are most welcome.

CONVERSATIONS WITH KEY INFORMANTS

PARTICIPANT NUMBER ONE (KI1)

Interviewer: Doctor, welcome to the interview.

Participant: thank you.

Interviewer: What is your role when it comes to issues to do with emergency caesarean section?

Participant: my role is to give instructions, sometimes is to do the actual caesarean section or sometimes is to correct a mistake if there is a surgical problem complication yeah.

Interviewer: please describe the communication between health care providers and the women who require emergency caesarean section before they are taken into theatre.

Participant: the general communication is that the next of kin is involved because when you talk about emergency caesarean section our situation here emergency caesarean section can be a person in a coma, can be in a person who is fitting for example convulsing that is one of the largest cause of emergency caesarean section when someone has a problem we call it eclampsia. She is not talking, she is not in a mental faculty to sign so that is why we consider a next of kin and if that person is under sixteen the things come to me because things have to come to me and we sign representing the management and from there on if the consent is obtained that is when we inform theatre and the procedure is done. After the procedure is done when the patient is fully awake most likely after forty-eight hours if it was general anaesthesia we talk to them again and explain what happened unfortunately we don't sign I think we should be signing when the communication happens.

Interviewer: what information are these women given before they are taken into theatre?

Participant: 1. they are given their diagnosis 2. They are given a choice if there is a choice a caesarean section and another forced way of delivery or to just wait for nature. If their education standard is good enough they are even told the percentage of survival in case of choices so that we give them information in which to base their agreeing to emergency caesarean section or they don't agree to emergency caesarean section even if it is not themselves the next of kin is given that information and then they are asked to sign an agreement and when they disagree we have very little choice the only choice is get a second opinion [text removed], we still say get the second opinion from [hospital name removed] or somewhere else.

Interviewer: What is the standard of communication to women who undergo emergency caesarean section?

Participant: I would say that the standard is that the most senior person in the team that receives a woman who is convulsing usually leads the communication, usually leads the two way conversation either the relative or patient herself.

Interviewer: describe the process of consenting to emergency caesarean section

Participant: first it is information given and this information is followed by a session in which a question can be asked and then clarified and then at that point a patient is called in to sign

whether they have agreed or not agreed and then the medical worker who is witness at that time is asked to sign the consent form.

Interviewer: Who obtains the written consent from the patient?

Participant: the one who did the counselling usually if it is a nursing care for example if the emergency in the labour ward usually this is delivered by the midwife yes, the midwife who is taking care of that person.

Interviewer: is that the standard?

Participant: yeah that is the standard, well the one who does the operation may not be there because part of the consent was that I understand that I have not been assigned a particular doctor to do that operation because we have many doctors there are in a rota. The way we do it here it is not the doctor who talks to the patient unfortunately is not the one who will do the operation.

Interviewer: Since it is an emergency, how much time is required to communicate with these women who require emergency caesarean section?

Participant: emergencies by their own standard, our standard that we are struggling to follow is from decision to the time the knife is put on the skin it should be thirty minutes. How we are able to achieve that? Sometimes we are able to achieve that but over eighty percent of times we fall off that thirty minutes the standard we try to set ourselves sometimes we are unable to achieve that one.

Interviewer: What challenges do you face when communicating to women who need emergency caesarean section?

Participant: there is posit of information if and when literacy levels are considered. This is not only for the old be who maybe illiterate it is also for the very young ones who are mostly actually people who have emergencies it is fan, it is ironically that people who are in the brackets are the ones who are at least able to understand, the old ones, the younger ones are the ones who have conditions like preeclampsia. So you find the literacy level to understand the diagnosis that you have just been fitting and if we don't deliver the baby, the baby will die or you will die, they may not understand, that is the issue that we have. The second problem

sometimes relate to age because you see the consent age which has been put at above sixteen we receive a lot of emergencies at 16 and below but in fact the proper age is 21, I will find out if this is still at 21. You still find teenage pregnancy they can't sign the only person who signs is the head of clinical care sometimes times they have to mobilise the night superintendent this tend to delay action.

Interviewer; please describe the communication between heath care providers and the women who undergo emergency caesarean section after they come back from theatre.

Participant: the communication after they come back from theatre the first twelve hours we avoid talking because of the pain. Sometimes we start talking to the patient on the forty-eight hour window period because we know that they can't digest when they are in pain after a caesarean we do try to give medication and it alters perception because it is opiod nature. So we don't want to talk during that time but when we do talk it is doctors when they are doing rounds, the senior most explains to the patient what happened. Some of them by the way don't even know what happened, it was an emergency they don't know so the senior most person in the round will explain and ask for questions because an operation has future consequences so we have to explain the future consequences what is the fertility prospects, do you want future pregnancy, will there be even possibilities of future pregnancy so those are the things we discuss.

Interviewer: As we conclude the interview is there anything of importance that you would like to talk about regarding communication between health care providers and women who undergo emergency caesarean section?

Participant: I think sitting in this office has opened my eyes to the fact that documentation is very very important we get the documentation on the consent but on the post-operative there is no signature and we do have women who come ten years later to say I am failing to get pregnant doctor and yet we removed the uterus because there was bleeding. So we find a situation where someone says no I wasn't told but we don't believe those stories or they don't believe our stories so it becomes a thing of who is lying so I think that if anything that is what we might introduce postoperative consent, it might look like just a discussion I don't know the name we will call it, they need to be signing about this discussion.

Interviewer: thank you so much for your time.

Participant: ok.

1. **PARTICIPANT NUMBER TWO (KI2)**

Interviewer: doctor welcome to the interview

Participant: thank you so much for the opportunity.

Interviewer: Please describe the communication between health care providers and the women that undergo emergency caesarean section before they are taken into theatre.

Participant: so ideally before you take them into theatre you need to discuss with them what you want to do about this. So the initial communication is first you assess that you can speak in the language which they understand because we know most of our women will not be able to get it better in English so you assess what language are they going to speak if it is Bemba, comfortable with English sometimes you find you don't click on what language Bemba you find that the other person is Lunda and you can't talk so you want the best person who knows their language. So the communication is such that it is simplified to the patient's own understanding. So it involves reducing the language to the non-medical language so that the patient understands. So the communication involves telling them what the diagnosis is, what is the problem you have seen, then from there you talk about what are the implications of this diagnosis then what you want to do in the theatre and why you want to do it.

Interviewer: Since it is an emergency, how much time is required to communicate to these women who need the surgical procedure?

Participant: so for emergency caesarean sections they are actually grouped into three; so there are those that are in group one, those that really need to be done fast, those mean that a patient should be operated within thirty minutes of making a diagnosis so in that thirty minutes it means that you must communicate to the patient and prepare them and move them to theatre. So what that means most of the emergencies we have, we have less than ten minutes to talk to them another ten minutes to prepare them then the next ten minutes they should be moving to theatre, the baby should be taken out within thirty minutes of making a diagnosis.so we may have those we call semi there are not really, they can wait a bit for example the baby is coming

with the buttocks but this baby has got more than thirty minutes you can have that time but there are those where the baby’s heart beat is going down, you know that what will save the baby is how fast you will take them to theatre. So you have ten minutes or so to talk to the patient you have another ten minutes to prepare another ten minutes to move into theatre.

Interviewer: describe the process of consenting to emergency caesarean section.

Participant: the process of consenting starts with explaining after assessing the patient you explain what the condition is, what the diagnosis is what the problem is we would say the baby’s heart beat is going down or we have seen that the baby has done poop inside the uterus so we have seen that the water is dirty like this so you are explaining the condition. So when the patient has understood the condition then you go on to propose what you think should be done to the condition. So you say in the interest of the baby I think the baby should be delivered by an operative delivery which is caesarean section. When you mention the caesarean section you don’t want to make an assumption that the patient knows what a caesarean section is, so you go on and describe what a caesarean section is, this is what is involved and during you may have issues of anaesthesia to help with pain relief and the options of that. You talk about why you think that caesarean section is the best at this time, the benefits. You also talk about the problems of not going for caesarean section, what problem may you encounter, the complication which may arise in theatre and beyond theatre 20 years from now. Then you talk about what other options does the patient have if we say caesarean section is the best what is the next best because there are some patients who are about to deliver, so they may have an option of going for caesarean section or an option of what we call a vacuum to pull the baby under distress so you talk about, the options and the benefits and complications of the options. Then having done that the next process to assess the understanding of the patient. So this involves asking her to tell you what you have discussed with her, what you have understood so she will tell you, you told me my baby is not fine the best is to go for an operation she describes in her own understanding. Then having her described then ask her if she has any questions which she wants you to patch up then she asks the questions, when you have addressed those questions that is when you say I want you to put this in writing or by signing that you have understood, which is the consent.

Interviewer: Who obtains the written consent from the patient?

Participant: so the written consent, there are two scenarios in few circumstances that is obtained by the doctor who has made the diagnosis. That is the standard so if I say I am taking you for caesarean section I should explain to you what a caesarean section entails and ask for your signature to say I have to take you for caesarean section. But most of the time that is not what we see reason being that you may have one doctor doing a round in labour ward. So the doctor will make a diagnosis and they are working by the midwives so in that case may come in to finish all the explanation and consent process. Majority of the consent we see it is obtained by the midwife on duty.

Interviewer: What are some of the challenges that you face when communicating with women that require emergency caesarean section?

Participant: so I think the challenges we have one of them is the limited time where the doctor is not able to first you are limited by the emergency you have you would want to take thirty minutes also to talk to this patient but the emergency itself can't allow you to go through all that reason being you are aware that this baby needs to be saved by going for caesarean section. You need to summarise it in such way that you leave time to take the patient to theatre. The other limitation is that at times there may not be good numbers of doctors to allow you to spend all this time on one patient while others are screaming in the same room because you are going to start explaining, explaining the other patient needs my attention, so your mind gets divided. So you find that that time to see other patients and the time to deliver this baby. Rarely we see concerns of the language barrier of seventy three plus languages you may not speak the same language with the patient so you may call in another person to come and explain who may not explain the way you want it to be explained but being in [removed] most people understand Nyanja, they can speak broken Nyanja the patient will still get it but I think the barriers mostly it is the time because other patients need to be seen and the limited time you have to explain before you go to theatre.

Interviewer: You mentioned that you have limited time to fully communicate with these women, when do you think is the best time to communicate to them?

Participant: the other option is to discuss these things during the antenatal period. So we have the antenatal lessons which are done during the antenatal clinic so when they women come for example to [Hospital name removed] they come like 7:30 or so and then they are taken through

these lessons so during those lessons that is you talk about the common signs of labour and it is a good time to talk about caesarean section to mention what conditions would make someone end up with caesarean section, the complications and what options would be available and during that time we want to these women their mind to say anybody can end up with a caesarean section it doesn’t matter even ones who have delivered before those who have not yet delivered everyone should know that there is a possibility of caesarean section. When you are not in that condition sometimes the dearth of understanding may not be as much as when it really affects you, you say this is not for me so the understanding may not be as much as when one who is going for the caesarean section. So it is for that reason that we feel if it was mentioned during the antenatal it needs to be explained to this person again to say this what it is but the best would be to start during antenatal so that we may not need to go into the neat gritties of the procedure but just to explain a bit before she goes for caesarean section.

Interviewer: What is your assessment of antenatal services countrywide?

Participant: I would say the system is overwhelmed yeah because I think I have been to a new MCH where you see that the ratio the number of women this person has to attend to is too much compared to what they are supposed to do. One person for example attending to fifty women there they may not do the best they need to do in terms of assessment and what we see is that sometimes women who should have been referred to higher centres in good time because there was not enough time to prepare each client to say this one is to go to a tertiary hospital and sometimes they are picked at the end but the situation where somebody has a caesarean section and the system hasn’t picked this woman until when they reach labour you actually this one had a caesarean section all because the system was overwhelmed otherwise everyone is aware as far as the hospitals are concerned but just that the care provider to the patient ratio we haven’t reached where we would say we would be happy the numbers are ok for example under my unit here we may be like five seven doctors doing antenatal sometimes we attend to women in excess of hundred and twenty in one session and these are not like straight forward cases this is a referral setting they have diabetes, they have hypertension and so forth so you can’t take them as routine patients so there that system is overwhelmed for people to provide antenatal care and we see it from patients complaining I have been sited here the whole day so that is telling us something yeah when somebody comes they will say I have been waiting to

seeing the whole day doctor I came at 7:30 hrs and this is 17hrs then you just say we are really overwhelmed.

Interviewer: then how best do you think we can deal with patient health care provider communication?

Participant: so I think we need training for health care providers because the way I know the subject is not equivalent to talking to somebody about it if I know how to do caesarean section I may not have the skill to communicate to the patient this is how we do it. But I can explain it to my fellow doctor into simple language we may need to have that training I think I have looked at our medical school for example I think it is one of the deficiencies I think we are trying to introduce it in their curriculum even into the exam like one of the clinical skills we examine them in communication so we trying to train them at an earlier on stage in the training to say when you are talking to the patient you need to know what language to use to assess the understanding like I am talking to you I should be looking at you like not looking somewhere else or whatsapp like that so that I can assess because there are times you are communicating the patient breaks down they are in tears because you are not paying attention you will not know that they are getting emotional. So one way is that you are taking into the training and making it examinable we know that we are going to assess how you communicate to a patient so as they start picking it earlier on they know how to speak to the patients so we don't want a doctor who is using the medical jargon to the patient that will not do justice to the patient we want the patient to understand it is not just a matter of just saying it for the sake of saying it but also rely information then you would have communicated effectively.

Interviewer: Please describe the communication between the health care provider and women after they come back from emergency caesarean section.

Participant: so after they come back from theatre I would say the communication is usually delayed yeah being that when people are operating the doctor who operates on the patient remains in theatre so it is like I operate on this woman I remain in theatre the patient goes out of theatre I remain to operate on another one so it means that at the time the patient is recovering I am not there to say this is what I found this is how the operation went and this is how I want you to look after yourself so that gap is there the immediate communication to say this is what went on so what we are seeing is that usually the communication will come a bit later when

we come to do our rounds it may be later in the day or the following day when you come to explain to the patient this is what we found and this is what we want to do or this is what happened to your baby so because of these deficiencies we see nurses now because when you are operating you are with the nurses in theatre so the nurse will receive the baby so they try to fill in that gap when the doctor has remained of course the nurse will talk to the patient but the patient would want to listen to the doctor who operated on her but that doctor has remained in theatre to operate on other six women so patient satisfaction may not be there because you are getting information like from a third party, a third party is the one who says the doctor did this so the doctor may only come in after they are done with their list the following day depending on how that day is of course the expression goes on before discharge they are told what was found what the implication of the caesarean section is and when next they should conceive things like that.

Interviewer: What is the standard of communication, what is expected because you are saying there isn't that immediate communication, so what is the normal thing or how are things supposed to be?

Participant: the standard is we have two types of patients depending on the anaesthesia they are given there are those who are operated on and they are not put to sleep so they are given an injection the lower back is numbed you can talk to them so the standard is after the operation you need to talk to them as you are talking to them you involve any person they are comfortable with spouse, the mother or somebody they have come with and say this is what we found and of course that goes with state are they ready to be talked to then there are those who are put to sleep so those who are put to sleep need to make an assessment and they are able to listen to you those are the ones where the issue is at least those who are awake while you are operating on them you are moving them out of the bed you can talk to them to say we are done you will find your baby on the ward or your baby has been taken to ICU they get a bit of what is going on the issue is those who are put to sleep who by the time they are fully awake they are out of theatre and they are on the wards so the standard is as soon as they are able to listen to you, you try and allay the anxiety and tell them what the next steps are or what the issue is.

Interviewer: As we conclude this interview is there anything of importance that you would like to talk about regarding communication between health care providers and women who undergo emergency caesarean section?

Participant: I think it is an ongoing thing I think as we are trying to improve communication we also want to see how we can bridge the barrier to our population to find out how because what we have seen is a place where people say the doctors know what they are doing so most of our communities as long as the doctor said they think it is right. I have seen a bit of change in those who are elite tend to ask questions doctor I saw this on google I went through this please explain to me we are seeing this especially in the private and those who are elite yeah with higher level of education but I think most of our community we still have that *gap* where they may not want to find out what it is. Sometimes we see that they believe that a doctor knows what is best we have had patients where two years later she comes back, you ask her you went for a caesarean section why did they do a caesarean section no I was just told that I needed to go for a caesarean section did you ask why what was the indication for you to go for caesarean section no I am sure they knew what they were doing we also want to have a campaign in our women they get to understand why certain things are done because it has implications on your future productive life or on your future health. Because at times that file may not be there you were operated on at [hospital name removed] the next thing you are operated at [hospital named removed] so the doctor who will be there will not be privileged to the information which is in the file in the other centre and this is the person who can help so we also need to get a campaign over our women also to get the zeal to ask doctor why did you operate on me because sometimes doctors may be carried away thinking the other person has explained and the patient is discharged without even knowing the implication of what they are going through

Interviewer: Doctor I would like to sincerely thank you for agreeing to be interviewed at short notice.

Participant: thank you very much for the time and for the opportunity

CONVERSATIONS WITH MIDWIVES

PARTICIPANT NUMBER ONE (RM1) APPEARS NERVOUS

Interviewer: Welcome to the interview.

Participant: Thank you.

Interviewer: Please tell me, what do you like about your job?

Participant: I like everything. I enjoy everything mmm even doing deliveries, ah also attending to patients that need urgent care.

Interviewer: What is role in obstetrics as a Registered Midwife at this hospital?

Participant: my role as Registered Midwife is to assist women who are pregnant in delivery or even during antenatal yes.

Interviewer: How often do you deal with women that need emergency caesarean section?

Participant: Ok in here (Obstetrics Intensive Care Unit) I can say weekly or two weeks we encounter women who need emergency caesarean section but it is not every day because most of the patients that come in our ICU are women who are critically ill. Sometimes they need …. support, others come as postnatal mothers.

Interviewer: Describe the communication between health care providers and women who undergo emergency caesarean section before they are wheeled into theatre.

Participant: if the patient is conscious we usually communicate with them, we explain the procedure and why they need emergency caesarean section. If the patient is unconscious, if the relatives are there we consult from the relatives if they can sign the consent form. We explain to the relatives, this is what is happening and she really needs caesarean section but in case of the absence of the relatives of the patient they are unable to talk, the Head Superintendent can sign or the doctor who is attending to the patient.

Interviewer: Apart from the indication for caesarean section, what other information do you give the women who undergo emergency caesarean section?

Participant: We explain the condition that she really need caesarean section and we explain the advantages and if we are not going to do caesarean section what will happen (consequences).

Interviewer: Please describe to me the process of consenting for emergency caesarean section.

Participant: the process, anyway sometimes it depends with the condition, I will give an example if it is a raptured uterus you don't really need to take your time explaining to the patient or relatives, you have to save the life of the mother

Interviewer: Please take me through the process from the time the patient is brought into the hospital, operated upon to the time they are discharged?

Participant: like I said the women that come here (ICU) need critical care so we don't even discharge patients from here. We don't admit patients directly, they first pass through Triage, if they need to come here they come here and if the patient was in the other ward and the condition changes they are brought here. Then if they improve we send them to the other wards.

Interviewer: Describe to me the communication with the patient after she comes back from theatre.

Participant: if the patient comes back to theatre we explain what went on in theatre, in cases of maybe they did hysterectomy it is not usually us who explain but there are doctors who explain what they did in there (theatre) reason why they did what they did in theatre. We also continue giving care to the patient and also explain to the relative.

Interviewer: What sort of information is given to the women before they are discharged?

Participant: that question ok here we don't discharge patients. So most of that information is given in the wards. But we can talk about if she goes with the baby we tell her to continue breast feeding the baby. We encourage the woman to be keeping the caesarean section wound clean. She should clean it with lifebuoy soap.

Interviewer: What are some of the challenges you face when you are communicating with the women who undergo emergency caesarean section?

Participant: for me the challenges is the language barrier I don't know Nyanja that much. Sometimes we see patients from Choma (Southern Province) maybe they can be speaking in Tonga I don't know the language but others they get Bemba others they get Nyanja others don't but we always work with someone who can interpret to the patient.

Interviewer: As we conclude this interview, is there anything you would like to talk about regarding communication between health care providers and women who undergo emergency caesarean section?

Participant: Ah Not really.

Interviewer: I would like to sincerely thank you for your time.

Participant: Alright, you are welcome. Thank you.

PARTCIPANT NUMBER TWO (RNM 2) APPEARS NERVOUS

Interviewer: Welcome to the interview.

Participant: Thank you very much.

Interviewer: What is your favourite food?

Participant: Ha ha ha ha I have a lot. Too many ha ha ha.

Interviewer: What do you love most about your job?

Participant: What I love most about my job is the fulfillment at the end of it part of helping out women especially here in the ICU. It is just that satisfaction you get when a patient is leaving the ward, they are doing better than they came in.

Interviewer: What is your role in dealing with women who require emergency caesarean section?

Participant: my role is to educate these women specifically about emergency caesarean section and explain the procedure to be done on the patient, the possible outcomes and just prepare them psychologically.

Interviewer: Please describe to me the process of consenting for emergency caesarean section

Participant: before the patient is taken into theatre we have to ask for consent, if they are unable to if there is a relative available they consent and if the woman is underage the relative can also consent and if there is no relative available somebody senior or a senior medical personnel can consent on their behalf.

Interviewer: What are some of the challenges that you have encountered when communicating with the women who require emergency caesarean section?

Participant: The challenges, it is mostly language barrier by trying to explain certain things to a woman but they won't understand, maybe certain terms that you get to use to explain these procedures they don't understand actually these are challenges we get.

Interviewer: What do you normally do to mitigate this barrier?

Participant: it is better if on duty there is a person that understands the language that the mother is using yes if there is someone that understands the language that the mother is using, it is easier that person can interpret but usually you just try your best you put the bits and pieces that they can get, they can grasp. But it is usually a challenge.

Interviewer: So going forward, what would you recommend in terms of the communication between health care providers and women who undergo emergency caesarean sections?

Participant: Hmmm on that one it is quite challenging, it is quite challenging most of the time there instances where we have patients from other countries but no one knows that language so it would be easier if we have a relative that understands both languages and just be a mediator maybe it can help out. But on the language barrier I don't really know how we can fix this.

Interviewer: As we conclude the interview, is there anything of importance that you would like to talk about regarding communication between health care providers and women who undergo emergency caesarean section?

Participant: I think emphasizing on how long it should take for them to conceive again because I think we have had a lot of challenges on that one. You find a woman comes back the following year or within a short period from the previous caesarean and we all know the consequences. So I don't know if there is more emphasis that can be put communicating with them on discharge would be of great help.

Interviewer: Who usually communicates that information to them?

Participant: it is multidisciplinary yeah on discharge the midwife explains but when explaining the procedure the doctor also tells them. So I don't know if they just don't understand or I don't know what emphasis.

Interviewer: is this information communicated to them?

Participant: yes it is.

Interviewer: So what do you think could be leading them to not adhering to professional advice that you give them not to conceive within a short space of time after the operation?

Participant: I think it is to do with the social background and it depends on where the woman comes from. Some are under pressure to have more children at a young age. So I am thinking in those lines maybe others just from advice from the community others will give the experience that I got pregnant immediately after caesarean and there was no effect. So where they are coming from matters.

Interviewer: Which group of women is this common, is it among women with low levels of education or high education?

Participant: It is very common among women with low levels of education. Those are the ones we have most challenges with.

Interviewer: I would like to sincerely thank you for your time.

Participant: You are welcome.

PARTICIPANT NUMBER THREE (RM3)

Interviewer: Sister, welcome to the interview.

Participant: Thank you so much.

Interviewer: please tell me your role as a medical practitioner in dealing with women who undergo emergency caesarean section?

Participant: my role is very involving, before a woman goes for caesarean section we have to counsel the woman so that she is aware of what she is going to undergo and if there are any dangers we assess the woman so that we prevent the pre-operative complications. Then the period of medication and even involving the significant other husband so that everybody is involved in the care of the woman pre-operative and after.

Interviewer: So are you the medical personnel who is in contact with the patient throughout?

Participant: Yes, throughout before the caesarean section, during the operation and even after the operation until discharge. I am able to communicate with the patient, the significant others and other people from theatre.

Interviewer: please describe the communication between health care providers and the women who undergo emergency caesarean section before they are taken into theatre

Participant: the communication which is there is that I have to explain to the woman what she will undergo through and any if there any dangers I will tell the woman the procedure will be like this and this and the outcome might be like this depending on the condition. Then after communicating with the woman if there any issues normally they are a bit scared most of them are a bit scared when going for caesarean section so they ask a lot of questions, sister I am going to come back, is my baby going to be ok, can you involve my relatives that I am going for caesarean section sometimes they even refuse to sign so you have to tell them that in cases where the condition is life threatening you tell them I will not wait for people who are not here to sign if it is an emergency I will involve people from administration to sign if I can wait we will wait for the relatives to come and sign until when the patient is fully aware and the relatives sign that is when we take them for caesarean section.

Interviewer: Apart from telling them the indication for caesarean section and the consequences of not going ahead with the operation, what other information do you give them before they are taken into theatre?

Participant: Most of them I explain to them the possible outcomes and even what they should do before and after caeser. Before caesarean section we explain to them to say we will starve you may be for twelve hours, you need to make sure that you are clean and any variables removed so that when she goes there (theatre) she is ok. Then we explain to them to say even after caesarean section there is pain but you need to ambulate early to reduce the risk of deep vein thrombosis and even healing early even how to clean the wound even after cesarean section even when they go home even when they come for review and for the rest of their lives I explain to them.

Interviewer: Kindly describe to me the process of consenting to emergency caesarean section

Participant: For emergency caesarean section there are two things involved if the person going for caesarean section is below 18, normally those with emergencies they come with relatives so there and then we will make the relative sign. If the patient has come with an ambulance and the relatives have remained we take to the administration the Head of Department to sign for them on behalf of the relative but if is an emergency and she is above 18 and able to write they sign. So there are three people it is either the relative, themselves and us medical personnel for emergencies.

Interviewer: What are some of the challenges that you face when communicating with these women who require emergency caesarean section?

Participant: the challenges we have when communicating with the women is that others have a preconceived mind that people die in theatre. So they would say no I am waiting for my father to come and sign even when their husband is there, they will say no this one is not my father suppose I die so you find that even when you are explaining an emergency a patient will not understand and sometimes they come with in laws, as Africans we take it to say in laws are not so much related so they will say I don't want my in laws to sign I want my father to sign. So in such cases we really have to counsel the patient to say if we don't do this (operation) its either you or the baby will die. Sometimes when we reach that stage that is when they say sister let my in laws sign so that I save my baby and myself. So those are the challenges we find because people are scared.

Interviewer: Apart from that what else do tell the women?

Participant: Apart from that even the relatives and the patients are even scared of the procedure itself because of maybe what they went through it sometime back, so they just don't want they think I will die yes.

Interviewer: Since it is an emergency, how much time do you have to explain to women who require emergency caesarean section?

Participant: In emergency situations I think time does matters, there are times when we explain as we are going to theatre. The moment we receive the patient we know that this is a life threatening condition normally it is not just one person, this one is doing the preliminaries for theatre then the other is talking to theatre, sometimes we even counsel the patient while on

the operating time. Sometimes we really have no time, we want to save the mother and the baby, so sometimes, time is not specific it just depends with the condition but where the patient is stable it can even take ten to twenty minutes for them to understand to the relatives and we say for now we are not waiting for anyone we are taking you to theatre. But there are certain conditions where you know that if you wait for ten minutes this woman is going to die, we don't even waste time, the rest of the things we finish them while we are in theatre.

Interviewer: Describe the communication between health care providers and women after theatre?

Participant: ok when they come back from theatre, these days we do spinal anaesthesia so they are fully awake from theatre they are able to say like here they say sister where is my baby, most of them that is the first thing they ask. The moment they know they are fine what will come into their mind is sister where is my baby. Is my baby ok. Depending on the outcome we are able to explain there are those with hypertension if the baby died we take time to explain there and then we would want to make sure they settle, you control the blood pressure but to the relatives when they come we tell them that the condition is like this we have lost the baby but we needed to save the mother. But for now we haven't disclosed to the mother when she delivered. When all is done we counsel them together and tell the patient to say the surgery was like this but unfortunately we lost the baby. But where both mother and baby are ok we tell them there and then we are bringing you the baby it is still in theatre we are taking you to the ward you and your baby will be together, we will take care of you until discharge.

Interviewer: What kind of information do you give to the mothers when they come back from theatre?

Participant: when they come back from theatre we talk to them about the importance of medication, that they will be given medication sometimes they complain it is paining but we explain to them that it is for your own benefit. We also talk to them about coming out of bed early because it also helps in healing so we explain to them that after eight hours you are supposed to sit and we even say from now you are not going to eat anything we will give you food from the drip and medication until maybe after six to eight hours that is when we will because sometimes they just come from theatre sister I want to eat nshima (maize meal) so you

will explain to them to say for now you are not supposed to eat such kind of food until further notice.

Interviewer: When is the information given to them?

Participant: It is given before, we start before, we explain to them when you come from theatre things will be like this, you may not be able to eat nshima and solid foods we will just keep you on drip after that you will start the oral sips and later you will eat the nshima and the rice.

Interviewer: What is the standard of communication for this hospital at the moment for women who undergo emergency caesarean section?

Participant: for now I think the challenges are being reduced because from antenatal we explain to them to say when you are pregnant anything can happen so we prepare them from antenatal so you find that even when they come you tell them to say you are going to theatre most of them are aware. Sometime back we had a lot of challenges others even used to die because they refused to go to theatre. But for now I think the challenges are being reduced through the public health education that are done in the community and antenatally such as that even when they come here they will say sister I know that this is my third caesarean section I know this. Unless those who have not been going for antenatal that is where we have challenges. But I think it is better for now.

Interviewer: What kind of information do you give pregnant women during antenatal classes?

Participant: The moment someone conceives we tell them to come to the hospital. What we start with are the dangers of pregnancy, what can endanger their life when they are pregnant, the baby may die even issues of eclampsia which have become so common these days, which is killing both the mother and baby. So we explain to them about the signs and symptoms so that when they have those signs they should rush to the hospital. Apart from that we also explain preparedness, what they need to buy for the baby and for themselves and even those who stay far away from the hospital we tell them to prepare money in case it is in the night and the husband is not there, if something happens they are able to book a taxi even just to reach a hospital or a facility. So we talk to them also how to take care of the baby when they deliver, how to take care of themselves after they deliver. If it is a caesarean section we tell them how

you are going to keep yourself is like this and this. We explain to them about the importance of them taking medication when they are pregnant, medications like …. Boost their blood, we also give them fancida for malaria prophylaxis.

Interviewer: What else?

Participant: Even the signs of labour. Some time back the women would come when they have already delivered and when you ask why didn't you come I didn't know I was in labour. So we explain to them that if you see signs like this water starts coming out don't stay because when you are pregnant the baby can come out anytime. If you see you are bleeding please rush to the hospital and by doing so we have saved most of the mothers who deliver from here. Sometime back it used to be half of the hospital delivery half home deliveries but now we have 95 percent hospital deliveries. They will come and say sister I am feeling like this I don't know if I am in labour so we are able to examine them. If she is not in labour we will tell her no maybe it is just another infection then we let them go home. We also explain to them that when someone is pregnant the immune system is low so they are prone to urinary infections because their cervix is a bit shortened and the communal toilets we use even here at the hospital it is easy for them to get an infection so when they feel itchy we tell them to come to the hospital and not to use herbal medicine which might be a danger to the baby and themselves.

Interviewer: Some of the medical personnel mentioned language barrier being a hindrance to fully communicating with the woman who is required to undergo emergency caesarean section, is this an issue with you?

Participant: in very rare cases I am saying so because like here in Lusaka Nyanja and Bemba it is so common such that there are very rare cases where you have difficulties unless non Zambians. A bit can pick a bit of some Nyanja but others it is quite difficult unless maybe if they have come with their relative. We are unable to try that but most of the time for none Zambians that is where we find it difficult. But for Zambians if it is there it is about two to four percent, it is not much.

Interviewer: is there anything else in line with communication you would want to talk about as we wrap up this interview?

Participant: what I can say is that communication is very important because in life we take it for granted that when I take this woman to theatre she will come back with the baby. Now if the patient does not come back should anything happen the relatives will come back and say no you didn't explain this if you had said this we wouldn't have agreed now look we have lost the mother we have lost the baby. They know even the consequences that this woman can survive or may not it is 50 – 50 as a medical personnel I know that this woman may survive or not so it is important to work together for not just in terms of death but even for total care because even when they go home that care should continue . so if you give enough information they will be able to care for themselves when they are in the hospital if they are any dangers they will be able to come back sister explain to me I am experiencing this and this that is why I have come back but if we start hiding some information to say maybe it is not necessary for them it will be difficult for them to make decisions even when they are home. I will not be there but if they have that information they will come back for me I think communication is very important.

Interviewer: In your opinion, what do you think is lacking in terms of communication between the two parties?

Participant: what is lacking on the medical side is that sometimes we are understaffed, I have five patients to take to theatre. I might not take all that time to finish explaining to them but where I needed to do so I may not manage depending on how the situation is because we are understaffed [text removed] you find that there is only one nurse in the whole ward you take care of many patients maybe five of them are going to theatre it becomes a challenge for me to give the total information to a patient. Then even on the patients themselves some are so illiterate such that when you explain even traditionally what they know is that when you do this you will die even when you explain to them because when they were growing they were told that if you deliver through caesarean you will die those who deliver at the hospital die. I remember we had a woman from [location removed] who refused to be put on oxygen said her father told her that oxygen kills. She was desaturating somewhere 80 and [text removed] she completely refused until when she became worse that is when we managed to put her on oxygen against her will. But thank God she pulled through. So those are some of the challenges

we find because of preconceived information it becomes difficult to change them especially those who have never been to school. But those who are learning it is a bit easy.

Interviewer: What do you recommend be done?

Participant: I think sensitization both at community and hospital. Public health care nurses should be able to sit with people in the community and talk to them. We just need to impart knowledge in the community.

Interviewer: sister thank you so much for your time.

Participant: you are welcome.

PARTICIPANT NUMBER FOUR (RM4)

Interviewer: Sister welcome to the interview.

Participant: Thank you so much

Interviewer: please tell me, what role do you play when you are dealing with the women that require emergency caesarean section?

Participant: my role as a midwife when dealing with women that require emergency caesarean section much as it is an emergency we need to give psychological care to these women it is something that they didn't expect so worrisome. So we give the psychological care simultaneously with preparing since it is an emergency we don't have much time to sit and liaise with the patient so we will be preparing them for theatre while giving psychological care reassuring the woman what will happen and the outcome.

Interviewer: please take me through the process of what happens when the woman who requires emergency caesarean section is brought into the hospital, taken to theatre, after theatre until she is discharged.

Participant: I will talk of a woman who is bleeding in pregnancy and is brought as an emergency. So it is not normal for the woman to be bleeding when they are pregnant and we call that antepartum hemorrhage when that woman comes we will need a team of people, people will need to team up because you cannot attend to that emergency as an individual so this woman will need to be … we will need to replace the lost fluids, we establish the cause of

the bleeding, then we prepare going for theatre, preparation on the body's mother, we put up the cannula, the fluids we catheterize because when the woman is taken for operation we don't want a full bladder or else it can be injured so then we also collect blood for cross matching we want to know the blood group for the woman so that we start purchasing blood to replace the ones that she is losing and then we want to establish the hemoglobin levels she has. So we would want to continue monitoring fetal heart because we are dealing with two lives here we don't want to lose the baby neither to lose the mother so we will continue monitoring the mother carrying out the observations how is the bp and we also listen to the fetal heart so basically we want the wellbeing of the mother and baby. And after theatre we called that the woman is ready actually we escort these women they don't just go after preparing the trolley we come from theatre we escort these women upto theatre there we are expected to receive the baby, we will resuscitate the baby according how hard or the outcome of the baby we resuscitate the baby to make sure the condition is satisfactory. Then depending on if the operation delayed somehow and the baby has difficulties in breathing or initiating the breathing we take the baby to the neonatal care unit for further management or if the baby is ok we come back with the baby and take care of the baby. There in theatre the mother will be kept, the only problem we have so many cases so usually they are not observed for a long time, they will call for us to go and collect the woman when we get there we get the baseline observation after theatre, we bring them here (ward) we continue observing the vital signs and also checking on the wound if there will be any bleeding even also the vagina there are some who can be bleeding if the sutures were done inside. Then we also when we put them in bed we give them medications which will be antibiotics, …. Pain relief and the iv fluids because we do not want them to be dehydrated since they are not eating there and then.

Interviewer: That sound like a lot of things to do, how much time is required when you are dealing with an emergency case?

Participant: hmmm dealing with an emergency case, a lot of time is needed and you can imagine you are understaffed and of course that won't be the only patient you would be attending to definitely there will be others because we also keep patients with bp, eclampsia even fitting so you find that you don't really meet the expectations of that woman. When there is an emergency, it is really involving, it is quite involving yes.

Interviewer: How would you describe the communication between medical personnel and the woman who requires emergency caesarean section before they are taken to theatre?

Participant: the communication depends on an individual ok there are some women who are good to engage in the discussion and we will be communicating and all will go on well. There are some whereby they will not even see the care you are offering them they want things the way they feel it should be done to them apparently you just have to be tolerant, there are some who would even say things that are ha ha that are not palatable so to say but you just have to be patient because if you are caring for that woman putting yourself in their shoes you wouldn't mind what they are saying. But most of the times the communication is good especially when you are in the low cost wards, these women they are very easy to engage in a discussion and they are very obedient usually we don't have so many challenges with them.

Interviewer: And the high cost wards?

Participant: I remember the time I worked there, it is not everybody who is difficult, they really give respect to the staff but there are some who would not take it so lightly they would think you are not concerned and you know emergencies here let's say I have received an emergency it won't be the only emergency in [hospital name removed], there will be other emergencies from other points and we have just one theatre so there are times when this woman is delayed being taken there and they think we are just sited because we don't want to take them there, not knowing that the theatre is busy with the emergencies you know the other emergencies you have a woman who is bleeding and is fitting that is an emergency also we want to save both. We also have women with fetal distress where the baby is tired, not breathing so much or the breathing is high, the baby is distressed so we just have to remove that baby. So when you delay somehow to take that one, they will think you are just deliberately delaying in taking them to theatre. So you would try to explain to them but they can't accept it there and then so they will say no if I lose my baby it will be your fault, anyway there are times when it is rough.

Interviewer: Why do you think some of the patients are difficult to deal with?

Participant: Some of the patients are difficult to deal with probably because of the previous experience they had, they are some maybe who were not treated well, sometimes it can be

genuine or maybe the perception of how they were treated the previous time they perceive it like they were neglected meanwhile maybe it wasn't really explained to them how it happened. For some it is just their personalities there are just people it is me they are in the me first world not tolerate to wait for some people just these hearsay stories that when you go there they don't treat you well so they come with a lot of preconceived mind.

Interviewer: who is the first person the women who undergoes emergency caesarean section have contact with?

Participant: it is the nurses.

Interviewer: the nurses or the midwives?

Participant: ok in this department yeah we have few nurses who are not midwives but those mostly are in the gynae ward but obstetrics its midwives because sometimes we find these women to be difficult maybe because of the reception where they are coming from because the first people to receive them are the clerks or the cashier at the main reception where they are registering to come. So there are times when they will meet those challenges exchange words with those people they are coming to meet us when they have already been offended by those people. So they tend to take it but it is now upto us to read the patient and just calm them down take them as an individual because every patient is unique, you can't treat the patient like the previous one you saw every patient is unique, you tend to establish why they are behaving like that if you get the original their agitated or rude so you get alone together. There are times when we have had these patients they come they were offended somewhere they were not treated well you take them in your hands and treat them so nicely in the end they will open up to say I never knew there were people who are good, they make you relax because where I came from this is how I was treated so even when I was coming I expected the same treatment to continue for some they even apologise to say I apologise for the way I behaved when I was coming.

Interviewer: What kind of information do you give the women before they are taken into theatre?

Participant: the kind of information we give them before they go in theatre you know there are people who have never been in theatre they will be apprehensive, they will be anxious, so

you tell them of the expectation when they go to theatre, what they will find in the group of people that will be there those that will be working as a team, the betterment of them and their babies, we even tell them the effects of the drugs and reassure them to say much as we are doing these things we cannot say they are 100 percent you make them understand that there are advantages and disadvantages, because even just a normal delivery there are disadvantages somebody can end up bleeding and it takes them (die). So we just reassure them of expectations because it is better the health talk is given before they go in theatre it will be easy for them to remember those things unlike when they come back from theatre they are so much in pain even if you will tell them things they are supposed to do it will be very difficult for them to comprehend because they are in pain all they want is that pain to be relieved. So that is how come it is better to tell them when they are going to theatre that they will be able to see the baby when they are there then afterwards this is what you are expected your legs will be numb, we will make you to lay on the bed for a certain number of hours that is when you will start ambulating bit by bit and all the expectations we will be telling you as you go.

Interviewer: you mentioned that it is better to give the health talk before the woman is taken into theatre, aren't they anxious before?

Participant: they are anxious but it is easy for them if you had said it before then you come and reinforce afterwards you say you remember when you were going (theatre) I had told you so this is the time when to make you say lie in this position without you turning it is like this. They are quite anxious that is why we need to say it both times because they may not remember the things you told them before.

Interviewer: Who gives the health talk?

Participant: it is us the midwives.

Interviewer: describe the process for consenting to emergency caesarean section

Participant: the process for consenting to emergency caesarean section depends on the age of the woman. If they are under age we involve management they are the ones who sign the consent for them if there is no adult that has accompanied them because these emergencies can be brought from other facilities with an ambulance and relatives have remained there if they are unable to make those people sign. But the practice, the teaching has been whenever you

are dealing with an underage we have to get the consent way before we anticipate anything that of an emergency but there are times when maybe this woman just went with neighbours those cannot consent for them and if they are able to *sometimes they come in a state where they cannot even give the consent* sometimes even if they can consent on their behalf looking at the situation maybe somebody is already ..still management will take it up. But for those that can be able to understand when you explain to them we can read the consent to them and tell them if they understand they can sign

Interviewer: Who gives them the consent form?

Participant: It is us, the midwives, the consent usually when we give it the doctors after tracking the patient they would have come up with a plan to say this one is an emergency ok sometimes we don't really need to be told even us we know that this is an emergency even before the doctors see because we see the patients before the doctors come and make their own further plan sometimes it will just be a continuation of what we have already come up with. So during that period we can already make them consent before they are seen by the doctors because we already know where it will end yeah we explain the procedure for someone who comes antepartum hemorrhage we know that that one it will be straight away be an operation. Even before the condition we have them consent in advance.

Interviewer: So what is the standard of communication with these women who require emergency caesarean section?

Participant: the standard of communication I would say ha ha ah ok what do you mean by the standard of communication?

Interviewer: What I mean is what is expected of you to communicate to the woman that requires emergency caesarean section?

Participant: the standard of communication is the same be it amongst the doctors or nurses, they are things whereby we meet even them they have that teaching on how we are supposed to approach an emergency eh there are times whereby if you are the one who is found with that emergency you have to call for help that is the standard for approaching an emergency. If a woman walks in and she is bleeding I have to scream loud to be heard by everyone and when you are calling for help you have to specify what kind of help you want and what emergency

is there say if the woman is bleeding before they deliver I will call for help to say I have antepartum hemorrhage there so everyone as they will becoming they will know the condition they are going to attend to and it involves all the cadres in the ward maids inclusive porters also and the person who has identified that emergency you are leading the team. You are the one who is going to delegate to who and what you want to be done or eh mention names if you are saying things [name removed] do vitals on this woman and record, you have to be very specific and you expect to have feedback from the person you told to do things, they have to get back to you or you want me take observations which she does then she will communicate to me, this is like this, then the next person I will tell even if I am the midwife who has identified the emergency even the doctors I will have to tell them what to do, lets collect blood they maybe porters send them to take blood to the lab or blood bank and collect the blood, keep the patient warm just like that depending on the number of people that we have.

Interviewer: I know earlier you mentioned prejudice or women coming with a preconceived mind to hospital, what other challenges do you face in communicating with the women who require emergency caesarean section?

Participant: another challenge I would say is the lack of knowledge on the conditions because there are some women whereby even if it is an emergency you are communicating to them they are not taking it serious, they will not even like respond and do the things the way they are supposed to be done and for some just that attitude of ah they just want to do an experiment on me so some of those challenges are the ones that we meet, the lack of knowledge and just the attitude of the patients.

Interviewer: What else?

Participant: hmmm sometimes it could be language barrier also when we are communicating sometimes you can be carried away thinking they are getting what you are communicating meanwhile they haven't gotten anything because this is a referral hospital we don't expect every woman to understand Nyanja, Bemba so there are some people where you are communicating to them in Nyanja they will just be looking at you, this woman is Lozi she can't pick anything so like that again we find it a challenge and there are times sometimes you are in an emergency you are dealing with a person who is dumb or deaf they will just be looking

at you, you will be screaming they are not hearing you so these things we need to establish are they listening or can they talk back .

Interviewer: So what have you done to address these communication barriers?

Participant: for communication that is why even hmmm when we are at school we are told to at least know the basic languages that are used mostly depending in the area where you are at least you are supposed to know some bit of the language so that you are able to know or where you have someone who understands who talks that language fluently so that you don't lose the woman anywhere so that she can get the information and for the deaf and the dumb you use pen and paper you draw things but not in an emergency anyway but in an emergency for the deaf and dumb we try a bit of sign language at least somebody will get something. [Text removed].

Interviewer: Kindly describe the communication between you and the women after they come back from theatre?

Participant: ok these women when they come back from theatre we put them in bed we tell them to say we have put you in bed starting from this hour about six hours you just lie in bed on your back but it is a challenge if you you slept or are laying on your back it is a challenge with back pain but there are times you know our beds are old the matresses are finished so you even feel for these women when you are telling them to lay on their back you tell them they can put a small pillow so that there is no much aching but it is not always easy when you tell them that they should be in that position for a long time and we also tell them they will continue on the iv fluid they are not supposed to eat anything though nowadays they don't do the general anaesthesia they just do spinal but still there are some people who react to the spinal that is why we don't want them to eat anything so that they don't start vomiting and you know with the vomiting it puts a strain on the wound. So we also tell them we are going to give you some drugs that are going to relax you you should rest and we don't give them their babies just there and then because might start crying, they cannot turn and feed the baby so we take care of their babies we feed them and we keep where it is warm and make sure we lock only staff have access to the babies because in the past we used to lose babies. This woman you have given her anaesthesia she may just fall asleep and you never know who walks in and you don't know what they would do to their babies.

Interviewer: As we conclude this interview is there anything of importance that you would like to talk about regarding communication between health care providers and women who undergo emergency caesarean section?

Participant: the only thing I would want to happen sometimes the communication why it is not like fully given and the way it is supposed to be given maybe it is because of having being short staffed there are people whereby you have other things to attend to so you don't find all that time to dedicate it to this woman and give her the full informed eh message that you want to deliver to this woman and there some whereby they are so stressed some people don't know how to handle their stress so sometimes they will not even communicate anything so the woman will just be asking and the more the woman is asking the other cadre will feel like they are being troubled or this woman is just troubling them but it is because the information hasn't been given in totality to her so the women will continue asking. So I think the shortage of staff makes us not to give proper communication to the woman. And also I just want to encourage our fellow staff just to be good amidst the stress because this thing is here to stay with us any way. It is not ending now just need to have empathy that is why even the students who come here I continue preaching to them be empathetic at least take a few minutes be good listeners because you don't lose anything by delivering the information than you would if you leave the woman without information they will keep pestering you because they are not satisfied because they would want to hear more and sometimes even us ha ha we are human we error, there some people who are just impatient.

Interviewer: you mentioned that in an emergency, there is limited time, when do you think would be the best time to properly communicate to women who require emergency caesarean section?

Participant: even antenatally we teach these women the emergency preparedness, we also teach them the danger signs in pregnancy, we also teach them on the danger signs even postnatally we go beyond that. So proper teaching of these women is antenatally if it were possible to do it even before they conceive so that during that time at least they have all time to listen and they can remember unlike during the time when an emergency has happened so we will do well to encourage these women to attend antenatal the moment they discover that they have conceived until the time that they are going to deliver that an emergency can come

at any time it does wait it can be either six months eight months or whatever so at antenatal classes we need to up our game so that these women are prepared to be less risk like trying to educate a woman in an emergency because it is not really practical that we can give proper information to these women. So even as they learn antenatally they will not delay to come when an emergency occurs there they will know to say hmmm this thing and they will come early and seek for medical attention very early before it is too late.

Interviewer: Sister, I would like to sincerely thank you for your time.

Participant: Thank you so much [interviewer name removed].

Interviewer: Thank you.

PARTCIPANT NUMBER FIVE [RM5]

Interviewer: **Sir,** welcome to the interview.

Participant: Thank you [interviewer name removed].

Interviewer: Tell me what is your role in dealing with obstetrics in particular emergency caesarean section cases?

Participant: so like in a department where I am where from which is Obstetric TRIAGE, most of these women will come in serious obstetric conditions and as such they would require quick surgical intervention so that we save mother and baby or we save the mother. Yes so they will need quick intervention. So most of them because of the nature of the condition they will be able to sign the consent form to surgery but some because of the condition they will be in they will not be able to sign the consent and in such a case the medical officers will sign for her before they take her to surgery. So yeah that is usually what happens and those that seemingly come in a stable condition we would want to explain the procedure to them then they will be able to consent to the procedure.

Interviewer: Please describe to me the communication between health care providers and women who undergo emergency caesarean section before they are taken to theatre.

Participant: ok so the communication basically has to involve explaining the need for the operation, you explain to them that in case you delay (in consenting) the condition will worsen

and the baby may die and as such that you make them understand the consequences of them not signing the consent form in time because like I said earlier it is EMERGENCY TRIAGE because most of them come in when they are fitting or have got very high blood pressure and such keeping them may mean the blood pressure will continue rising and as such they may lose the baby. So those are the details we explain to the mother.

Interviewer: please describe the process of consenting to emergency caesarean section.

Participant: basically what happens like I said you have to explain to them ah what is going to happen and also a detailed explanation in terms of the nature of the operation itself how it is going to take place? Being that it is an emergency there will be nothing like the mother starving prior to the operation, we also give them explanation on the anaesthesia we are going to give, because there are two types, there is general and spinal one. For spinal they don't go to sleep it is from the waist going downwards and as such we quickly extract the baby. General anaesthesia is the one where they have to sleep so based on that others will say me I prefer general others will say I prefer spinal anaesthesia. But the most used is spinal anaesthesia you say ok fine usually it helps allay anxiety they could have had before the operation and as such they are able to understand and consent. Sometimes we get cases you culturally we get women who have strong belief that the husband should consent and sometimes it also delays us in getting the consent because we have to call the relatives and they have to come and give consent. Then despite that we still explain the dangers if they take long to come. Others will also be hesitant they could be an age to consent but then you know this thing of getting consent from parents they would want to wait so that parents can come over and give consent for the operation yes. So basically that is what happens.

Interviewer: What are the challenges that you face in communicating with the women who require emergency caesarean section?

Participant: the challenges like for emergency obstetric cases most of them would be either the mother is not in a stable mental state so you would be explaining but they would not be able to comprehend what you are saying. Sometimes it is them having certain thoughts at the back of their mind and so they will be hesitant to consent. Others will be hesitant because they will be concerned with the survival of the baby. So those will be some of the things we face. Another constraint that is there is the underage you know legally patients can only consent when they

are eighteen years and above, of the cases that we have we have patients as young as 14 years old and in this case it requires consent to take the mother to theatre and age does not allow yeah so in this case three doctors can sign or the Chief Nursing Officer or the Principal Nursing Officer can sign consent. So further when parents come we explain that it was done in the interest of the patient.

Interviewer: please describe the communication between health care providers and the women after they come back from theatre.

Participant: the communication between health care providers and the women after they come back from theatre. Actually what usually happens some will not be very mentally stable but then they have given consent for the operation to go ahead so most of them would be in a drowsy state so it is important to explain to them what transpired and also what the procedure was all about. This helps them to know what took place and also being that they are nursed in the same ward postnatal and antenatal mothers it also helps in explaining to those who are reluctant to go to theatre having gone through it some of them have proved to be helpful in explaining to others what the procedure is about and as such it has proved very helpful. Usually depending on the outcome some help but others come out negative because they feel that maybe the procedure was done in a hurry so they feel they were not given enough time to understand what the procedure was all about.

Interviewer: Who gives the consent form to the woman?

Participant: the consent form is given by the midwives before the patient goes to theatre. So before they append their signatures to go to theatre it has to be explained to them about what the procedure is all about yes and so before they sign. That is why you find others are hesitant to sign because they feel like it is something big and they can’t do it on their own they would want their relatives to come have a discussion and allow them to sign.

Interviewer: so are they given the consent form to read on their own or it is read out to them?

Participant: it is read out to them because sometimes they may not understand the language and again they may not be familiar with the procedures that take place. It is read to them and they will be able to consent once they ask they will be able to sign. But in an event that you know when one is given drugs they will feel a bit drowsy and as such they will not be able to

comprehend a lot of things so all they want is to do away with what is going on and they consent. So no wonder it is very important that the procedure has to be explained to them again so they know what they consented for and what transpired in the operation so that they know this is what took place and this is what has happened.

Interviewer: So that is the standard of communication?

Participant: yes, that is the standard of communication though you know sometimes emergencies usually give us like *you have to make some short cuts,* of course short cuts meaning that you have to hurry taking the patient to theatre and as such certain details will take a bit of time to explain to them so you would want to rush them to theatre to save the baby.

Interviewer: What information do you give the women after they come back from theatre?

Participant: the information they are given is basically about the procedure, why there was that need to have taken theatre at that point in time and also we explain to them about what they sign it becomes the legal document so that in case of anything goes wrong you say we didn't do the procedure without your consent, there was consent. Sometimes also you know surgery is not one hundred percent safe, something can go wrong but in the event that something goes wrong if a person want to sue you say this is the legal consent you gave us.

Interviewer: legal backing for the patient or health care worker?

Participant: for the institution because it is a legal document for the institution yes.

Interviewer: As we wrap up this interview, is there anything else you would want to talk about regarding communication between health care providers and the women who undergo emergency caesarean section?

Participant: of importance is some of these women come like in [hospital name removed] these are patients who are referred from far facilities out there so what is important is some of the patients we get are underage and as such explanation sometimes is not given and you know as regards to an ambulance bringing a patient, they will just be limited people and consent is not obtained from relatives at the facility before they come it becomes a bit of a delay when it comes to consent so it would be very good if antenatally some of the things about consent are made known to the women during pregnancy. I think it is something that needs to be included

in the antenatal care. So it would be important if it is included as a topic so that parents even the mothers themselves get to understand that this consent at some point maybe required during the process of giving birth so that there is this document that needs to be signed and know its importance. It would be very important if this is done. Another important thing there is need to harmonise there should be a standard consent obtained at [hospital name removed], it should be the same at [facility name removed] in such a way that if parents are remaining in [hospital name removed] they can sign the consent to allow personnel at [hospital name removed] to just go ahead the procedure because they would have already consented where the woman is coming from. I said earlier it important that the consent form is included at antenatal so that the women know that there is this document that needs to be signed for emergency surgery.

Interviewer: I understand that you offer antenatal sessions here at [hospital name removed], what kind of information to you give pregnant women?

Participant: mostly the common ones that are given about danger signs in pregnancy, we also give information about birth preparedness in terms of clothing for the baby a bit of finances and in terms of also their preparedness as a mother all those things and also they are also talked to in terms of diet and also the importance of them testing for HIV, and the new one which has been introduced is the covid testing. Yeah it has also been included during the antenatal care.

Interviewer: Sir, thank you so much for your time.

Participant: you are welcome, you are welcome.

CONVERSATIONS WITH REGISTRARS

PARTICIPANT NUMBER ONE [RR1]

Interviewer: Doctor, welcome to the interview.

Participant: Thank you.

Interviewer: What is your role when dealing with women that require emergency caesarean section?

Participant: so my role is assess and see the eligibility of the patient’s need for surgical intervention primarily caesarean section and also most of the time I also participate in the actual

operation. But before that we talk about the preparation of the patient including investigations and also explaining to the patient the plan that we have for her and getting permission from her to proceed.

Interviewer: At what point do you get in contact with the patient when they are brought here for emergency caesarean section?

Participant: ah so it is basically in the emergency room that we call Triage most of the time that is where we have first contact with them then there are those that may have been undergoing what was anticipated to be normal labour and then the circumstances changed depending on their medical condition then they need caesarean section. So those the contact will be in labour ward.

Interviewer: Please describe the communication between health care providers and the women who require emergency caesarean section before they are taken into theatre.

Participant: so the communication is basically the language that a patient understands and we explain to them what is the underlying condition has necessitated or given rise to us needing to intervene with a caesarean section. The communication involves explaining the underlying condition, the dangers if we don't do a caesarean section that is the risks that are involved and the chances of having a good outcome and what is likely to be the prognosis. What are the implications on the woman herself, what are the implications on the baby to be born if we do not do the caesarean section.

Interviewer: Apart from the indication for emergency caesarean section and the consequences of not conducting the surgical procedure, what else do you tell these women?

Participant: depending on the state of the woman we also explain to them the implications of having a major operation at the point we are going to have it done on her in terms of her future fertility, what are the prospects for her to have more children in future, what are the chances that she can deliver normally, what are the chances that she will have complications in future so we discuss all those things with the woman as she is going to the operation room**.**

Interviewer: please describe the process of consenting to emergency caesarean section.

Participant: ah so the process for consenting to emergency caesarean section, first of all ah having assessed the patient, having identified the problem ah we try to see how ah is the woman in a state where she understands where she can also communicate freely because an emergency operation usually we don't have a lot of time and sometimes you find a patient wasn't even told about the possibilities of a complication during labour, so those are the patients when you start the discussions sometimes they meet the information with shock you have to explain to them gently, they might not consent you give them time and there are those who require family support we will bring in a member of the family that they trust and sit in and also participate in the discussion as well so basically we take them through the steps of explaining the condition to them trying to verify if they have understood what we have explained to them and making sure that they know the options they have and implications to each of the choices they make and once they have fully understood they will sign the consent form to authorize us to proceed with the operation.

Interviewer: What has been your assessment, do these women really understand your explanations?

Participant: Ah well it's a mixed kind of scenario, most of them do understand of course they do understand and some of them this being a tertiary hospital they come in as referrals yeah just the whole experience of having have been in labour the other side having being told something where they are coming from being put in an ambulance that kind adds a lot of anxiety to them when they come here you try to explain ok the caesarean section just the whole process of having been ferried around, from one town to another from one district to another yeah or within [name removed] from one hospital to another kinds of puts them in a situation where they think they are in grave danger so we try as much as possible not to take advantage of the situation. A situation where someone is scared and they think surgery is the ultimate solution we try to ensure that they take in the information as it is, I have come from far and this is the solution, we need them to understand even if some information has been given to them we still have to go through our standard procedure to make that they understand.

Interviewer: What are some of the difficulties that you face when communicating with these women who need emergency caesarean section?

Participant: Ah most of them are distressed, they are anxious and they are looking upto you as a doctor most of the time before you even finish explaining they say no just take me for an operation but we still have to explain fully but there are those that fail to understand and they still believe that there is always an alternative to an operation in some cases of course there is an alternative sometimes its gets difficult to get to them to say if we don’t do an operation these are possible consequences which are not like they will certainly happen if they don’t happen well and good if they happen then it becomes a very serious situation so the small number where during the antenatal reviews visits they were not prepared to say labour doesn’t always go smoothly they will be a point where if things go wrong you might need surgery they are the ones where we get a big challenge in terms of understanding the situation when they were told during antenatal to say your baby is average size on scan ah you have delivered before, this is your first pregnancy you will deliver just fine those are the ones where if problems arises it is difficult for them to understand there is a problem when previously they have been assured to say everything is fine. The other thing is the issue of consenting itself because most of these clients we are seeing they are adults who can consent for themselves so they would want the social support from the husbands because they are also worried about implications of surgery itself if they consent on their own. Some of them will feel like if things went wrong they will be blamed of having consented so this is where some of them usually would want a family member to come in sometimes a family member would sign consent for them despite being adults who are capable of consenting themselves, that is another challenge that we get.

Interviewer: kindly describe the communication between medical personnel and the women after they come back from theatre.

Participant: so after they have come back from theatre basically is ah explaining to them ah the procedure that was done sometimes there could be complications into additional steps while doing an operation you have to explain those things but most of them it is actually good outcome, it is an issue of reassuring to say the surgery went on well and this is what we found this is the outcome for the fetus, your baby is in this state and you start introducing information on how they need to take care of themselves after surgery and the kind of care after the surgery and we starting talking about the possible duration they will stay in the hospital, that is the kind of information we usually talk about.

Interviewer: As we conclude this interview is there anything of importance that you would like to talk about regarding the communication between health care providers and women who undergo emergency caesarean section?

Participant: I think I have mentioned most of the things ah yeah communication there are some situations where they will need the patient is not in a good state they might need blood transfusion and these are patients most of them just present as emergencies we have not been able to prepare them ourselves we just received them as emergencies so it gets challenging to prepare things like blood transfusion yeah especially if they have an underlying religious belief they can't receive this kind of treatment it gets challenging yeah.

Interviewer: Doctor, thank you so much for your time.

Participant: ok thank you, you are welcome.

PARTICIPANT NUMBER TWO (RR2)

Interviewer: Doctor, welcome to the interview.

Participant: thank you, thank you a lot.

Interviewer: First of all I would like to sincerely thank you for agreeing to speak with me. Please tell me the role you play in dealing with women that require emergency caesarean section.

Participant: ok thank you for this welcoming notice. So hmm here as a Registrar meeting I am post graduate following obstetric and gynae programme this is a four year programme and I am a post graduate year four. Ah in terms of caesarean sections we are dealing with the caesarean sections on a daily basis the rescue intervention for the mother and for the baby as well this is what I do, I can say in summary.

Interviewer: please describe the communication between Registrars and women that undergo emergency section before they are taken into theatre.

Participant: ok so in terms of operative delivery I am talking about caesarean sections there is some requirements meaning there is what we call an informed consent ah prior to the surgical intervention we need to talk to the pregnant woman about the benefits, the risks and just to

agree for that operative delivery ah because we are dealing with a lot of emergencies I need to admit that the informed consent sometimes is not properly processed we need to sit with the pregnant woman and explain in detail but sometimes there are some gaps in terms of explaining the possible complications for example that can arise. I need also to mention that our colleagues midwives they do a better job compared to the Registrars ah this is what I can say in short.

Interviewer: What sort of information are the women given before they are taken into theatre?

Participant: of course we need to tell them why she is going to theatre the indication because this will impact on subsequent pregnancies when she comes back she will be asked what was the reason for the previous caesarean section there are some indications they need to know because of the recurrent indications in the future subsequent pregnancies of course apart from that you tell them why the benefits of this delivery and you also need to mention it has some risks they also need to know all those consequences apart from that maybe you can tell them the type of anaesthesia they are going to receive most of them they are going for an injection at the back meaning the spinal anaesthesia you need to tell them that it has also some complications though very rare and you tell them they are not going to sleep they will be awake but they will be numb from the ambalicas going down they won't feel anything the whole process will take thirty to forty minutes maybe they are going to stay in the hospital for the next three days yes in short this can summarise what we do when we ask them to sign consent forms.

Interviewer: What are some of the challenges that you face as Registrars in communicating with women who need to undergo emergency caesarean section before and after surgery?

Participant: the challenges if I may be correct it is mostly on our side I think we need to reinforce on the practice take our time, sometimes we rush maybe there is another patient next door who is bleeding maybe there is emergency which is coming you don't take enough time maybe three to five minutes explaining to her it is an important step that needs to be ah reinforced from our part. In terms of when we are talking about patients there are some indications that are a bit difficult to be perceived by a patient based on the level of education but we try as much as possible to explain in simple language not medical terms so that she can get the information needed.

Interviewer: Are you able to give any examples?

Participant: in terms of examples hmm in terms of examples ah ok if I can pick one example this is one patient who has gone into labour and she has prolonged for example the labour has taken long and maybe apart from the labour we also intervene in terms of precipitating the labour and we put some… so that the labour can advance but sometimes that intervention can fail this is what we call a failed …..mentation. it is a bit try for the patient to understand but we explain in simple words what the failed augmentation is means meaning we have put a drip but for labour to progress maybe it has failed so you try to be a bit in a language a patient can understand.

Interviewer: Since it is an emergency, how much time is required to communicate to the woman who requires the surgical procedure?

Participant: hmm looking at the facility which a tertiary hospital it is a referral hospital all these general hospitals all these clinics they refer complicated cases but I will say the ideal time after knowing the indication that patient should be in theatre within thirty minutes but sometimes we face some challenges due to the overload of patients especially that maybe let's say this is a long weekend ah where we are remembering [text removed] but some clinics are not working proper work within this weekend so sometimes you find we are overloaded but ideally that patient should go to theatre within thirty minutes.

Interviewer: You mentioned that you have limited time in which to talk to these women and that you have work overload, when do you think would be the best time to communicate to these women?

Participant: ah the best time to communicate according to me it is from the time of indication of the caesarean section why I said there are some challenges you find maybe the doctor there are also few in terms of numbers Registrars you find people are depleted, you find one doctor is attending to more than expected patients, the WHO recommended to attend in terms of patients so when you are dealing with thirty patients and you bare alone it is a bit tricky to do a thorough job but we try by all means to meet the requirements in terms of medical attention.

Interviewer: What information do you give the women when they come back from theatre?

Participant: So as I said when they go to the ward after theatre we do daily rounds on a daily basis the surgery even if it was an eventful even after it can have some complications yes those complications are anticipated by telling you need to ambulate, you need to take small walks within a short period of time after the surgery you need to start taking some oralcepts you need to maybe lay down after the surgery if there was some complications maybe you tell them you are not allowed to eat within six hours. So all those information are emphasized after surgery we also co-manage the patients with our colleagues the midwives also they need to do their part. They also tell them to follow the instructions.

Interviewer: As we conclude this interview is there anything of importance that you would like to talk about regarding the communication between medical personnel and women who undergo emergency caesarean section?

Participant: Yes, it was my opening statement we need to reinforce in terms of communication especially that there are some patients that are coming later you find that you have done some caesarean section in 2017 you are the one who did the caeser she can even remember you she becomes pregnant again in 2020 and you are surprised oh madam you are back what was your indication for your previous caeser and you are surprised she remembers but not the way you wanted her to remember meaning that there was some lapse at some point so let us just improve our practice and communicate properly with our patients.

Interviewer: Doctor, thank you so much for your time.

Participant: Thank you for the opportunity.

PARTICIPANT NUMBER THREE (RR3)

Interviewer: Doctor, welcome to the interview

Participant: Thank you.

Interviewer: Please describe the communication between Registrars and women who undergo emergency caesarean section before they are taken into theatre.

Participant: So once we make a diagnosis requiring an emergency caesarean section, we have to get informed consent, we explain the situation yes like the mother is in danger or the baby and the imminent need for delivery through an operative procedure. So after we inform them

and they understand what the procedure will entail the complication that may arise then they endorse the signature or the thumb print before they are taken to theatre.

Interviewer: Apart from you communicating the indication for emergency caesarean section and the consequences if the woman does not accept the surgical procedure or delays, what else do you tell these women?

Participant: The other things maybe if they have any questions if they have understood, they are given chance to ask yes just that sometimes you know the situation of labour *t*hey maybe too stressed and they may not have questions to ask so they will just sign the consent form.

Interviewer: Please describe the process of consenting to emergency caesarean section.

Participant: the process of consent for emergency caesarean section hmm ah apart from that informed consent yes which is anyone above 18 years, they legally can sign the form for the operative procedure. If the woman is under the age of 18 years, there is a guardian in case of an emergency or the senior doctors on call, if the consultant is not available, the Senior Nursing staff, the night Super can sign for a patient yes. The patient would have been told already just in case they are not in a position to sign some of them are unconscious. But we don't just operate when they (women) come to us we need to be protected, the senior most doctor available or the senior most nurse has to sign.

Interviewer: Since it is an emergency, how much time do you require to communicate to a woman who needs an emergency caesarean section?

Participant: For emergency caesarean section we plan to have from the time we make a decision to the time of delivery we aim within thirty minutes if the situation allows but sometimes we might have multiple cases that may need emergency operations so we can't take all of them at once so sometimes we try to open two or three theatres just to try to avoid any complications yes hmm so basically may wait upto an hour from the time the decision was made but we try to do the operation within thirty minutes from the decision. Some of them don't even wait for example someone who is unconscious, they just go straight and then that paper work follows the patient in theatre since we are doing something in the interest of the patient even if we know that when they wake up they may sue you that I never consented for this procedure but sometimes it happens we might do it backwards yeah. .

Interviewer: What challenges do you face when communicating with women who require emergency caesarean section?

Participant: Hmm the only challenge sometimes is the women I don't know if it's because of myths they only know that they are supposed to deliver normally if they deliver by operation ha ha the society where they are coming from will start thinking that they are *not women enough* that is a challenge some of them they decline but as long as they sign against it to protect the health workers we but most of the times as long as we give informed consent yeah they should understand the implication of them refusing to go for that operation yeah after they have done that if the woman says no we can't force. Then they will sign in the file so that we are protected so that they have taken responsibility of whatever may come after that.

Interviewer: Who is responsible for obtaining consent from the women who require emergency caesarean section?

Participant: Ideally it is supposed to be issued by the one who has made the decision for emergency caesarean section, the doctor, the doctor will explain to the patient that they are going for an operation, this is what is involved but sometimes you find that we don't witness it. We don't witness it, the midwife takes care of it, they will formalize the paper work but ideally the doctor would have already told the woman that this is the situation, the witnessing of her signing, the attending midwife does it as long as the patient was told everything. The midwife may not know what is going on that is why the doctor informs the patient because they may be very busy if they are available they may even be the witness on that consent.

Interviewer: But, what is the standard?

Participant: the standard for a consent ha ha ha a consent is supposed to be issued by the one who has made a decision that the woman goes for emergency caesarean section after explaining what the procedure is the possible complications yes but sometimes the attending midwife obtains consent. The other aspect is depending on the situation these are emergency cases because there are two aspects, the operation and the aspect of anaesthesia but these emergencies an Anaesthetist will not see the women pre-operative but ideally if there is time depending on the situation we need to alert our colleagues they know also beforehand. They

may also explain to the woman if things allow because the complication may not arise from the operation itself but from the drugs that we are using.

Interviewer: What is the role of the Registrar in dealing with women that require emergency caesarean section?

Participant: The role of the Registrar, first of all ah they are the ones that make the decision to review the patient, they assess whether she can deliver normally or not. If there is need for an operation they have to make that decision and inform the patient and then the same Registrar or there will be another one in theatre is supposed to operate it may not be the same one who made the decision to operate the colleague in theatre will take the responsibility.

Interviewer: So how much contact does the Registrar have with a woman who undergoes emergency caesarean section?

Participant: hmm apart from when the patient is seen that first contact and then depending on what is going in theatre, if the patient can't go within thirty minutes we will continue reviewing, we will be assessing just to see the wellbeing of the baby as well as the mother, if something else has come otherwise it is just to review and assuring the patient.

Interviewer: Please describe the communication between health care providers and the women after they come back from theatre.

Participant: So hmm after theatre depending on what anaesthesia was used, if it was spinal anaesthesia the patient will be aware of what is going on. So afterwards usually depending on the situation usually it is very busy she will know the state of the baby. If there any things she might want to ask she can ask in the ward but the team is there after the operation they will be talking. But if it is more for the general anaesthesia she won't be able to know what is going on, whether the baby is alive, where the baby has gone, so that is where we come in the patient will be awake by the time they will be going we would have told them one or two things.

Interviewer: So what information do you give the women when they come back from theatre?

Participant: we tell them how the operation went, if they were general anaesthesia, how the baby is. By the time they are going to theatre they will know that the baby is alive, we go into

theatre hoping the baby is alive if the baby is not alive we have to break that news to the mother yeah. Sometimes we delay to disclose until they are in a stable condition.

Interviewer: You mentioned that in an emergency time is limited and you also alluded to low staffing levels, when do you think is the best time to speak with the women who require emergency caesarean section?

Participant: the best time I think it should start from antenatal, they should discuss with them antenantally so that they are ready if they can't deliver normally, if they end up having caesarean section, there is nothing like me I can't have an operation it can happen to anyone as long as they are pregnant, they will be taken for an operation if there is a complication, so a complication may arise at any stage of labour yes so it should be something that starts antenatally yeah especially I understand sometimes women are not prepared when they come for an emergency operation, you find that here we receive different women from all parts of Zambia so you never know, they may not have been told that you are going for surgery then they come they start refusing ha ha so we can't do anything.

Interviewer: As we conclude the interview is there anything of importance that you would like to talk about regarding communication between health care providers and women who undergo emergency caesarean section?

Participant: the only thing that I can say is most women may not be in a position, it may not be a good time to get consent because they are in pain, it is like they are not fully aware of what they are signing for, they may, they may start regretting but there is no other way, they are not in a position to ask a lot of questions to understand what the procedure entails and there is no one like in our setting here, ideally there should be someone to witness what their relative is being told but the set-up is where the woman is alone, so that is the challenge but it is ok this is done.

Interviewer: What is the standard for communication?

Participant: the standard of communication, what do mean exactly?

Interviewer: What is the health care provider expected to communicate to the patient who undergoes emergency caesarean section?

Participant: Apart from the operation itself there these other things, how to take care of the wound, when they are supposed to start feeding, although some of them are scared that we did something to them they want to stay a bit longer without taking anything but we encourage them to start eating as soon as possible.

Interviewer: Did I hear you correctly, you encourage them to start eating as soon as possible after the operation?

Participant: Some of them they delay mmm they may even go and come after two weeks and say I haven't started eating normally. So they are scared of the wound that they haven't healed inside. So two hours after the operation they start sipping fluids, after six hours they told to revert to their normal diet.

Interviewer: Doctor, thank you so much for your time.

Participant: You are welcome.

PARTICIPANT NUMBER FOUR (RR4)

Interviewer: Doctor, welcome to the interview

Participant: What is your role when dealing with women who require emergency caesarean section?

Participant: Ok so my role involves preparation of the patient, ok and that preparation includes consenting, giving medication and making sure that the theatre staff are ready for that patient doing the operation as well as taking care of the patient post-operative.

Interviewer: Describe the communication between health care providers and women who undergo emergency caesarean section before they are taken into theatre.

Participant: So communication is usually good but the challenge is that most of these women that undergo caesarean section you find they are in pain many at times they are distressed, even if you are explaining why they are going to theatre there are still a few that may not really get it because they just want to get rid of the pain so that you deliver them. But the communication is usually ok in that when they reach theatre they are asked on the indication, why they need to do an operation on them and they need to explain though others will say no no just do the

operation I am in pain, but we will not proceed with the operation as long as the client does not know why they have been taken to theatre.

Interviewer: Apart from the indication for emergency caesarean section, what other information do you give the women?

Participant: So when we look at consenting for an operation, there is what we call informed consent for the operation meaning that you inform the woman why you want to do the operation, what type of the operation, what are the advantages and disadvantages of that operation, disadvantages I mean the complications that may come with the operation because they need to know that it is not only the person who is doing the operation but that there are other staff in there for example someone who is going to put this woman to sleep or to make them comfortable as you are doing the operation so that so they can have complications arising from there, then you have complications arising from the operation so they need to understand all these things before they consent for an operation and what are they expectations after the operation ok because they need to know for example how long they need to stay in the hospital, what is the future of their reproductive life after the operation. So all that information has to be provided for them.

Interviewer: After they come back from theatre apart from their future reproductive life what else do you tell them?

Participant: So after theatre you tell them what you did you explain so you explain before the operation you would have a hint of what you are going to do but sometimes when you are doing the operation certain things can change so you need to communicate to them what were your findings when you did the operation, how did the operation go, was it a difficult operation or it was a straight forward operation. So all that information they need to know, for example if you planned a normal caesarean then you end up with removing the uterus so that is intra decision that you made to save that woman's life. That information has to be communicated ok and what they are expected of after the operation they need to know for example that they will not be having periods, there are sometimes that we have done an operation to remove ovaries of the eggs of a woman they also need to know the implication of that so the post-operative information has to be communicated and also the minor effects of an operation if they are in pain which pain will be acceptable of course no one likes pain but which discomfort

will be acceptable at what point do you want an intervention to be done. If they develop a headache because of spinal naesthesia those are some of the effects of either the medication that was used during the operation or if the surgeon doing the operation did some other things that would complicate that individual's life, that information has to be communicated as well.

Interviewer: Since it is an emergency, how much time do you require to communicate to a patient who require caesarean section?

Participant: So our major setting here it means from the time you make the diagnosis to the time you deliver this woman, it should be within the window of thirty minutes and you are able to provide a lot of information within that thirty minutes because you just need about ten minutes to prepare, we have a consent that is already printed so it is more like a check list that you provide but on the post-operative you need to provide the information it is not there on the check list, you have to provide extra information you have little time but within that little time the basics have to be provided, the indication of the operation, what you are going to do because for someone to consent they need to know what you are going to do, what if you say you are going to do a caesarean section but you are going to chop off their head so they need to understand what you are going to do in theatre and what is expected of them and what are the possible outcomes at least they need to know the basics. .

Interviewer: Please describe the process of consenting to emergency caesarean section.

Participant: Ok so when you make a diagnosis and this person needs an operation, a caesarean section so you explain to them first you get that consent form, you say you need to go for an operation, the reason why you need to go for an operation is this and that ok , this is what is involved in this operation ok these are the possible complications, this can happen and if you are able you can even provide the percentages in terms of risks, there you tell them how much they can occur right so once you are able to explain that information, have you understood yes because what is important is for them to understand then once they have understood you give them this form for consenting then they sign it. Then again you ask them if you are asked in theatre do you understand yes why because of this and this ok you can proceed.

Interviewer: Who reads out the consent form?

Participant: So before they go into theatre there are two things, the midwife can read the consent form to them, the doctor can read the consent form to them, there are others who may want to read on their own, they can read so you just provide clarifications, in certain areas which they are not sure of ok so they can read, the doctor or midwife.

Interviewer: What is the obtaining scenario?

Participant: the obtaining scenario, it is the midwives who get consent ok so midwives read out the form then in some instances the patients themselves would like to read it own their own.

Interviewer: Apart from the women being in a lot of pain, what other challenges do you face when communicating with these women who undergo emergency caesarean section?

Participant: So sometimes you find that there are those that are unable to read or write ok so in those that are unable to read or write we tend to read for them then they will put a finger print in terms of signing the consent. Sometimes you get patients that can't speak the local languages here or English because sometimes we have patients who come from other countries so it becomes a challenge for them to consent what has to be done so probably we need to find an interpreter or where you can't find an interpreter in terms of consent there is usually someone who can override either a consultant can consent on their behalf at that time, the other issue we usually face is those that are underage they are not able to consent so if there is a relative around they will consent on their behalf and them they will provide assent or if there is nothing it is an emergency caesarean that needs to be done they don't have relatives around so either the consultant can also provide consent in such instances so those are some of the challenges we face, you find they are underage and cant consent or someone doesn't speak the common languages that we use in Zambia. So we need to find an interpreter and that can delay to obtain consent before the operation.

Interviewer: You mentioned that dealing with an emergency time is limited, when do you think is the best time to communicate to the women?

Participant: So in an emergency unfortunately when you make a diagnosis that is when you need to communicate but then after the operation because they are calm you provide information on the operation, findings you can also retaliate on the indication of the operation,

intra operatively this is what we found during the operation. So you find that women are mostly likely to recall what was said after the operation than what you have discussed before the operation before the operation one thing they may pick is why the operation was done, the other things they may not even remember them. But after the operation you have more time to explain to them what really happened and why you did the operation. I feel after the operation in an emergency setting it is different from an elective operation because you have more time but in an emergency setting you need to give more information after the operation.

Interviewer: As we conclude the interview is there anything of importance that you would like to talk about regarding communication between health care providers and women who undergo emergency caesarean section?

Participant: I think probably we just need to mention during the antenatal period that every woman going into labour is at risk of having caesarean section ok people can know the basic indication of emergency caesarean section even before they go into labour during the antenatal period so that this will help them so that should they have one of the indications they will recall that we had discussed this, the way we discuss on the danger signs of pregnancy it is something that we need to bring on board during antenatal to tell people that are pregnant that whoever goes to labour is at risk of emergency caesarean section and these are the common indications of caesarean section and also probably the complications of emergency caesarean section, they can understand because many of them only have myths from the community, that is why we have had instances where we need to do this emergency caesarean section and someone refuses because they want to for example prayed for because of religious affiliations, they will call their leaders who will say no let them not operate on you this is work of the devil and therefore people refuse and others have ended up with poor outcomes. But if they understand antenatally they will have more time to ask questions or research on their own from their relatives or people in society to make that decision when they in labour ward they will understand better.

Interviewer: Doctor, thank you so much for your time.

Participant: You welcome.

PARTICIPANT NUMBER FIVE (RR5)

Interviewer: Welcome to the interview.

Participant: thank you very much.

Interviewer: what is your role when dealing with women who require emergency caesarean section?

Participant: so my role as a health worker is to order that emergency caesarean section depending on the features that the patient may have whether the mother is distressed or the baby is distressed and for us to save either the mother or baby an emergency caesarean section is required. So these are mothers that we talk to we tell them this is the condition that you are having right now and for us to save either you and the baby we need to do an emergency caesarean section. So we tell them that and then we explain what is involved when they go for that emergency caesarean section. The main concern is issues to do with pain, they usually ask are well going to feel pain somehow anxiety or just fear of having an operation so you have to calm them down, no it is a safe procedure we do everything to ensure that it is a safe procedure and painless but is the quickest way we can help you and your baby. We tell them what they are supposed to receive, for example they are supposed to receive medication before the operation and also that during the operation they will be awake so they can communicate how they are feeling, we tell them the duration most likely it will be less than thirty minutes and then they can even see the baby afterwards and we provide them with medication so that during the operation they don't feel pain and after the operation and we keep them in the hospital for about three weeks covering them on anti-biotics and also on pain killers until they are discharged.

Interviewer: describe the process of consenting to emergency caesarean section

Participant: so for a consent to be obtained in an emergency caesarean section, it is usually done by the nurses we order the caesarean section as clinicians and explain to the mother this is what needs to be done and the reason we are doing the caesarean section is for this and this we give the reason why we are doing the caesarean section and we ask them if they are agree or not. Most of the time we don't even ask them whether they agree or not we just order you are going for emergency caesarean section because of this and that so then the nurse comes in to consent them. She explains and asks them if they have any questions and whether they want to go ahead with the operation or not so when they agree the nurses will be the ones to get the

consent, the patient signs but if they are not in a state to sign underage we get the next of kin, the next of kin can sign if we don't have next of kin nearby then our consultant can sign.

Interviewer: what about those that are of consenting age and are fully conscious?

Participant: they can sign.

Interviewer: what challenges do you face when communicating with these women who require emergency caesarean section?

Participant: So sometimes they may not really understand what the caesarean section is done for because after they have done that caesarean section when you see them for review and you ask them why the caesarean section was done they don't know. So maybe it is because of the pain sometimes they may just want the pain to go away so them they will think they had caesarean section because of so much pain. Most of them don't really comprehend the reason why we are doing caesarean section.

Interviewer: Why don't they comprehend?

Participant: ah maybe because they don't really focus on what you are saying especially if they are in labour and it is advanced, they may not focus on what you are saying, they may focus on pain and once you tell them you are going to have caesarean section they will jump to it no matter what three quarters of the women they just want the pain to go away but that is not the reason we do the caesarean section. Now when you see them during review and see why they didn't understand the operation then we explain to them why they had the operation.

Interviewer: you mentioned that the midwives are the ones that obtain written consent, is that the standard?

Participant: it is not the standard ideally we are supposed to consent them because we are the ones that order the caesarean section but we do mostly is just explaining to them and asking if they agree or not and if they agree the nurses will come and do the rest probably because of the numbers and most of the time you are required to see more than one emergency at a time so you may not have time to sit with one woman explain the procedure and consent them and go to the next we may not have that time. You find yourself running from one woman to the other before you are even done you go to the next woman.

Interviewer: Since it is an emergency how much time is required to communicate to the woman?

Participant: it is supposed to be less than thirty minutes, most of the time maybe it will be ten minutes you spend less than ten minutes talking to that woman if she understands you ask whether she has understood or not why she is going to theatre but if you think she understands why she is going to theatre then you are good to go. So less than thirty minutes on average but for an emergency less than ten the communication.

Interviewer: Describe the communication between health care providers and women after they come back from theatre?

Participant: After they come back from theatre the communication now is more detailed because they are relaxed, they are not in pain, so they are able to focus on you completely so we ask her how the baby is doing how they are doing after the operation tell us there complaints majority of the women will complain of pain and headache because the majority of the women we do spinal anaesthesia so one of the side effects of that is headache. They will talk to you about the headache, the paining wound and then you ask them how the baby is doing, why they didn't deliver normally vaginal delivery. So you will be able to gauge whether they understood why they went to theatre or not. If you find that they did not understand then you check through the notes because in theatre they usually indicate why this woman went to have an emergency caesarean section. So therefore you will be able to explain to them why they went to theatre and the outcome how the baby is doing and the findings in theatre what was found, was the uterus normal, was the baby ok, did they bleed too much, so all those things are explained. So the difference is that before the operation it is kind of hurried communication but after the operation there is ample time to discuss with the woman.

Interviewer: As we conclude the interview is there anything of importance that you would like to talk about regarding communication between medical personnel and women who undergo emergency caesarean section?

Participant: I think mostly it will be the fact that these women need to understand that an emergency caesarean section is not a normal way of delivery so therefore it is ordered when there is an urgent need for a caesarean section because most of them will just be screaming

telling you to take them for a caesarean section because they are feeling pain although we do give pain killers in labour but it depends on how far they have gone in labour if they are really advanced we don't give them any pain killers because most of the drugs have an effect of reducing breathing capacity for the baby so therefore we don't give it when someone is nearing delivery. So they need to understand that an emergency caesarean section although it helps the baby and the mother it is very … compared to an elective caesarean section. So an emergency caesarean section has complications because most of the time this is labour that would have progressed maybe the head of the baby would have moved way down into the birth canal the risk to the bladder of the woman, risk to cervix of the woman, the birth canal a lot of injuries can happen during an emergency caesarean section because it is not a normal thing that we normally do so they need to understand that there are risks associated with that.

Interviewer: I would like to sincerely thank you for your time.

Participant: You are welcome.
