# Supplementary material for: Women’s experiences of communication with medical staff before and after emergency caesarean birth in Zambia: A qualitative study
Source: PLoS One. 2026 Apr 9;21(4):e0346694. doi: 10.1371/journal.pone.0346694 (PMC13065054; doi:10.1371/journal.pone.0346694)
Supplement: S8 File — (PDF) [file pone.0346694.s008.pdf]

Code	Definition	Direct quote
Limited time	Insufficient time/time constraints	*"The woman who is undergoing an emergency caesarean section might be not in the right state of mind to be fully explained to, she is bleeding, she is restless so what she needs from you is immediate intervention so those are the constraints which stops you from explaining in full because the woman will be bleeding and she is restless and you need to act and if you look at her eyes she is pleading, save my life."*<br><br>*"So the difference is that before the operation it is kind of hurried communication but after the operation there is ample time to discuss with the woman."*<br><br>*"Since it is an emergency we don't have much time to sit and liaise with the patient so we will be preparing them for theatre while giving psychological care reassuring the woman what will happen and the outcome."*<br><br>*"If it is an emergency it is a very short time, five ten minutes as you are counselling the patient someone is preparing the patient. Then sometimes ok we are talking about consent so it is relative. Sometimes there is no time you need to save this patient you start doing what you have to do."*<br><br>*"It is not like you are going to spend a lot of time explaining why someone is bleeding so you expect the*

		woman to understand her condition because she is seeing the blood and you just say mum you are bleeding, your life is at stake your baby is at stake or someone maybe has distressed baby, so you say we are trying to save your baby's life. So in short the counselling is quite specific and relevant, very specific and relevant."<br><br>*"Just as the word says it is an emergency, so at the moment you have realised here is the problem, that time that communication needs to be done because you don't have time to wait so the moment you make a diagnosis you say ok this is the situation I think we cannot continue waiting you communicate. I feel at that time is the right time though sometimes it may just come suddenly to a woman."*<br><br>*"The process of consenting to ECS, anyway sometimes it depends with the condition, I will give an example if it is a raptured uterus you don't really need to take your time explaining to the patient or relatives, you have to save the life of the mother."*<br><br>*"It should be done within thirty minutes or sixty minutes the delivery should have occurred unless the circumstances are extenuating where for example you have so many similar emergencies yet you, you have immediate operating space then you find that others go up to two hours ok."*

		"It is just a few minutes normally within five minutes you would have actually communicated, explained and she should be able to understand during that time. Most of them will understand, a few may not understand or they may need more time to think about it or they may need to consult usually when they want to consult it is either their husband or their mother which we do allow them to do that especially when they have a phone with them it easy for them to call."
Communication barriers	Anything that hinders effective communication	*"We have no challenges with somebody who reads on their own, they even tell you doctor I have read about eclampsia just remove the baby. So with the educated we have no problems most times. But we have challenges with women who have low levels of literacy. There are times we talk to the partner who maybe more enlightened to convince the woman. But the final decision is in the hands of the woman as long as she is of consent age."*<br><br>*"Some are so illiterate such that when you explain even traditionally what they know is that when you do this you will die even when you explain to them because when they were growing they were told that if you deliver through caesarean you will die those who deliver at the hospital die."*<br><br>*"Sometimes it is very difficult to simplify it to where a layman can*

		understand and you find that we usually tend to give half-truths so that the patient can just try to understand what you are trying to say.”<br><br>*“Sometimes it could be language barrier also when we are communicating sometimes you can be carried away thinking they are getting what you are communicating meanwhile they haven’t gotten anything because this is a referral hospital we don’t expect every woman to understand Nyanja, Bemba so there are some people where you are communicating to them in Nyanja they will just be looking at you, this woman is Lozi, she can’t pick anything.”*<br><br>*“For me the challenges is the language barrier I don’t know Nyanja that much. Sometimes we see patients from Choma (Southern Province) maybe they can be speaking in Tonga I don’t know the language but others they get Bemba others they get Nyanja others don’t but we always work with someone who can interpret to the patient.”*<br><br>*“The challenges, it is mostly language barrier by trying to explain certain things to a woman but they won’t understand, maybe certain terms that you get to use to explain these procedures they don’t understand actually these are challenges we get.”*<br><br>*“When they come back from theatre they are so much in*

		pain even if you will tell them things they are supposed to do it will be very difficult for them to comprehend because they are in pain all they want is that pain to be relieved."<br><br>*"Some of the challenges are making them really understand yeah so I think some could have been in* ***pain*** *so whatever you want to do she says do whatever you need to do to help me and because of* ***pain*** *they want you to go ahead with whatever."*<br><br>*"It is ignorance, there is a lot of illiteracy yeah around and unfortunately that is the majority. It is very difficult to communicate with an illiterate person because conventionally they are illiterate but customarily and traditionally they think they have a lot of information that may not conform the conventional information. So sometimes you have difficulties to get the understanding of this person."*<br><br>*"We really face challenges. They will just say papa (clergy) is praying for us and they are on the phone they will say let me get the guidance from papa. But basically speaking it is supposed to be an emergency operation."*<br><br>*"So it is ignorance, illiteracy very bad. The other categories is the google literacy, literate but through doctor google this person thinks that they can have better information than you because they have been googling."*

Indication for emergency caesarean	Reason for emergency caesarean section	*"We tell them the indication for emergency caesarean section, that one is very important you will be driven to theatre, she has to understand and her risk if she doesn't take that operation."*<br><br>*"We explain the condition that she really need caesarean section and we explain the advantages and if we are not going to do caesarean section what will happen (consequences)."*<br><br>*"So the most important is explaining why we are taking this woman to theatre and the consequences of not going there. Those are very critical."*<br><br>*"The most important things are to make them understand why they cannot deliver vaginally and why it needs to be done as an emergency that is very key."*<br><br>*"Sometimes they may not really understand what the caesarean section is done for because after they have done that caesarean section when you see them for review and you ask them why the caesarean section was done they don't know. So maybe it is because of the pain sometimes they may just want the pain to go away so them they will think they had caesarean section because of so much pain. Most of them don't really comprehend the reason why we are doing caesarean section."*

Work overload/low staffing levels	Large number of patients/tasks & staff shortages	*"You find yourself running from one woman to the other before you are even done you go to the next woman."*<br><br>*"What is lacking on the medical side is that sometimes we are understaffed, I have five patients to take to theatre. I might not take all that time to finish explaining to them but where I needed to do so I may not manage depending on how the situation is because we are understaffed. You find that there is only one nurse in the whole ward you take care of many patients maybe five of them are going to theatre it becomes a challenge for me to give the total information to a patient."*<br><br>*"We need to reinforce on the practice take our time, sometimes we rush maybe there is another patient next door who is bleeding maybe there is emergency which is coming you don't take enough time maybe three to five minutes explaining to her it is an important step that needs to be ah reinforced from our part."*<br><br>*"Sometimes the communication why it is not like fully given and the way it is supposed to be given maybe it is because of having being short staffed there are people whereby you have other things to attend to so you don't find all that time to dedicate it to this woman and give her the full informed eh message. The shortage of staff makes us not to give proper communication to the woman."*

		"There some whereby they are so stressed some people (medical staff) don't know how to handle their stress so sometimes they will not even communicate anything so the woman will just be asking and the more the woman is asking the other cadre will feel like they are being troubled or this woman is just troubling them but it is because the information hasn't been given in totality to her so the women will continue asking."<br><br>*"You may have one doctor doing a round in labour ward. there may not be good numbers of doctors to allow you to spend all this time on one patient while others are screaming in the same room because you are going to start explaining, explaining the other patient needs my attention, so your mind gets divided."*<br><br>*"There are some challenges you find maybe the doctor there are also few in terms of numbers Registrars you find people are depleted, you find one doctor is attending to more than expected patients."*
Risk communication	Explanation of surgical risks and complications	*"I think you and I have boarded a plane, if the airline starts concentrating on the risks of air travel someone will turn back at the door of the plane. So you give mostly about the positives of emergency caesarean section. Of course risks of the surgery can be given but it shouldn't outweigh the positives and then you assure the patient*

		that these are trained people, the whole team anaesthetist, the surgeon, theatre nurse, and the midwife so we are trained to have no room for error that is why we don't emphasise on the risks of surgery. So we give mostly the positives."<br><br>*"I need to admit that the informed consent sometimes is not properly processed we need to sit with the pregnant woman and explain in detail but sometimes there are some gaps in terms of explaining the possible complications for example that can arise."*<br><br>*"But most of the times the communication is good especially when you are in the low cost wards, these women they are very easy to engage in a discussion and they are very obedient usually we don't have so many challenges with them."*<br><br>*"The communication basically has to involve explaining the need for the operation, you explain to them that in case you delay (in consenting) the condition will worsen and the baby may die."*
Limited understanding of medical condition	Patient unaware of seriousness of condition	*"I think the challenges we face when communicating with the mothers are those who don't understand their conditions. I think one condition we usually have problems with is eclampsia or severe pre-eclampsia, some women do not understand how severe this condition is. However, where there is no problem like bleeding she is seeing the*

		blood and she knows the consequences of not consenting to a caesarean section if she is bleeding."<br><br>*"Another challenge I would say is the lack of knowledge on the conditions because there are some women whereby even if it is an emergency you are communicating to them they are not taking it serious, they will not even like respond and do the things the way they are supposed to be done and for some just that attitude of ah they just want to do an experiment on me so some of those challenges are the ones that we meet, the lack of knowledge and just the attitude of the patients,"*
