# Supplementary material for: Women’s experiences of communication with medical staff before and after emergency caesarean birth in Zambia: A qualitative study
Source: PLoS One. 2026 Apr 9;21(4):e0346694. doi: 10.1371/journal.pone.0346694 (PMC13065054; doi:10.1371/journal.pone.0346694)
Supplement: Attachment — Submitted filename: PONE-D-25-47624Pj.pdf [file pone.0346694.s009.pdf]

PLOS ONE

Women’s experiences of communication with medical staff before and after emergency caesarean delivery in Zambia: a qualitative study.

--Manuscript Draft--

**Manuscript Number:**	PONE-D-25-47624
**Article Type:**	Research Article
**Full Title:**	Women’s experiences of communication with medical staff before and after emergency caesarean delivery in Zambia: a qualitative study.
**Short Title:**	Women's emergency caesarean birth communication experiences
**Corresponding Author:**	Effie Moreblessing Mphande, MPH<br>University of Zambia<br>Lusaka, ZAMBIA
**Keywords:**	Communication; experiences; emergency caesarean birth
**Abstract:**	Background<br><br>Communication between patients and staff during emergency caesarean section is important for ensuring positive outcomes and reducing the negative psychological impact of the procedure. This study aimed to explore patient-healthcare provider communication before and after emergency caesarean birth at the University Teaching Hospitals, Women and Newborn Hospital in Lusaka, Zambia.<br><br>Methods<br><br>Qualitative interviews were conducted with 30 women who were purposively sampled from the hospital’s wards. Thematic analysis was used for data analysis.<br><br>Results<br><br>Communication between healthcare providers and women who underwent emergency caesarean delivery was inadequate, untimely and lacked detail about the surgical procedure. As a result, women felt afraid, angry, anxious and lacking emotional support, resulting in a sense of worthlessness and helplessness. The use of medical jargon by healthcare providers, misconceptions about caesarean section, the presence of pain and poor staff attitudes towards mothers were identified as some of the communication barriers.<br><br>Conclusion<br><br>Findings suggested the need for medical staff to use simple language for patients. Interventions are needed to address the current gaps in communication between patients and staff at the University Teaching Hospitals Women and Newborn Hospital. Clear communication can help to improve the overall experience of caesarean birth.
**Order of Authors:**	Effie Moreblessing Mphande, MPH
	Doreen Chilolo Sitali
	Jakub Gajewski
**Additional Information:**	
**Question**	**Response**
**Financial Disclosure**<br><br>Enter a financial disclosure statement that describes the sources of funding for the work included in this submission. Review the submission guidelines for detailed	The author(s) received no specific funding for this work.

Powered by Editorial Manager® and ProduXion Manager® from Aries Systems Corporation

requirements. View published research articles from *PLOS ONE* for specific examples.<br><br>This statement is required for submission and **will appear in the published article** if the submission is accepted. Please make sure it is accurate.<br><br>**Funded studies**<br>Enter a statement with the following details:<br>• Initials of the authors who received each award<br>• Grant numbers awarded to each author<br>• The full name of each funder<br>• URL of each funder website<br>• Did the sponsors or funders play any role in the study design, data collection and analysis, decision to publish, or preparation of the manuscript?<br><br>Did you receive funding for this work?	
**Competing Interests**<br><br>Use the instructions below to enter a competing interest statement for this submission. On behalf of all authors, disclose any competing interests that could be perceived to bias this work—acknowledging all financial support and any other relevant financial or non-financial competing interests.<br><br>This statement is required for submission and **will appear in the published article** if the submission is accepted. Please make sure it is accurate and that any funding sources listed in your Funding Information later in the submission form are also declared in your Financial Disclosure statement.<br><br>View published research articles from *PLOS ONE* for specific examples.	The authors have declared that no competing interests exist.

**NO authors have competing interests**<br><br>Enter: *The authors have declared that no competing interests exist.*<br><br>**Authors with competing interests**<br><br>Enter competing interest details beginning with this statement:<br><br>*I have read the journal's policy and the authors of this manuscript have the following competing interests: [insert competing interests here]*<br><br>* typeset	
**Ethics Statement**<br><br>Enter an ethics statement for this submission. This statement is required if the study involved:<br><br>• Human participants<br>• Human specimens or tissue<br>• Vertebrate animals or cephalopods<br>• Vertebrate embryos or tissues<br>• Field research<br><br>Write "N/A" if the submission does not require an ethics statement.<br><br>General guidance is provided below. Consult the submission guidelines for detailed instructions. **Make sure that all information entered here is included in the Methods section of the manuscript.**	The study involved human participants. The study complied with ethical standards outlined in the Belmont Report and Declaration of Helsinki. Ethical approval was obtained from the University of Zambia Biomedical Research Ethics Committee approval number 1445-2020 and National Health Research Authority (Ref No: NHRA00011/4/03/2021). Permission was also sought from the Lusaka Provincial Health Office (PHOLSK/101/8/11) and the University Teaching Hospitals Women and Newborn Hospital. Written informed consent was obtained from all participants before they took part in this study. Before obtaining consent, the purpose, procedure, duration, type of research intervention, risks and benefits of the study were explained to participants in a simple and clear language. They were also informed that participating in the study was voluntary and that they had the right to refuse. Participants were also told that they could withdraw from the study if they wished to do so and that their withdrawal would not affect access to healthcare at the Women and Newborn Hospital. They were also assured of confidentiality. Prior to being interviewed, participants were informed that they would receive a summary of results before they are shared at a conference and published in a journal. Participants were also given an opportunity to ask questions. Prior to being interviewed, participants were given an information sheet and told not to decide there and then. The next day if they agreed to participate in the study, they gave written consent in the presence of their witnesses who also appended their signatures. After each interview, participants were asked to confirm if the researcher's summary accurately reflected their views. They were also provided with the researchers' contact details in an event that they had questions or needed further clarification.

**Format for specific study types**<br><br>**Human Subject Research (involving human participants and/or tissue)**<br>• Give the name of the institutional review board or ethics committee that approved the study<br>• Include the approval number and/or a statement indicating approval of this research<br>• Indicate the form of consent obtained (written/oral) or the reason that consent was not obtained (e.g. the data were analyzed anonymously)<br><br>**Animal Research (involving vertebrate animals, embryos or tissues)**<br>• Provide the name of the Institutional Animal Care and Use Committee (IACUC) or other relevant ethics board that reviewed the study protocol, and indicate whether they approved this research or granted a formal waiver of ethical approval<br>• Include an approval number if one was obtained<br>• If the study involved *non-human primates*, add *additional details* about animal welfare and steps taken to ameliorate suffering<br>• If anesthesia, euthanasia, or any kind of animal sacrifice is part of the study, include briefly which substances and/or methods were applied<br><br>**Field Research**<br><br>Include the following details if this study involves the collection of plant, animal, or other materials from a natural setting:<br>• Field permit number<br>• Name of the institution or relevant body that granted permission	
**Data Availability**<br><br>Authors are required to make all data underlying the findings described fully available, without restriction, and from the time of publication. PLOS allows rare exceptions to address legal and ethical concerns. See the PLOS Data Policy and FAQ for detailed information.	No - some restrictions will apply

A Data Availability Statement describing where the data can be found is required at submission. Your answers to this question constitute the Data Availability Statement and **will be published in the article**, if accepted.<br><br>**Important:** Stating 'data available on request from the author' is not sufficient. If your data are only available upon request, select 'No' for the first question and explain your exceptional situation in the text box.<br><br>Do the authors confirm that all data underlying the findings described in their manuscript are fully available without restriction?	
**Describe where the data may be found in full sentences. If you are copying our sample text, replace any instances of XXX with the appropriate details.**<br><br>• If the data are **held or will be held in a public repository**, include URLs, accession numbers or DOIs. If this information will only be available after acceptance, indicate this by ticking the box below. For example: *All XXX files are available from the XXX database (accession number(s) XXX, XXX.*).<br>• If the data are all contained **within the manuscript and/or Supporting Information files**, enter the following: *All relevant data are within the manuscript and its Supporting Information files.*<br>• If neither of these applies but you are able to provide **details of access elsewhere**, with or without limitations, please do so. For example:<br><br>*Data cannot be shared publicly because of [XXX]. Data are available from the XXX Institutional Data Access / Ethics Committee (contact via XXX) for researchers who meet the criteria for access to confidential data.*<br><br>*The data underlying the results presented in the study are available from (include the name of the third party*	Due to the confidentiality of participants, full transcripts are not publicly available. However, anonymized data could be shared upon request to the corresponding author.

and contact information or URL).<br>• This text is appropriate if the data are owned by a third party and authors do not have permission to share the data.<br><br>* typeset	
Additional data availability information:	Tick here if the URLs/accession numbers/DOIs will be available only after acceptance of the manuscript for publication so that we can ensure their inclusion before publication.

Manuscript
Click here to access/download;Manuscript;FINAL VERSION EMCS COMM EXPERIENCES_submission_authors.docx

Women's experiences of communication with medical staff before and after emergency caesarean delivery in Zambia: a qualitative study.

Authors: Effie Moreblessing Mphande [1, 3*], Doreen Chilolo Sitali [1], Jakub Gajewski [2]

1. [#a] Department of Health Promotion and Education, School of Public Health, University of Zambia, Lusaka, Zambia.
2. [#a] Institute of Global Surgery, School of Population Health, Royal College of Surgeons in Ireland.
3. [#b] Department of Social Work and Community Development, University of Johannesburg, South Africa.

*Corresponding author

E-mail: effiemphande@gmail.com, emphande@uj.ac.za (EMM)

Abstract

Background

Communication between patients and staff during emergency caesarean birth is important for ensuring positive outcomes and reducing the negative psychological impact of the procedure. This study aimed to explore patient-healthcare provider communication before and after emergency caesarean birth at the University Teaching Hospitals, Women and Newborn Hospital in Lusaka, Zambia.

Methods

Qualitative interviews were conducted with 30 women who were purposively sampled from the hospital's wards. Thematic analysis was used for data analysis.

Results

Communication between healthcare providers and women who underwent emergency caesarean delivery was inadequate, untimely and lacked detail about the surgical procedure. As a result, women felt afraid, angry, anxious and lacking emotional support, resulting in a sense of worthlessness and helplessness. The use of medical jargon by healthcare providers, misconceptions about caesarean birth, the presence of pain and poor staff attitudes towards mothers were identified as some of the communication barriers.

Conclusion

Findings suggested the need for medical staff to use simple language for patients. Interventions are needed to address the current gaps in communication between patients and staff at the University Teaching Hospitals Women and Newborn Hospital. Clear communication can help to improve the overall experience of caesarean birth.

Keywords: *communication, experiences, emergency caesarean birth.*

Introduction

Caesarean birth remains the most appropriate option for several obstetric problems [1] and contributes greatly to safe motherhood. As a result, communication between women undergoing emergency caesarean delivery and medical staff (Nurses/Midwives and obstetricians) is crucial for ensuring positive outcomes and reducing the negative psychological impact of the procedure [2]. Effective communication can help to alleviate women's fears, reduce anxiety and improve the overall experience of caesarean birth [3–6]. Clear, timely and empathetic communication enables women to understand the reasons for the procedure, the risks and benefits, and the steps taken during the procedure [7]. This can lead to better patient compliance, satisfaction and trust in the medical staff [7]. Furthermore, good communication can help women feel more in control and empowered, which can help them cope better with the procedure [6]. Poor communication can lead to negative experiences, dissatisfaction with care and mistrust towards the medical staff [6–9].

This study aimed to explore the experiences of women concerning communication with medical personnel before and after an emergency caesarean birth at the University Teaching Hospital in Lusaka, Zambia. This research aims to contribute to the understanding of the current communication practices and gaps in maternal health identifying gaps and informing strategies to improve the patient experience for women who undergo emergency abdominal delivery in similar settings.

Materials and methods

Study design and sampling

We employed a phenomenological study design to understand women's experiences of communication with medical personnel before and after emergency caesarean birth at the University Teaching Hospitals Women and Newborn Hospital (UTHWNH). Purposive sampling was used to select 30 women from the hospital's five wards.

Participant recruitment

The participants were approached individually after formal introductions to the study done in the wards. Participants included women who had undergone emergency caesarean delivery. Women who were critically ill after the emergency caesarean delivery and those who had stillbirths were excluded. Information regarding demographical characteristics was obtained from the patients' files.

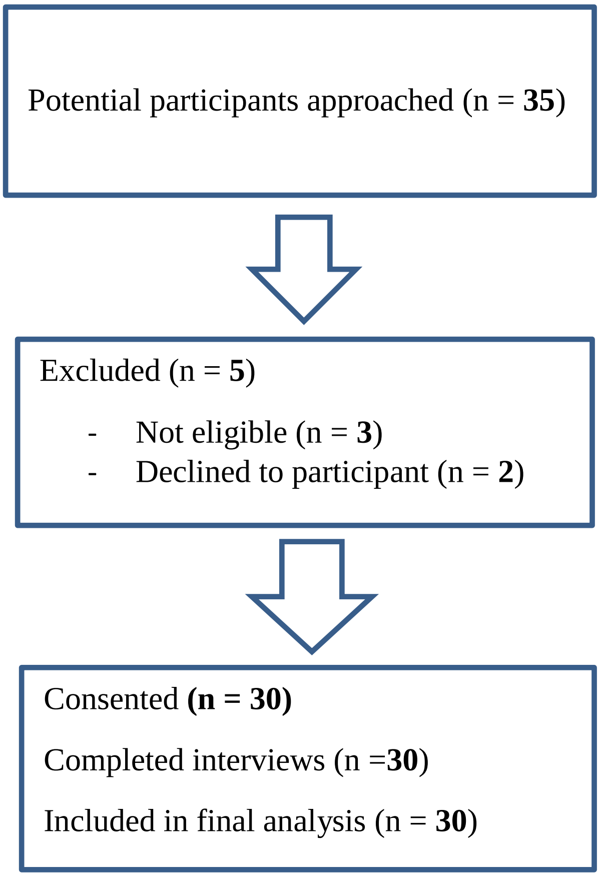

Fig 1. Flowchart of participant recruitment

Data collection

An interview guide with open-ended questions was used to explore women's experiences of communication with healthcare providers before and after emergency caesarean birth. Face-to-face, in-depth interviews were conducted between 17 March and 11 April 2021, within 48 hours post-operatively. To ensure participant privacy, interviews took place in treatment rooms and private rooms of both standard (non-fee-paying) and premium wards.

The interview guide was professionally translated into the two main languages spoken in the capital city by a translator from the Ministry of Education. Interviews were conducted in either English or local languages, depending on participant preference, and each session lasted approximately 30 to 45 minutes. Narratives were audio-recorded using a digital voice recorder.

Data collection ceased once no new information emerged, indicating thematic saturation. Participants were asked to describe their experiences of communication with healthcare providers both before and after undergoing emergency caesarean delivery.

To ensure trustworthiness, data was also cross-checked with the participants after the interviews to verify their responses. We followed the COREQ checklist for reporting qualitative data in this study [10].

Ethical approval and consent to participate

Ethical approval was obtained from the University of Zambia Biomedical Research Ethics Committee (REF. No. 1445-2020) and National Health Research Authority (Ref No: NHRA00011/4/03/2021). Permission was also sought from the Lusaka Provincial Health Office (PHOLSK/101/8/11) and the University Teaching Hospitals Women and Newborn Hospital. Written informed consent was obtained from all participants before they took part in this study. As part of the informed consent process, the purpose of the study was explained to participants. Before interviews, they were given an opportunity to ask questions and sign the consent form. Participants were informed that their participation was voluntary and that they could withdraw from the study at any point if they wished to. They were assured that their withdrawal from the study would not affect access to health services.

Data analysis

The recordings were transcribed verbatim and field notes were typed and saved as a Word document. Interviews, which were done in local languages were transcribed and translated into English by personnel from the Zambia National Broadcasting Corporation (ZNBC) home language section. Data collection and analysis occurred simultaneously. Data were analysed using a thematic analysis approach [11]. An inductive iterative process of reading and rereading

the transcripts produced subcategories for data analysis within research areas of interest. This was followed by developing preliminary codes. 55 initial codes were developed. Similar codes were grouped and similar patterns were put together resulting in 19 final codes. After that, themes were created and a coding tree was developed. NVIVO was used to facilitate the analysis. Direct quotes were used to illustrate specific concepts.

Research team and reflexivity

The interviews were conducted by EMM (Zambian female Journalist) who at the time of the study had a postgraduate qualification in Peace, Leadership and Conflict Resolution. Before the study, EMM had completed coursework for the Master's degree in Public Health, Health Promotion and Education. The selection of the research topic was influenced by the researcher's communication professional background. The journalism background helped balance empathy and objectivity as well as upholding ethical standards. While conducting the study, EMM reflected on her positionality, supported by conversations with the other researchers. This was important to minimise bias.

The use of in-depth interviews enabled to probe further and ask follow-up questions. Through this process, the interviewer gained a deeper perspective and understanding of the participants' point of view. Before the interviews, none of the participants were known to EMM, either in a personal or professional capacity. To enhance credibility, the study was supported by JG and DCS, both with vast experience and expertise in qualitative research in Zambia. The transcripts and audio recordings were shared with them for their analytical input and to agree on codes and themes between the research team.

Results

Participants' attributes:

A total of 30 participants completed the study. Table 1 summaries their demographic characteristics. The majority of participants were aged between 30 and 39, married and school leavers. 19 of them had an emergency caesarean birth for the first time while eight had the operation for the second time and three were being operated on for the third time.

Table 1. Participants' attributes (n = 30).

Mothers	Age	No. of Emergency C-section	First time mother	Level of education
1.	38	2	No	degree
2.	31	1	Yes	Master's degree
3.	38	1	Yes	Degree
4.	35	3	No	No formal education
5.	25	1	Yes	Diploma
6.	26	2	No	No formal education
7.	32	1	No	Secondary education
8.	35	1	No	Primary education
9.	32	2	No	Primary education
10.	40	1	Yes	Diploma
11.	29	2	No	Secondary education
12.	38	2	No	Secondary education
13.	35	3	No	No formal education
14.	34	2	No	Secondary education
15.	43	1	No	Degree
16.	38	1	No	Diploma
17.	33	3	No	Primary education
18.	33	1	No	Primary education
19.	32	2	No	No formal education
20.	26	1	Yes	Diploma
21.	30	2	No	Primary education
22.	33	1	No	No formal education
23.	29	1	Yes	Certificate
24.	36	1	No	Diploma
25.	26	1	Yes	Degree
26.	28	1	No	No formal education
27.	26	1	No	Primary education
28.	19	1	Yes	Secondary education
29.	35	1	Yes	Diploma

30.	30	1	No	Primary education

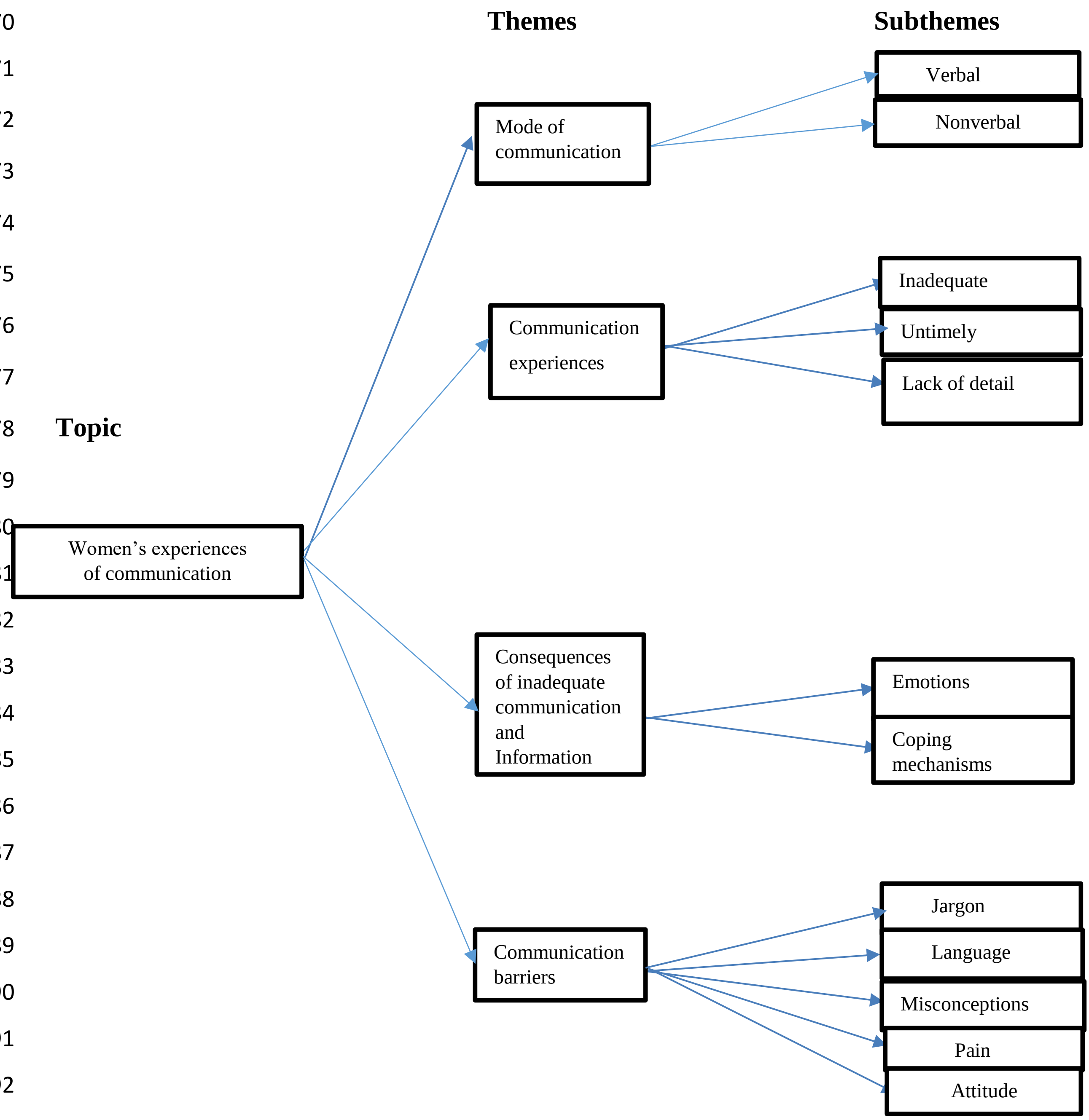

Fig 2. Coding tree

Mode of communication

The mode of communication between healthcare providers and women who underwent emergency caesarean delivery was verbal, nonverbal and written. Verbal communication involved spoken words, while nonverbal communication was through facial expressions, tone of voice, voice volume, voice pace, eye contact, silence, distance (physical space), body movements and posture, hand gestures and touch. Verbal and nonverbal communication was face-to-face whenever medical personnel and women communicated. Written communication was in the form of obtaining written consent from the women before they could be operated on. In most cases, the consent form was signed in the absence of the medical doctor. Communication between medical personnel and women who underwent emergency caesarean birth was usually firstly verbal and thereafter written consent was obtained by midwives. In the words of one participant:

"The doctor told me I was going to have caesarean section then the nurses who were preparing me for theatre are the ones who gave me the consent form," (Mother 15).

The women provided written consent by appending their signatures or putting a thumbprint on the form. The consent form was in English. Communication between healthcare providers and women took place in the admission rooms of the labour ward, high dependency unit or obstetrics intensive care unit before they were taken to the theatre. According to study participants, the purpose of the consenting process was to explain the indication for emergency caesarean birth, the risks of not performing the operation and obtaining verbal and written consent. Communication was often restricted to information provision, convincing the women to accept emergency caesarean delivery and avoid complications.

Emergency caesarean delivery communication experience

The communication between healthcare providers and women who underwent emergency caesarean birth was reported to be inadequate, untimely and lacking detail about the surgical procedure. The majority of the study participants said it was incomplete in the sense that it lacked an explanation of the women and baby's condition, classification of emergency caesarean birth, amount of pain women would experience after the procedure, anaesthesia side effects after the operation, consequences of not adhering to post caesarean birth advice and recovery trajectory. For example, one mother indicated that she had not been advised to lay down for eight hours after the emergency caesarean birth. She reported that she only learnt about this after she questioned medical personnel on the cause of her excruciating headache.

"You can imagine the damage I would have done to myself. Just that piece of information, which they know and could have communicated," (Mother 1).

Most of the study participants said communication also lacked clarity. Regardless of the emergency, women expected medical personnel to communicate clearly before and after the surgical procedure.

Some study participants said that the communication also lacked regular updates. For instance, mothers who had been notified of the need for emergency caesarean delivery but were not taken into theatre immediately desired to be communicated to about the reason for the delay. Some women said they expected healthcare providers to communicate the delay immediately after they noticed that the operation was not going to take place at once. They thought they needed to be taken into theatre immediately after being told that they were required to give birth through emergency caesarean delivery. Not being taken to the theatre instantly left them wondering whether their case was an emergency or not.

Some of the participants wanted to be given a timeframe in which they would be taken to the theatre so that they could determine when to eat. Some women reported starving for over 17 hours before being taken to the operating room.

Furthermore, some of the interviewed mothers reported that often the communication lacked detail about the medical procedure in that it did not contain the steps to be taken during surgery and the duration of the operation. They indicated that it also lacked information about risks associated with the operation.

"You have to make somebody understand what they are getting themselves into and you can mention the odds of a successful emergency caesarean section because I didn't even know anything about the procedure, how much risk do I have, is it possible that it might not be successful, I didn't even have that information you know so if I who is a health professional don't know what more somebody for instance these guys from rural areas who have never even gone to school?" (Mother 2).

However, some women reported that they were told about the need for emergency caesarean birth, the procedures involved and the consequences of not being operated on. One participant pointed out that before emergency caesarean birth she had been informed that she would experience pain for two weeks after the operation.

"They explained to me what I went through before they conducted a caesarean section on me. I was told what I would experience, I was told the pain would be there for about two weeks," (Mother 10).

In several instances, rapport was lacking between healthcare providers and the mothers. Most mothers viewed the uncommunicative attitude of medical personnel as a hindrance to asking questions or seeking clarity. Women from both low and high-cost medical wards felt some of the staff were impolite making it difficult to communicate with them.

Some women reported that they did not want to ask questions for fear of upsetting medical staff. This was common among all the participants regardless of their level of education.

"In my case I was in pain so I didn't want to ask questions for fear of upsetting them because I was the one who was in need," (Mother 12).

Consequences of inadequate communication and information

Due to inadequate communication and information, women developed different types of emotions such as sadness, fear, anger and surprise. This affected effective communication with healthcare providers. Women also expressed the desire to know what to expect after the emergency caesarean section to minimise their fear, anxiety and nervousness. Most of the women reported being anxious after experiencing the side effects of anaesthesia. Women indicated that experiencing side effects such as shivering after an emergency caesarean birth could have been normal but because they were not informed, they thought they were going to have life-changing consequences or die.

"I didn't understand why I was feeling very cold but later on after I asked they told me it was the medication that it would clear afterwards but with me I felt hmm this was not normal am I going to be ok because when I came (to the ward) I told mum to put blankets and close the windows but nothing helped not until after everything. I think if someone had told me what to expect after caesarean section that would have helped," (Mother 16).

"Being an emergency no one prepares for an emergency but maybe the time the nurse was preparing me to go into theatre maybe she should have given me a hint about one or more things that are important so that my mind is ready for it," (mother 15).

As a result of inadequate communication, women reported that they also developed coping mechanisms such as seeking information from the internet, friends, relatives and those who had emergency caesarean birth as postnatal and antenatal mothers were being nursed in the same ward. This was common among first-time mothers and those who had the surgical procedure for the first time regardless of their level of education.

Barriers to effective communication

Women including those with tertiary education indicated that they had difficulties understanding what medical personnel communicated. The majority of women reported that some healthcare professionals did not know how to explain the medical terms they were using in an understandable, lay language manner. They desired medical personnel to explain their condition so that they could relay the information to whoever would be taking care of them. Others wanted a situation where they would be made to understand medical issues so that they could explain to family members and friends the reason they had the surgical procedure.

Language was also identified as a barrier to communication. Some of the participants reported that medical personnel did not use local languages they understood when communicating with them. They also stated that the hospital staff used local languages popular in the capital city, which other patients from more remote places could not understand. This sentiment was raised by mothers in the low-cost ward regardless of their level of education. Mothers wanted medical personnel to use a diversity of language when giving out information to patients.

"For this hospital they use a lot of Bemba and Nyanja and we see a lot of people in here, we are not all Bembas and Nyanjas yes. Maybe if there could be a diversity of language so that medical staff can give information that everybody requires regardless of the language the patients speak," (Mother 28).

The other barrier to communication was misconceptions women had about emergency caesarean delivery. Some perceived emergency caesarean birth as an act of God (uncontrollable natural force), which only God could choose or inflict on mothers.

This view was expressed by those who had staunch Christian faith regardless of their level of education. In addition, emergency caesarean birth was seen as an act of kindness especially among those with low levels of education.

"It is by the grace of God that they chose this path for me, I did not choose it for myself," (Mother 10).

"God has the final say. God guided me on what to follow and accept caesarean section," (Mother 18).

In consequence, some women felt that medical doctors had acted kindly by conducting emergency caesarean delivery to save their lives and that of their babies. Learning about the need to have an emergency caesarean birth alarmed some of the women and provoked thoughts of being in a serious condition. Before being operated upon, all the women knew about caesarean birth, however, the majority did not know that there was an emergency caesarean delivery. The mothers were under the impression that all those who gave birth through caesarean delivery did so on their own accord.

The presence of pain was also identified as a barrier to communication. Mothers who were in advanced labour desired to be taken for a caesarean delivery before being provided with information as the pain was unbearable. They indicated that they were tired and just wanted the pain to go away. This was common among mothers with low levels of education and who had been referred from lower-level hospitals. One shared:

"Hmm.....I was in pain, I didn't know anything when I went in theatre but I just signed the form because I really needed help," (Mother 14).

Staff attitude towards mothers also hampered communication. There was a reported feeling of lack of respect from healthcare providers. The majority of the participants felt that they were spoken to rudely and intimated that it would be impossible to have proper communication when the other party was disrespectful. A sense of mistrust was perpetuated by a lack of respect from healthcare providers in the short time the women were at the hospital. Some women indicated that they were shouted at or yelled at by some nurses instead of being explained to.

"There were some things that I did not understand but I was surprised that I was shouted at. Some of us don't understand medical issues because it is not in our line of work. So it is better they explain instead of shouting. Like in my case, this was my first emergency caesarean section but they shouted at me on things that I didn't even know or understand," (Mother 8).

Other participants expressed similar concerns mentioning explicitly some of the language used:

"I would rather they explain that when you do this and that it will affect you this way than some other nurses come out to say <I am not the one who impregnated you>., Such words are not good. So, I would really love to see a change on that," (Mother 23).

However, not all study participants reported similar concerns, five women intimated they were treated in a dignified manner. As one woman explained:

"They treated me well and spoke to me nicely as a patient is supposed to be handled," (Mother 22).

Discussion

This is the first study in Zambia investigating communication during emergency caesarean delivery in a tertiary hospital. It offers valuable insights to inform strategies aimed at improving the quality of emergency caesarean birth care, with potential applicability across the country and beyond. While rooted in the Zambian context, particularly at the University Teaching Hospitals Women and Newborn Hospital in Lusaka, the findings align with existing literature from other sub-Saharan African countries, suggesting broader relevance across similar healthcare systems. Consistent with findings from a study in Malawi [12], face-to-face communication—both verbal and nonverbal—emerged as a key mode of interaction between women and healthcare providers. Such communication allows patients to interpret nonverbal cues [13] and anticipate providers' responses. In our study, several women reported refraining from asking questions due to perceived nonverbal signals suggesting that doing so might upset healthcare staff. This reflects entrenched power imbalances that discourage open dialogue—a trend similarly reported in Uganda, Tanzania, and Nigeria [14 - 17]. These studies demonstrate how patients rely on nonverbal indicators such as gestures, facial expressions, posture, and attire to gauge whether the clinical environment is welcoming or intimidating [18].
At University Teaching Hospitals Women and Newborn Hospital, women's hesitation to speak up suggests that hierarchical and non-inclusive communication practices may inhibit patient engagement in maternal care. Most participants expressed dissatisfaction with the quality of communication from healthcare providers, often linking negative emotions to poor interactions. Similar to findings from other regional studies [9, 18], many women attributed their distressing birth experiences to a lack of information and ineffective communication. One possible explanation is that healthcare providers may only communicate what they perceive as critical—overlooking the importance of explaining the reasons for surgery, operative risks, recovery expectations, and potential complications.

Women in our study explicitly expressed the need to understand the duration of the procedure, outcomes for their baby, and the consequences of non-adherence to post-operative advice, echoing findings from other studies in sub-Saharan Africa [19, 20]. The absence of clear communication before and after the emergency caesarean delivery contributed to widespread anxiety and mistrust. For example, some women questioned whether their surgery was truly urgent due to unexplained delays. Unaware that other high-risk cases were being triaged, they interpreted the lack of information as negligence. This mirrors findings from a study in the US, where women also reported frustration with inadequate communication throughout obstetric emergencies [21].

Furthermore, the emotional state of patients—marked by fear, anxiety, and a lack of emotional support—likely influenced both the reception and perception of communication. Similar associations between emotional distress and impaired communication have been documented in Spain [22], where patients' apprehension and emotional instability were shown to disrupt effective interactions with healthcare staff.

In response, many women developed coping strategies. They sought information from peers in the maternity ward, as well as online sources, friends, and relatives. This pattern aligns with previous research showing that in the absence of adequate counselling from healthcare providers, women turn to informal networks and digital resources [9, 23]. Other studies suggest that patients increasingly consult the Internet as their first source of health information, often before interacting with medical personnel [23].

Our study identified several barriers to effective communication between providers and women undergoing emergency abdominal delivery. These included the use of medical jargon, language differences, misconceptions about caesarean deliveries, pain, and staff attitudes. Many participants struggled to understand healthcare messages due to complex terminology, a

challenge also reported in other studies across the region. Language barriers further complicate communication, especially in multilingual settings such as Zambia, where several local languages are spoken. Previous studies and research from other countries [24–26] confirm that language discordance remains a persistent obstacle in healthcare delivery. Pain was another significant barrier. In some cases, severe discomfort prevented women from engaging meaningfully with providers or processing the information given. Moreover, healthcare staff were sometimes reluctant to provide explanations during periods of intense pain, deeming such communication ineffective—a view supported by similar findings in Malawi [12]. While this rationale may be understandable, it highlights the need for timing and sensitivity in patient interactions.

Lastly, the attitude of some healthcare providers emerged as a key factor affecting communication. Reports of disrespectful or dismissive behaviour suggest a broader cultural issue within the facility, contributing to breakdowns in provider-patient interaction. Similar issues have been reported in other studies from Zambia and neighbouring countries [7, 27], underscoring the importance of addressing healthcare culture and provider behaviour as part of efforts to improve maternal care.

Strengths and limitations

The strength of this study is that it is the first to explore women's experiences of communication with healthcare providers before and after emergency caesarean birth. This study novel provides insights into maternal care as there is limited research in this area. However, there are several limitations in our study. Firstly, the study only documented the views of patients and did not represent the views of the healthcare providers which could have shed a different light on the issue of communication in the situation of emergency. However, this is not a weakness since the researchers' scope was limited to women, yet further work on

the voice of the providers regarding medical communication is needed to fully understand this phenomenon. Another limitation is that for interviews done in the local languages some medical terms for example *mwadzidzidzi* (emergency) could not be understood by some women. To mitigate this, short descriptive terms (colloquial language) such as “fast fast” were used by the lead researcher. However, there is no guarantee that patients fully understood the meaning. Lastly, it is a single-site study which limits its generalisability, and more research is needed to understand whether similar experiences are common in other tertiary facilities and at other levels of care.

Conclusion

This study highlights the critical role of communication in shaping women’s experiences of emergency caesarean birth, demonstrating how interpersonal dynamics, emotional well-being, and systemic barriers influence patient engagement and satisfaction. The findings resonate with evidence from other sub-Saharan African countries, pointing to a regional challenge that warrants broader investigation. Future research should explore interventions to improve provider-patient communication, particularly in high-stress, multilingual, and resource-limited settings. Comparative studies across diverse healthcare systems in sub-Saharan Africa could further illuminate context-specific and shared barriers, while implementation research is needed to evaluate strategies that promote respectful, inclusive, and patient-centred communication in maternal care.

Acknowledgments

The research team would like to thank the participants who in the most sensitive period in their lives agreed to participate in this study.

Authors' contributions

Effie Moreblessing Mphande: conceptualisation, methodology, data collection, data curation, data analysis and writing original draft of the manuscript.

Jakub Gajewski: conceptualisation, data analysis, critically reviewed and edited the manuscript and supervised the study.

Doreen Chilolo Sitali: conceptualisation, methodology, reviewed the manuscript and supervised the study.

JG and DCS are Joint Senior Authors.

References

1. Gonen R, Tamir A, Degani S. Obstetricians' opinions regarding patient choice in cesarean delivery. Obstetrics & Gynecology. 2002 Apr 1;99(4):577-80.
2. Butow P, Hoque E. Using artificial intelligence to analyse and teach communication in healthcare. The breast. 2020 Apr 1;50:49-55.
3. Van der Pijl MS, Hollander MH, van der Linden T, Verweij R, Holten L, Kingma E, et al. Left powerless: A qualitative social media content analysis of the Dutch# breakthesilence campaign on negative and traumatic experiences of labour and birth. PloS one. 2020 May 12;15(5):e0233114.
4. Rodríguez-Almagro J, Hernández-Martínez A, Rodríguez-Almagro D, Quirós-García JM, Martínez-Galiano JM, Gómez-Salgado J. Women's perceptions of living a traumatic childbirth experience and factors related to a birth experience. International journal of environmental research and public health. 2019 May;16(9):1654.
5. Kalok A, Kamisan Atan I, Sharip S, Safian N, Shah SA. Factors influencing childbirth fear among Asian women: a scoping review. Frontiers in Public Health. 2025 Jan 14;12:1448940.
6. Roux SL. *An exploratory study of mothers perceptions and experiences of an unplanned Caesarean section* (Doctoral dissertation, North-West University).
7. Afaya A, Dzomeku VM, Baku EA, Afaya RA, Ofori M, Agyeibi S, et al. Women's experiences of midwifery care immediately before and after caesarean section deliveries at a public Hospital in the Western Region of Ghana. BMC pregnancy and childbirth. 2020 Dec;20:1-9.
8. Karlström A. Women's self-reported experience of unplanned caesarean section: Results of a Swedish study. Midwifery. 2017 Jul 1;50:253-8.
9. Hollander MH, van Hastenberg E, van Dillen J, Van Pampus MG, de Miranda E, Stramrood CA. Preventing traumatic childbirth experiences: 2192 women's perceptions and views. Archives of women's mental health. 2017 Aug;20(4):515-23.
10. Tong A, Sainsbury P, Craig J. Consolidated criteria for reporting qualitative research (COREQ): a 32-item checklist for interviews and focus groups. International journal for quality in health care. 2007 Dec 1;19(6):349-57.
11. Tolley EE, Ulin PR, Mack N, Robinson ET, Succop SM. Qualitative methods in public health: a field guide for applied research. John Wiley & Sons; 2016 May 9.
12. Bakker W, Zethof S, Nansongole F, Kilowe K, van Roosmalen J, van den Akker T. Health workers' perspectives on informed consent for caesarean section in Southern Malawi. BMC medical ethics. 2021 Dec;22:1-1.
13. Dongyele M, Ansong D, Osei FA, Amuzu EX, Mensah NK, Kwame Owusu A, et al. Communication Medium Used by Clients and Health Professionals in Accessing and Providing

Healthcare in Low Resource Setting: A Descriptive Cross-Sectional Study. Advances in Public Health. 2021;2021(1):7419305.
14. Mwamba C, Mukamba N, Sharma A, Lumbo K, Foloko M, Nyirenda H, et al. Provider discretionary power practices to support implementation of patient-centered HIV care in Lusaka, Zambia. Frontiers in health services. 2022 Sep 14;2:918874.
15. Owino RS, Sewankambo NK, Kituuka O, Kutyabami P. Experiences and ethical issues during shared decision-making in healthcare at rural health facilities in Uganda: An exploratory qualitative study. Research Square. 2024 Sep 16:rs-3.
16. Gourlay A, Wringe A, Birdthistle I, Mshana G, Michael D, Urassa M. “It is like that, we didn't understand each other”: exploring the influence of patient-provider interactions on prevention of mother-to-child transmission of HIV service use in rural Tanzania. PloS one. 2014 Sep 2;9(9):e106325.
17. Orpin J, Puthussery S, Burden B. Healthcare providers’ perspectives of disrespect and abuse in maternity care facilities in Nigeria: a qualitative study. International journal of public health. 2019 Dec;64:1291-9.
18. Meaney S, Lutomski JE, O’Connor L, O’Donoghue K, Greene RA. Women’s experience of maternal morbidity: a qualitative analysis. BMC Pregnancy and Childbirth. 2016 Dec;16:1-6.
19. Richard F, Zongo S, Ouattara F. Fear, guilt, and debt: an exploration of women’s experience and perception of cesarean birth in Burkina Faso, West Africa. International journal of women's health. 2014 May 5:469-78.
20. Olalude OA, Lawal OO, Morhason-Bello IO. Perception and attitude of first-time mothers towards caesarean section in secondary health care facility (A mixed-method study). Journal of West African College of Surgeons. 2025 Apr 1;15(2):224-31.
21. Wang E, Glazer KB, Sofaer S, Balbierz A, Howell EA. Racial and ethnic disparities in severe maternal morbidity: a qualitative study of women's experiences of peripartum care. Women's Health Issues. 2021 Jan 1;31(1):75-81.
22. Colomer-Sánchez A, Ayuso-Murillo D, Lendínez-Mesa A, Ruiz-Nuñez C, Fontán-Vinagre G, Herrera-Peco I. Anxiety effect on communication skills in nursing supervisors: An observational study. Nursing reports. 2021 Apr 1;11(2):207-16.
23. Husby AE, van Duinen AJ, Aune I. Caesarean birth experiences. A qualitative study from Sierra Leone. Sexual & Reproductive Healthcare. 2019 Oct 1;21:87-94.
24. Shulman HC, Dixon GN, Bullock OM, Colón Amill D. The effects of jargon on processing fluency, self-perceptions, and scientific engagement. Journal of Language and Social Psychology. 2020 Oct;39(5-6):579-97.
25. Bullock OM, Colón Amill D, Shulman HC, Dixon GN. Jargon as a barrier to effective science communication: Evidence from metacognition. Public Understanding of Science. 2019 Oct;28(7):845-53.
26. Sharon AJ, Baram-Tsabari A. Measuring mumbo jumbo: A preliminary quantification of the use of jargon in science communication. Public Understanding of Science. 2014 Jul;23(5):528-46.
27. Haskins JL, Phakathi S, Grant M, Horwood CM. Attitudes of nurses towards patient care at a rural district hospital in the KwaZulu-Natal province of South Africa. Africa Journal of Nursing and Midwifery. 2014 Jan 1;16(1):32-44.

Click here to access/download

Supporting Information

S1_ File Interview guide women.pdf

Click here to access/download
Supporting Information
S2 _ File Information sheet and consent form.pdf

Click here to access/download
Supporting Information
S3 _ File POTENTIAL CODES.- WOMENdocx.pdf

Click here to access/download
Supporting Information
S4 _ File PROPOSED HEADINGS.pdf

Click here to access/download
Supporting Information
S5 _ File INTERVIEW GUIDE FOR MEDICAL PRACTITIONERS.pdf

Click here to access/download
Supporting Information
S6 _ File MEDICAL PERSONNEL CODES.pdf
