# Supplementary material for: Women’s experiences of communication with medical staff before and after emergency caesarean birth in Zambia: A qualitative study
Source: PLoS One. 2026 Apr 9;21(4):e0346694. doi: 10.1371/journal.pone.0346694 (PMC13065054; doi:10.1371/journal.pone.0346694)
Supplement: Attachment — Submitted filename: Response to Reviewers.docx [file pone.0346694.s011.docx]

**Response to Reviewers**

**Manuscript ID:** PONE-D-25-47624

**Title:** Women's experiences of communication with medical staff before and after emergency caesarean birth in Zambia: a qualitative study

**Journal:** PLOS ONE

Dear Editor and Reviewers,

We thank you and the reviewers for the constructive and detailed feedback provided. We have carefully addressed all comments and made the necessary revisions. Below, we provide point-by-point responses.

# Response to Journal Requirements

**1. PLOS ONE style requirements:** *Reformatted manuscript to comply with PLOS ONE style.*

**2. Inclusivity questionnaire:** *Completed and uploaded as Supporting Information.*

**3. Data availability contact:** *We have now included the anonymized transcripts as supporting information (see S5 and S7 Files) as well as revised to institutional contact: University of Zambia Biomedical Research Ethics Committee (unzarec@unza.zm). All personal identifiers have been removed to ensure participant confidentiality.*

**4. Abstract consistency:** *Ensured manuscript and online abstracts are identical.*

**5. Figure references:** *Added explicit references to Figure 1 and Figure 2 in the text.*

**6. Recommended citations:** *Added WHO (2018) and Bohren et al. (2015) on respectful maternity care.*

**7. Reference list:** *Reviewed; no retracted articles; added three new references.*

# Response to Reviewer #1

**Comment 1 - Terminology consistency:** Inconsistent use of caesarean birth/delivery.

*Response: Standardised to "caesarean birth" throughout, retaining "delivery and caesarean section" only in direct quotes, referenced titles and anonymized transcripts.*

**Comment 2 - Table 1 formatting:** Needs easier-to-read format.

*Response: Completely reformatted Table 1 to present aggregated summary data by categories (age groups, parity, and education) with counts and percentages.*

**Comment 3 - Private healthcare:** Did participants have the same level of care?

*Response: Thank you for this helpful question. All participants were recruited from the same hospital and received care under the same clinical team and facility protocols. The reference standard (non-fee-paying) and premium wards relates only to where the interview was conducted, not to differences in clinical care received for the condition under study. We did not recruit participants based on ability to pay, insurance status, or use of private healthcare outside the hospital. We also did not collect or analyse private versus non-private healthcare status as a study variable, and the interviews did not aim to compare experiences by payment category. We used private rooms in both ward types to maximize confidentiality and reduce interruptions during interviews. To prevent misinterpretation, we have revised the sentence to clarify this point. Revised wording: To protect confidentiality, interviews were conducted in private treatment rooms or private rooms within the hospital wards, selected for privacy and minimal disruption, rather than to reflect differences in care or payment status. All participants received public healthcare at UTHWNH.*

**Comment 4 - "Starving" to "fasting":** *Changed "starving" to "fasting" as recommended.*

# Response to Reviewer #2

## Abstract

**Add design details:** *Added phenomenological qualitative design and inductive thematic analysis to abstract.*

**Specify key themes:** *Listed all four themes in abstract Results section.*

## Introduction

**Global issues (violence, PTSD):** *Added paragraph on communication failures contributing to obstetric violence, mistrust, and PTSD.*

**Knowledge gap:** *Added explicit statement that no prior study examined this in Zambia's tertiary settings.*

## Materials and Methods

**Sample size justification:** *Added justification citing qualitative guidelines (20-30 for phenomenological studies).*

**Thematic saturation:** *Explained iterative assessment; saturation reached at ~28 interviews; 2 additional to confirm.*

**Who conducted interviews:** *Clarified that the lead author conducted all interviews.*

**Local languages:** *Specified Bemba and Nyanja as the local languages.*

**NVivo version:** *Specified NVivo version 11.*

**Incomplete sentence (Line 153):** *Reviewed and corrected all incomplete sentences.*

## Results

**Systematic theme presentation:** *Restructured with clear thematic headings, sub themes and brief summaries before quotes.*

**Table 1 formatting:** *Reformatted as a summary table with n and %*

**Summary table of themes:** *Added new Table 2 summarising themes and sub-themes.*

## Discussion

**What this study adds:** *Added a paragraph on novel contribution.*

**Systemic reasons:** *Added section on workload, staff shortages, and power dynamics.*

**Respectful maternity care frameworks:** *Linked findings to WHO framework and RMC principles.*

## Strengths and Limitations

**Social desirability bias:** *Added mention of potential bias due to hospital setting.*

**Non-clinician interviewer:** *Highlighted the lead author's non-clinician status as a strength.*

## Conclusion

**Practical implications:** *Revised to include specific practical recommendations for training, language use, and policy.*

## Other Corrections

**Standardised terms:** *Standardised to "caesarean birth" throughout.*

**Grammar ("novel provides"):** *Corrected to "provides novel insights."*

**Spelling ("summaries"):** *Corrected to "summarises."*

**Spacing/line breaks:** *Reviewed and corrected throughout.*

**Note on interview guide attachments:** The interview guide for medical practitioners and codes were attached as supplementary materials for transparency. The manuscript focuses on women's experiences; discussions with healthcare providers were used to counter-check and contextualise the clinical experiences reported by patients as well as understand the hospital operations.

We trust these revisions adequately address the reviewers' concerns. We remain available for any further clarifications.

Sincerely,
**Effie Moreblessing Mphande**
On behalf of all co-author
